# Genetic Basis Underlying Correlations Among Growth Duration and Yield Traits Revealed by GWAS in Rice (*Oryza sativa* L.)

**DOI:** 10.3389/fpls.2018.00650

**Published:** 2018-05-22

**Authors:** Fengmei Li, Jianyin Xie, Xiaoyang Zhu, Xueqiang Wang, Yan Zhao, Xiaoqian Ma, Zhanying Zhang, Muhammad A. R. Rashid, Zhifang Zhang, Linran Zhi, Shuyang Zhang, Jinjie Li, Zichao Li, Hongliang Zhang

**Affiliations:** Key Lab of Crop Heterosis and Utilization of Ministry of Education and Beijing Key Lab of Crop Genetic Improvement, China Agricultural University, Beijing, China

**Keywords:** genetic correlation, genome wide association study, pleiotropic gene, pleiotropic QTL, gene interaction

## Abstract

Avoidance of disadvantageous genetic correlations among growth duration and yield traits is critical in developing crop varieties that efficiently use light and energy resources and produce high yields. To understand the genetic basis underlying the correlations among heading date and three major yield traits in rice, we investigated the four traits in a diverse and representative core collection of 266 cultivated rice accessions in both long-day and short-day environments, and conducted the genome-wide association study using 4.6 million single nucleotide polymorphisms (SNPs). There were clear positive correlation between heading date and grain number per panicle, and negative correlation between grain number per panicle and panicle number, as well as different degrees of correlations among other traits in different subspecies and environments. We detected 47 pleiotropic genes in 15 pleiotropic quantitative trait loci (pQTLs), 18 pleiotropic genes containing 37 pleiotropic SNPs in 8 pQTLs, 27 pQTLs with *r*^2^ of linkage disequilibrium higher than 0.2, and 39 pairs of interactive genes from 8 metabolic pathways that may contribute to the above phenotypic correlations, but these genetic bases were different for correlations among different traits. Distributions of haplotypes revealed that selection for pleiotropic genes or interactive genes controlling different traits focused on genotypes with weak effect or on those balancing two traits that maximized production but sometimes their utilization strategies depend on the traits and environment. Detection of pQTLs and interactive genes and associated molecular markers will provide an ability to overcome disadvantageous correlations and to utilize the advantageous correlations among traits through marker-assisted selection in breeding.

## Introduction

Rice (*Oryza sativa* L.) is the staple food of a large proportion of the world population. Development of elite varieties that use light and soil resources efficiently to produce high yield is an important way to eliminate food shortages resulting from population growth and reductions in the area of arable land (Takeda and Matsuoka, 2008). However, in practice, it is difficult to develop varieties that produce high yields under conditions of shortened growth duration (Okada et al., 2017). Rice breeders also often face the problem of negative correlations among different yield traits (Sandhu et al., 2013; Ranawake and Amarasinghe, 2014), such as grain number per panicle (GNP), panicle number per plant (PN), kilo-grain weight (KGW), and rate of seed setting (Sakamoto and Matsuoka, 2008; Zhao et al., 2016). Many published works suggest that there is a disadvantageous positive correlation between heading date (HD) and GNP, but negative correlations among yield traits (Das and Sarma, 2015; Kharb et al., 2016). Most high-yield varieties have long growth duration in regions with adequate light and energy resources because longer growth duration provides more metabolic activity and nutrition for grain filling (Okada et al., 2017). However, longer growth duration becomes a key limiting factor for widespread production planting, especially in high latitude and high altitude regions. Modern breeding has therefore targeted development of high-yielding varieties with moderate growth duration, such as Shanyou 63 (Zhu et al., 2016) with optimal growth duration of 138 days, 120–130 GNP, medium PN and KGW of 29 g, but also maintains higher yield than those with short growth duration (http://www.ricedata.cn/variety/index.htm). Thus it is feasible to balance the relationships among traits by breaking constraints and to uncover the genetic basis underlying correlations among HD and yield.

Mapping of quantitative trait locus (QTL) has revealed many pleiotropic QTLs (pQTLs) that contributing to the correlations among HD and yield traits at the genomic level. Approximately 734, 209, 239, and 223 widely dispersed QTLs for HD, GNP, PN, and KGW were mapped using different segregating populations respectively (http://www.gramene.org/), which densely distributing across the 12 chromosomes (Ni et al., 2009). By searching their shared markers at those QTLs, we detected more than 27-42 pQTLs per combination of two traits (Supplementary Figure 1 and Supplementary Table 6), namely the common QTLs between two traits. However, only few of them were reported to be pleiotropy by mapping in the same population and in a single environment. For example, one pQTL that contributed to the phenotypic variations of both HD and GNP was detected using a recombination inbred line derived from the cross Zhenshan 97B × Miyang 46 (Zhuang et al., 2002). However, most of the other pQTLs detected are not yet to be confirmed by mapping in the same population and in a single environment.

With increasing numbers of QTLs being cloned (Supplementary Tables 11–14), several pleiotropic genes (designated pGenes, the genes with pleiotropy for different traits of interest, that is, commonly associated with the traits of interest in GWAS results) related to the four traits in pQTLs have become evident. Among them, *Ghd7, DTH8*, and *RFL* have important roles in regulation of HD and GNP (Rao et al., 2008; Xue et al., 2008; Wei et al., 2010). *AID1* and *RFL* are pGenes that control HD and PN. *AID1* mutant plants have fewer tillers and show delayed flowering by up to 15 days compared to the wild type (Zhu et al., 2004). The expression level of *RFL* affects HD and vegetative axillary meristems (Rao et al., 2008). *HGW* has a fundamental role in the ubiquitination pathway in control of HD and KGW (Li et al., 2012). *LAX1, OsSPL14* and *RFL* affect GNP and PN by having opposite roles in production of tillers and panicle branches (Komatsu et al., 2003; Rao et al., 2008; Jiao et al., 2010; Miura et al., 2010). *GSD1* is an important regulator of GNP and KGW, with a role in regulating photoassimilate translocation through the symplastic pathway to impact grain setting (Gui et al., 2014). The literature about related to pQTLs and pGenes suggests that pleiotropic effects among agronomic traits are very common, but study on its discovery and our understanding of the genetic basis is still quite limited.

Facilitated with high-throughput sequencing technology, genome-wide association study (GWAS) using high quality single nucleotide polymorphisms (SNPs) has been proved to be a good approach to explain the genetic basis of complex quantitative traits (Huang et al., 2010, 2011). It may also provide an efficient tool to discover the genetic basis of correlations among agronomic traits since GWAS has demonstrated the advantages in improving the efficiency of detecting natural variations, and in saving time for QTL mapping and gene cloning, and especially in detecting QTLs controlling multiple agronomic traits in different environments (Myles et al., 2009). In this study, we estimated the phenotypic correlations among HD and yield traits for different rice subspecies in two typical environments using a representative mini core collection (MCC) of the global cultivated rice (Zhang et al., 2011), and carried out GWAS in *indica, japonica* and the full populations using high quality SNPs from the whole genome. Our aim was to identify QTLs simultaneously controlling growth duration and yield traits, to analyze the genetic bases contributing to correlations among them, and to investigate possible utilization pattern for the QTLs with different genetic bases in breeding practice so as to gain from the positive correlation or avoid the negative correlation. The study sheds light on understanding of the genetic basis underlying the correlations among four traits and provides QTLs and markers to breeders for further improving the important agronomic traits that are subject to both positive and negative correlations.

## Materials and methods

### Populations for GWAS

The 266 rice varieties of the MCC for GWAS included 87 non-Chinese cultivated varieties that represented a global collection of more than 9,000 foreign rice accessions, and 179 varieties selected from more than 50,000 Chinese varieties conserved in the Chinese National Gene Bank, and representing more than 70% of the genetic diversity in the respective germplasm sets (Zhang et al., 2011). The MCC thus has a wide geographic and morphological representation (Supplementary Table 1 and Supplementary Figure 2A). It comprises 151 landraces, 114 improved varieties from 24 provinces in China and 35 other countries; it covers all maturity types, including 70 early-season, 100 intermediate-season, and 87 late season varieties.

The phylogenetic relationships of the 266 varieties were determined using genetic distance calculated from 1.8 million high quality SNPs, and the resulting neighbor-joining tree (Supplementary Figure 2B) as well as the principal-component analysis (PCA) (Supplementary Figure 3) showed that they comprised two divergent subspecies groups, *Oryza sativa ssp*. *indica* with 157 varieties, and *ssp*. *japonica* with 109 varieties.

### Phenotyping for MCC

Phenotypes of MCC were evaluated in two rice-growing environments (Supplementary Figure 4); one was grown from November 2012 to April 2013 at Sanya (SY) in Hainan province (18°20′N), representing a short-day environment; the other was grown from May to October 2013 at Changsha (CS) in Hunan province (28°11′N), representing a long-day environment. All varieties were planted in a randomized complete block design with two replications. Strong and uniform seedlings were transplanted according to a standard design of three rows × 10 plants / row, with a relatively low density of 150,000 plants per hectare so as to avoid interaction between density and genotype. Field management, including irrigation, fertilization, and disease and pest control, followed local plot trial management.

The middle five plants in the middle row for each variety were sampled for phenotyping that included HD and the three major yield factors, PN, GNP and KGW. HD of each accession was recorded when the first inflorescence of more than 50% of plants had emerged above the flag leaf sheath; GNP was the mean of grains number on the main panicles of five plants; PN was the mean number of productive panicles from the five plants; and KGW was estimated from the weights of 100 fully filled grains.

### Genome sequencing and SNP identification

As a subset of the 3,000 Rice Genomes Project (3KRGP), the whole genome sequence of the entire MCC, was carried out using high-throughput sequencing on the HiSeq2000 platform of BGI-Shenzhen (Li, 2014; Alexandrov et al., 2015; Sun et al., 2017). Clean reads showed an average sequencing depth of 14 ×, covering 94.0% of the Nipponbare reference genome (Kawahara et al., 2013). Using the same method as 3KRGP, we called 10.7 million non-redundant SNPs in the MCC. By quality control, we obtained 4.6 million high quality SNPs with minor allele frequency ≤ 5% and missing rate ≤ 50% across the 266 accessions, 12.35 SNPs per kb in the rice genome on average approximately.

### GWAS and QTL detection

GWAS for *indica, japonica* and full populations on traits HD, GNP, PN and KGW were separately implemented using the compressed mixed linear model (CMLM) that took into account population structure estimated by principal component (PC) and kinship so as to reduce false positives (Kang et al., 2008; Price et al., 2010; Zhang Z. et al., 2010; Yang et al., 2014). The principal component (PC) and kinship were conducted using the 4.6 million SNPs by an R implementation (www.R-project.org) available as part of the GAPIT software package (version 3.0.2) (Lipka et al., 2012).

Given that most of multi-test adjustment methods especially the Bonferroni correction are too stringent because not all markers are independent due to linkage disequilibrium (LD) among markers, missing QTL with small effect usually makes GWAS not as straightforward as previously thought (Liu et al., 2016). Instead of multi-test adjustment method, in this study we used the permutation test, which was proven to be an effective strategy to define the genome-wide significant threshold of a complex agronomic trait controlled by multiple alleles with minor effect, and had better control of population structure (Huang et al., 2015; Yano et al., 2016). We divided the phenotype (Y) of each accession into the original genotypic effect (G) and the fixed effect of population structure (Ps). Ps was estimated by the average effect of each PC on each individual through regression analysis of each PC on Y; the remainder was G after excluding Ps from Y. G was randomly reshuffled as Gr, and the new phenotype of each accession was Ps + Gr. We executed permutation test using CMLM with the same parameters of GWAS, a total of 1,000 sets of Ps + Gr were performed for the four traits with the same number of PC. To improve the computational efficiency, the SNPs whose *P*-values were greater than 2 in the GWAS using original phenotypes were included in the permutation test. Any SNP whose -log_10_(*P*) was higher than -log_10_(*P_perm*), where *P_perm* was the lowest *P*-value in 1,000 permutation tests, was considered to be real association between the SNP and the original phenotype in the GWAS.

LD length in cultivated rice is approximately 120 and 170 kb for *indica* and *japonica*, respectively (Mather et al., 2007; Huang et al., 2010). We therefore defined one QTL by the bin that included at least three significant SNPs with a physical distance <70 kb between two adjacent significant SNPs.

### Hitch-hiking effects among SNPs within pQTL on phenotypic correlation

To investigate the role of LD on phenotypic correlation (such as HD vs. GNP) through hitch-hiking effects of QTL controlling one trait (such as HD) with other traits (such as GNP), we firstly calculated the average *r*^2^ (LD) of all pairs between significant SNPs in HD QTL and significant SNPs in GNP QTL using HAPLOVIEW, the detail is demonstrated in the schematic diagram (Supplementary Figure 11A). We calculated the Pearson correlation coefficient (*r*_HD2GNP_) between average *r*^2^ of LD and differences between phenotypic variance contributions (PVC) to GNP for HD QTL and GNP QTL (i.e., PVC to GNP for significant SNPs within GNP QTL in GNP GWAS minus PVC to GNP for significant SNPs within HD QTL in GNP GWAS). This *r*_HD2GNP_ thus accounted for the hitch-hiking effect of HD QTL on GNP. Similarly, we also calculated the hitch-hiking effect of GNP QTL on HD QTL, *r*_GNP2HD_, and other combinations.

### Effects of SNP or haplotypes and their distribution among different variety types

We investigated the significance of effects on related traits of alleles of each pleiotropic SNP (pSNP; that is, a SNP simultaneously associated with two different traits), haplotypes of pQTLs, and interactive genes (iGenes, the genes that simultaneously control different traits of interest by interaction among those genes) by multiple comparison analysis of GLM in SPSS (version 19.0). Alleles with significantly higher effect on some traits such as HD were denoted as increased effect alleles for that trait, and those with significantly lower effect were denoted as decreased effect alleles. For pSNPs or haplotypes, for example, pleiotropy between HD and GNP, we classified their effects into four types: the same direction (SD) with both increased effect on HD and GNP, or SD with both decreased effect on HD and GNP, the opposite direction (OD) with increased effect on HD but decreased effect on GNP, or OD with decreased effect on HD but increased effect on GNP. We then calculated the distribution of alleles with different kinds of effects among four types of varieties, including *indica* landraces, improved *indica* varieties, *japonica* landraces, and improved *japonica* varieties.

## Results

### Phenotypic variation and correlation

**Figure 1:**
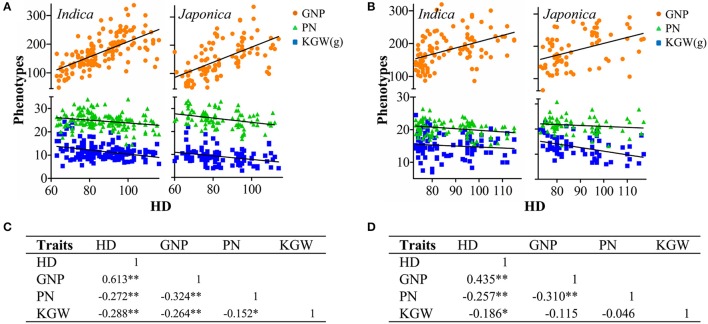
Phenotype distribution of grain number per panicle (GNP), panicle number (PN) and kilo-grain weight (KGW) along with increase of heading date (HD) in different subspecies at Sanya **(A)** and Changsha **(B)**, and correlations among the four traits at Sanya **(C)** and Changsha **(D)**. ^*^, significant correlation at the 0.05 level (two tails); ^**^, significant correlation at the 0.01 level (two tails).

Phenotypes of the four traits for the whole MCC and two subspecies at SY and CS showed continuous Gaussian distribution (Supplementary Figure 5) and wide variation. The coefficient of variation at SY was higher than that at CS; however, the phenotypic means for the MCC indicated longer HD, higher GNP and PN, and lower KGW at CS than at SY. For subspecies (Supplementary Table 2), *japonica* had longer HD, more GNP and PN, and lower KGW than *indica* in both environments. Pearson's correlation analysis (Figure 1 and Supplementary Table 3) indicated that in general the correlations among traits were stronger at SY than that at CS. This may be attributed to the smaller range of variation in HD due to high temperature at CS, but some correlations also appeared as subgroup-specific or environment-specific. There was a significant positive correlation between HD and GNP and a significant negative correlation between GNP and PN in all populations and environments. Longer HD significantly decreased PN and KGW in both subspecies at SY, but its impact on these two traits depended on subspecies at CS, where HD was negatively correlated with KGW, but not PN in *indica* and the reverse in *japonica*. The interplay between GNP and KGW depended on subspecies, being significantly negative only in *japonica*. The interplay between PN and KGW was very weak, being only significantly negative in *indica* at SY. These findings implicate the genetic foundation underlying phenotypic correlation, which was also impacted by environment to some extent.

### Detection of pQTL by GWAS

**Figure 2:**
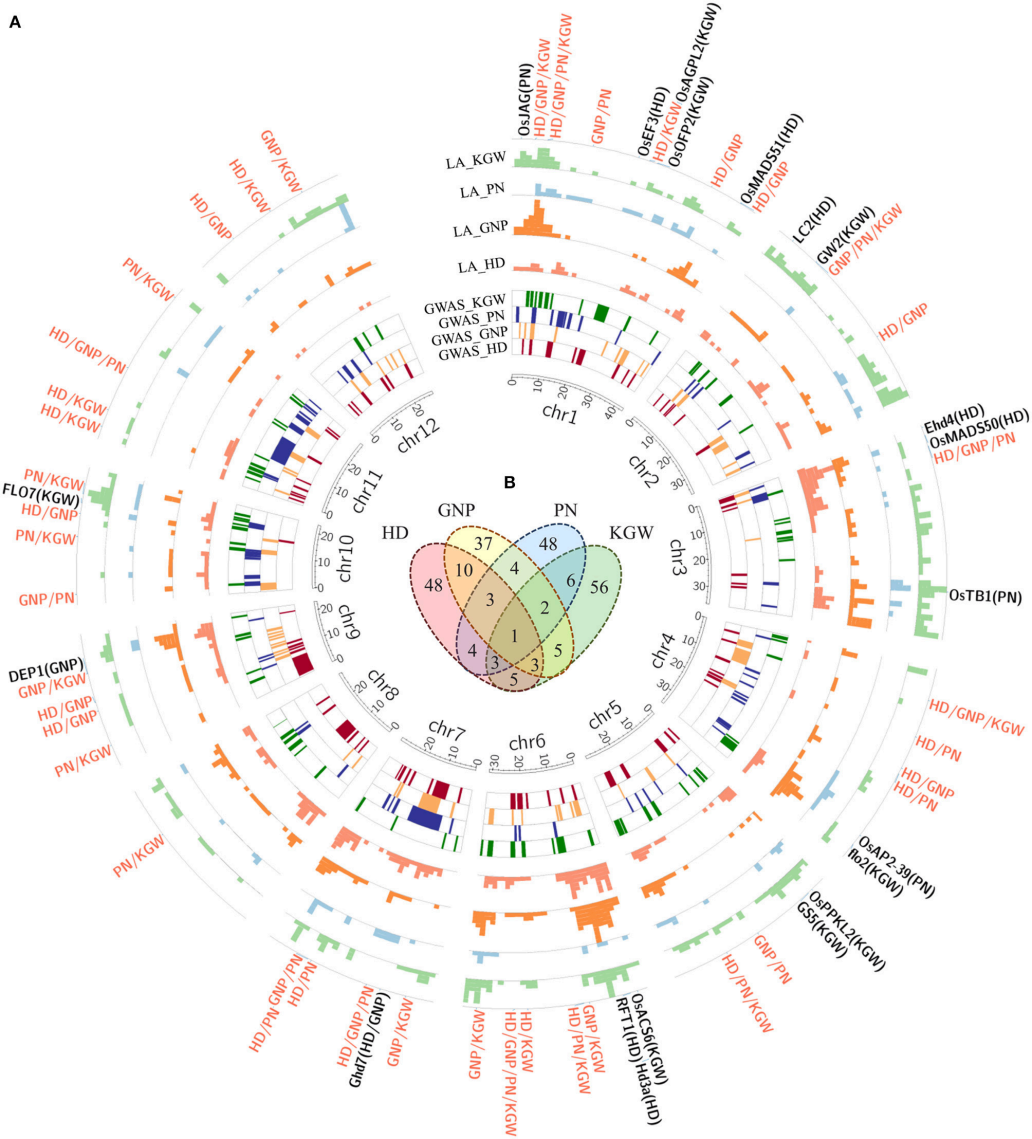
Display of pleiotropic quantitative trait loci (QTLs) identified in the genome-wide association study (GWAS). **(A)** Circos diagram illustrates QTLs contributing to different traits from GWAS and linkage analysis (LA). Here, the inner four layers from the inside represent QTLs for HD, GNP, PN, and KGW, respectively; the outer four layers represent the frequency of each bin for HD, GNP, PN and KGW covered by LA-QTL respectively; the outside red fonts represent pleiotropic QTLs and the black fonts represent cloned genes. **(B)** Venn diagram of pleiotropic QTLs from GWAS among the four traits in same population and environment.

GWAS was performed to dissect the underlying genetic basis of correlations among the four agronomic traits for the full population (266 lines) and each subspecies, the differences of which contributed to 94% of population structure and differentiation using CMLM (Huang et al., 2010; Lipka et al., 2012; Figure 2A and Supplementary Figures 6–9). With the permutation test threshold to evaluate significant SNP, we identified 77, 65, 71 and 81 QTLs associated with HD, GNP, PN and KGW, respectively (Supplementary Tables 4, 5), and the reported QTL clusters had high coverage percentages to GWAS QTL (Figure 2A and Supplementary Table 6). Among them, only a few QTLs were repeatedly detected in both environments, which had 2, 6, 3, 7 QTLs for HD, GNP, PN, KGW in the full population, 4, 1, 0, 1 QTLs in *indica*, and 0, 3, 1, 1 QTLs in *japonica*, respectively. The low proportion of common QTLs between SY and CS was partly ascribed to the different light and temperature conditions. The number of QTLs in *indica* was higher than in *japonica*, and was consistent with lower variation in *japonica* than in *indica* (Garris et al., 2005). Within these QTLs, 6 cloned genes were significantly associated including *OsMADS50, RFT1, Hd3a* regulating HD, *TUT1* relating to PN, and *OsPPKL2, FLO7* involved in KGW, and a further 8, 2, 4, and 8 cloned genes located in the scope of QTLs.

**Figure 3:**
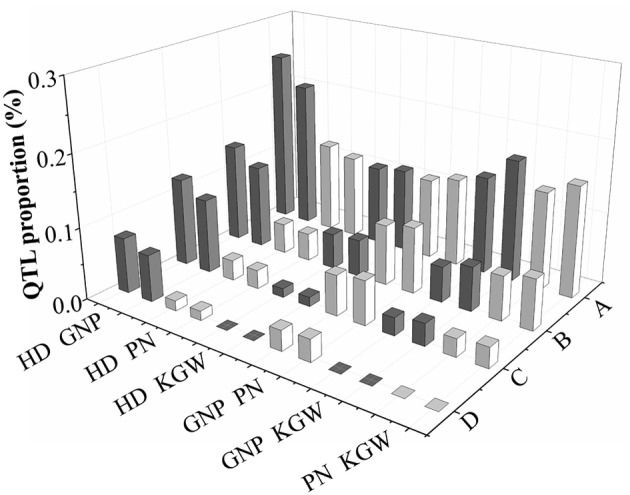
Percentages of pleiotropic QTLs for different combinations relative to the total QTL number of each corresponding trait revealed by GWAS. Here, “A”, all pleiotropic QTLs in the same population and environment; “B”, pleiotropic QTLs with *r*^2^ of Llinkage disequilibrium (LD) > 0.2; “C”, pleiotropic QTLs containing pleiotropic genes; “D”, pleiotropic QTLs containing pleiotropic genes with pleiotropic SNPs. Two bars in each pair of traits (such as HD GNP) in “D” and elsewhere are percentages of pleiotropic QTLs relative to the QTLs of the first trait (such as HD) and the second trait (such as GNP), respectively.

There were 46 pQTLs, each of which was shared in GWAS by two different traits in the same population and environment (Figure 2B and Supplementary Figure 10). Among them, 16, 9, 9, 8, 11, 11 pQTLs were detected between HD and GNP, HD, and PN, HD and KGW, GNP and PN, GNP and KGW, and PN and KGW, respectively. There was high correspondence between pQTL number and the degree of phenotypic correlation among traits. For example, the number of pQTLs between HD and GNP was the highest (Figure 3), corresponding to the highest correlation between HD and GNP. These pQTLs may shed light on the genetic basis underling the correlations among traits.

### Genetic analysis of pQTL

There were at least two genetic bases to explain the pleiotropic phenomenon: pGene (Rao et al., 2008; Miura et al., 2010; Li et al., 2012; Lu et al., 2012) or linkage of genes controlling different traits (Bai et al., 2011; Luo et al., 2013). To distinguish these two fundamental genetic bases, we annotated all significant SNPs, and found 47 pGenes in 15 pQTLs, 18 pGenes containing 37 pSNPs in 8 pQTLs, while other 38 pQTLs did not contain any pSNP. We hardly distinguished the real pGenes from the linkage between genes when two pGenes were found in one QTL, unless functions of all pGenes were validated. Here we focused on two sets of genes or SNPs, one set was the cloned pGenes and pSNPs within pQTLs and the other was pQTLs with unknown pGenes or without any pGene or pSNP, in order to investigate the contribution of pGenes or pSNPs and the contribution of linkage between different genes or SNPs within pQTLs to the phenotypic correlations among traits and their utilization in breeding.

**Figure 4:**
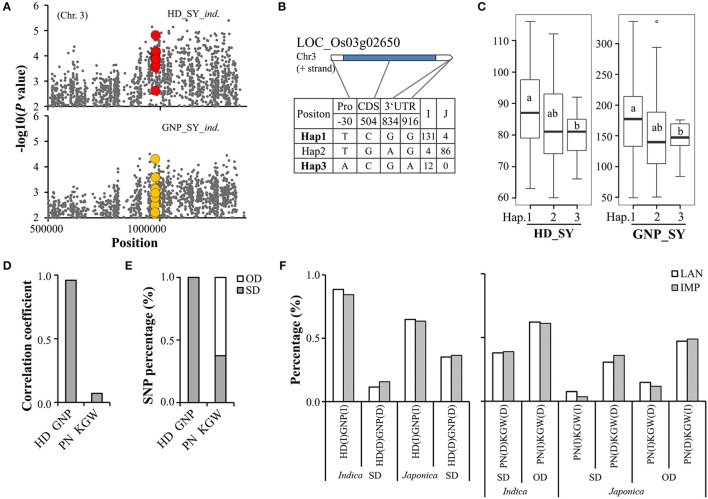
Analysis of pleiotropic genes and SNPs. **(A)** Local Manhattan plots from 450,000 to 1,450,000 on Chromosome 3 for GWAS of HD and GNP in *indica* (*ind*.) at Sanya (SY). Here, colored dots represent SNPs in pleiotropic gene *HYR* (LOC_Os03g02650). **(B)** Gene structure of *HYR* and its haplotype distribution between the two subspecies. **(C)** Differences for HD and GNP at SY among different haplotypes in pleiotropic gene *HYR*, different letters in box plots label significant differences (*P* < 0.05) using Duncan's test. **(D)** Correlations of effects calculated using pleiotropic SNPs among different traits. **(E)** Haplotype percentages of pleiotropic SNPs with SD or OD. **(F)** Distribution of pleiotropic SNPs with SD or OD in landrace (LAN) and improved varieties (IMP) for HD and GNP, PN and KGW in *indica* and *japonica*. I, increased effect; D, decreased effect; SD, same direction effect; OD, opposite direction effect.

Among the 37 pSNPs (Supplementary Tables 7, 8), the most pSNPs and pGenes were between HD and GNP (26 pSNPs and 12 pGenes containing pSNPs) in 5 pQTLs, followed by PN and KGW (8 and 4), and GNP and PN (2 and 2) in 1 and 2 pQTLs. The pQTLs between HD and GNP accounted for 6.49 and 7.69% of the QTLs for HD and GNP), respectively, pQTLs between PN and KGW accounted for 1.41 and 1.25%, and pQTLs between GNP and PN accounted for 3.08 and 2.82%, respectively. Among the 47 pGenes (Supplementary Tables 7, 8), the most pQTLs and pGenes were between HD and GNP (8 pQTLs containing 22 pGenes), followed by GNP and PN (4 and 15), PN and KGW (2 and 7), HD and PN (2 and 5), GNP and KGW (2 and 2), and HD and KGW (1 and 1). The pQTLs between HD and GNP accounted for 10.39 and 12.31% of the QTLs for HD and GNP, respectively, pQTLs between GNP and PN accounted for 6.15 and 5.63%, pQTLs between PN and KGW accounted for 2.82 and 2.47%, pQTLs between HD and PN accounted for 2.60 and 2.82%, pQTLs between GNP and KGW accounted for 3.08 and 2.47%, and pQTLs between HD and KGW accounted for 1.30 and 1.23%. The number of pQTLs containing pSNPs and pGenes represented high correspondence with the strength of phenotypic correlation (Figure 3). Among these pGenes, *Ghd7* and *HYR* were cloned. *HYR* (LOC_Os03g02650) was reported to control the chlorophyll levels and chloroplast number, and to further affect plant biomass including GNP and PN (Ambavaram et al., 2014). In our study, *HYR* was pleiotropy between HD and GNP (Figure 4A). Haplotype analysis (Figures 4B,C) indicated that this gene clearly differentiated between the two subspecies, that Hap1 and Hap 3 mainly presented in *indica* and Hap2 was mainly in *japonica*. The varieties carrying Hap1 showed significantly longer HD and more GNP than Hap3, possibly explaining the positive correlation between HD and GNP. *Indica* varieties grow in the low latitudes where their growth is not limited by the light and energy resources (Londo et al., 2006), thus the Hap1 of *HYR* was mainly selected in *indica* in order to sufficiently make use of the long season to increase yield, especially GNP. In contrast, *japonica* varieties grow in the higher latitudes where their growth duration is usually limited (Londo et al., 2006), consequently Hap2 of *HYR* was mainly selected in *japonica* to maintain a balance between HD and GNP. The effect of pSNPs (Figures 4D,E) showed that the percentage of pSNPs with SD was 100% for HD vs GNP, completely explaining the strong positive phenotypic correlation between HD and GNP. But for PN vs KGW, 62.50% pSNPs appeared OD effect, being consistent with the moderate strength of negative phenotypic correlation between PN and KGW. The distribution of pSNPs with SD or OD in landraces and improved varieties (Figure 4F) indicated that genotypes with earlier HD and fewer GNP were higher in proportion in *japonica* than in *indica*. The subspecific differentiation attributed to the adaptions of two subspecies to different light and energy resources (Khush, 1997; Garris et al., 2005), that is, *japonica* has decreased its growth duration in order to ripen normally at the cost of decreasing GNP to some extent. For pSNPs between PN and KGW, breeders tended to select the genotypes with more PN but lower KGW in *indica*, but with the reverse situation in *japonica*.

**Figure 5:**
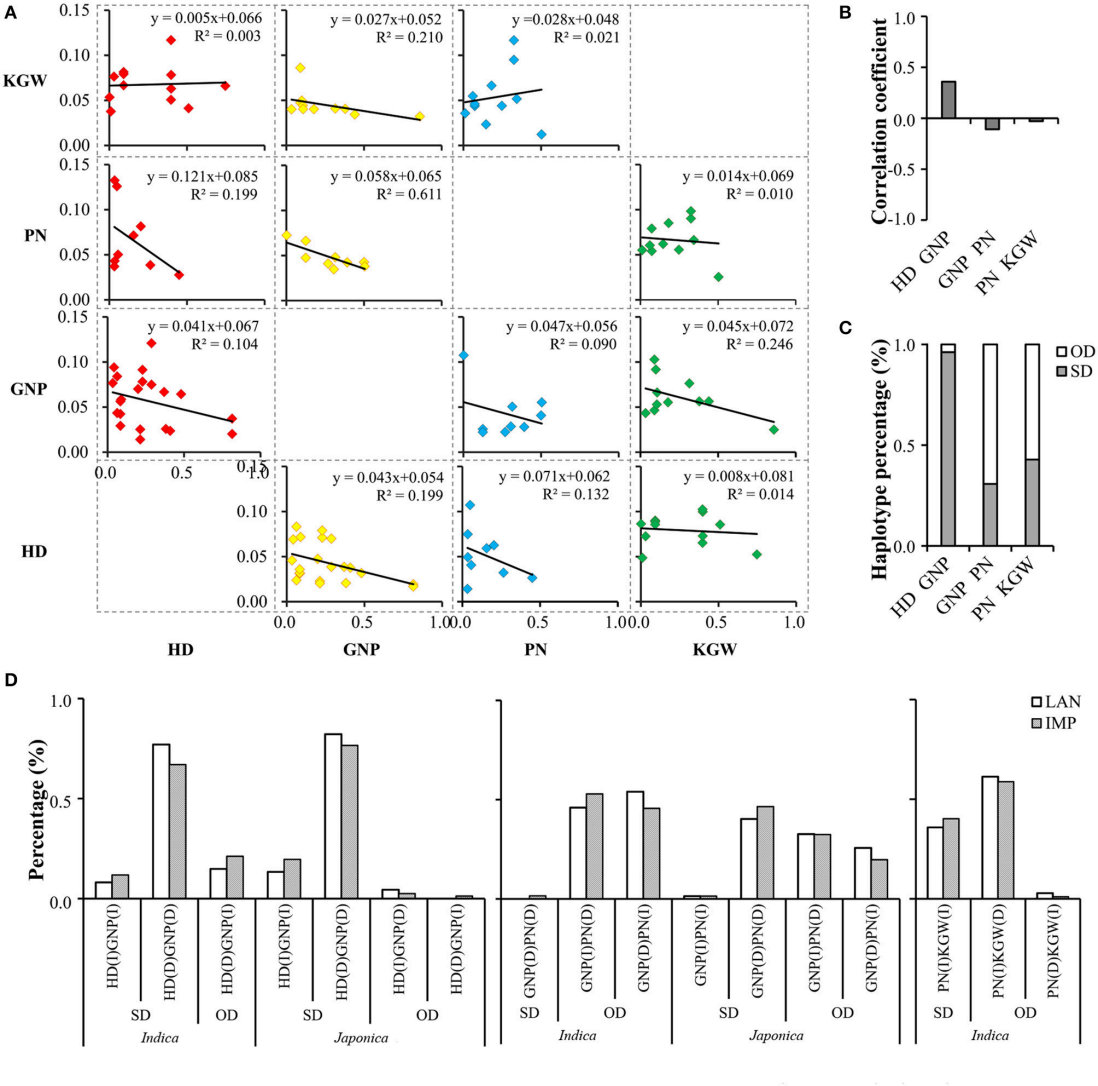
Analysis of pleiotropic QTLs. **(A)** Correlations between LD of two adjacent QTLs and differences of phenotypic variance contributions (PVC). Red, yellow, blue and green represent the traits of calculated PVC respectively, and each combination has two corresponding results of correlation analysis. **(B)** Correlations of effect which were calculated using pleiotropic QTLs (*r*^2^ of LD > 0.2) among different traits. **(C)** Haplotype percentage of pleiotropic QTLs (*r*^2^ of LD > 0.2) with SD or OD. **(D)** Distribution of pleiotropic QTLs (*r*^2^ of LD > 0.2) with SD or OD in LAN and IMP for HD and GNP, GNP and PN, PN and KGW in *indica* and *japonica*. I, increased effect; D, decreased effect; SD, same direction effect; OD, opposite direction effect; LAN, landrace varieties; IMP, improved varieties.

To estimate the effect of linkage between genes or QTLs on phenotypic correlation between pairs of traits, we calculated the average *r*^2^ (LD) between QTLs of two traits belonging to the same pQTL. Among 46 pQTLs, the LD of approximately 5% of combinations exceeded 0.6 (Supplementary Figure 11B). The number of pQTLs indicated the consistent trend of distribution among six combinations at each of four *r*^2^ (LD) levels (> 0.2, > 0.4, > 0.6 and > 0.8) (Supplementary Figure 11C). Among 27 pQTLs with LD stronger than 0.2, there were 9, 3, 4, 6, 4, and 5 pQTLs in combinations between HD and GNP, HD and PN, HD and KGW, GNP and PN, GNP and KGW, PN and KGW, respectively. pQTLs between HD and GNP with LD stronger than 0.2 accounted for 11.69 and 13.85% of the QTLs for HD and GNP, respectively; those between HD and PN accounted for 3.90 and 4.23%, those between HD and KGW accounted for 9.23 and 8.45%, those between GNP and PN accounted for 6.15 and 4.94%, those between GNP and KGW accounted for 5.19 and 4.94%, and those between PN and KGW accounted for 7.04 and 6.17%, respectively. These percentages represented roughly the strength of phenotypic correlation between the corresponding traits. The correlation analysis (Figure 5A) between PVC differences and *r*^2^ (LD) of pQTLs showed that the interplay influence decreased along with the LD decay in one pQTL, indicating the contribution of LD to the phenotypic correlation. The effect of these pQTLs on different traits (Figures 5B,C) indicated that 96.15% of haplotypes showed SD effects for HD vs GNP, only 30.77 and 75% of haplotypes showed OD effects for GNP vs PN and PN vs KGW, implying their weaker contributions to phenotypic correlation than pSNPs. The distribution of haplotypes with SD or OD in landraces and improved varieties (Figure 5D) revealed the possibility of breaking the phenotypic correlations by recombination, which may result to produce haplotypes with earlier HD but increased GNP, and haplotypes with more PN and higher KGW simultaneously. The above results indicated that linkage between genes for different traits contributes partially to the phenotypic correlation, but their constraints on improving different traits simultaneously can be broken by recombination.

### Shared pathways contribute to phenotypic correlation

**Figure 6:**
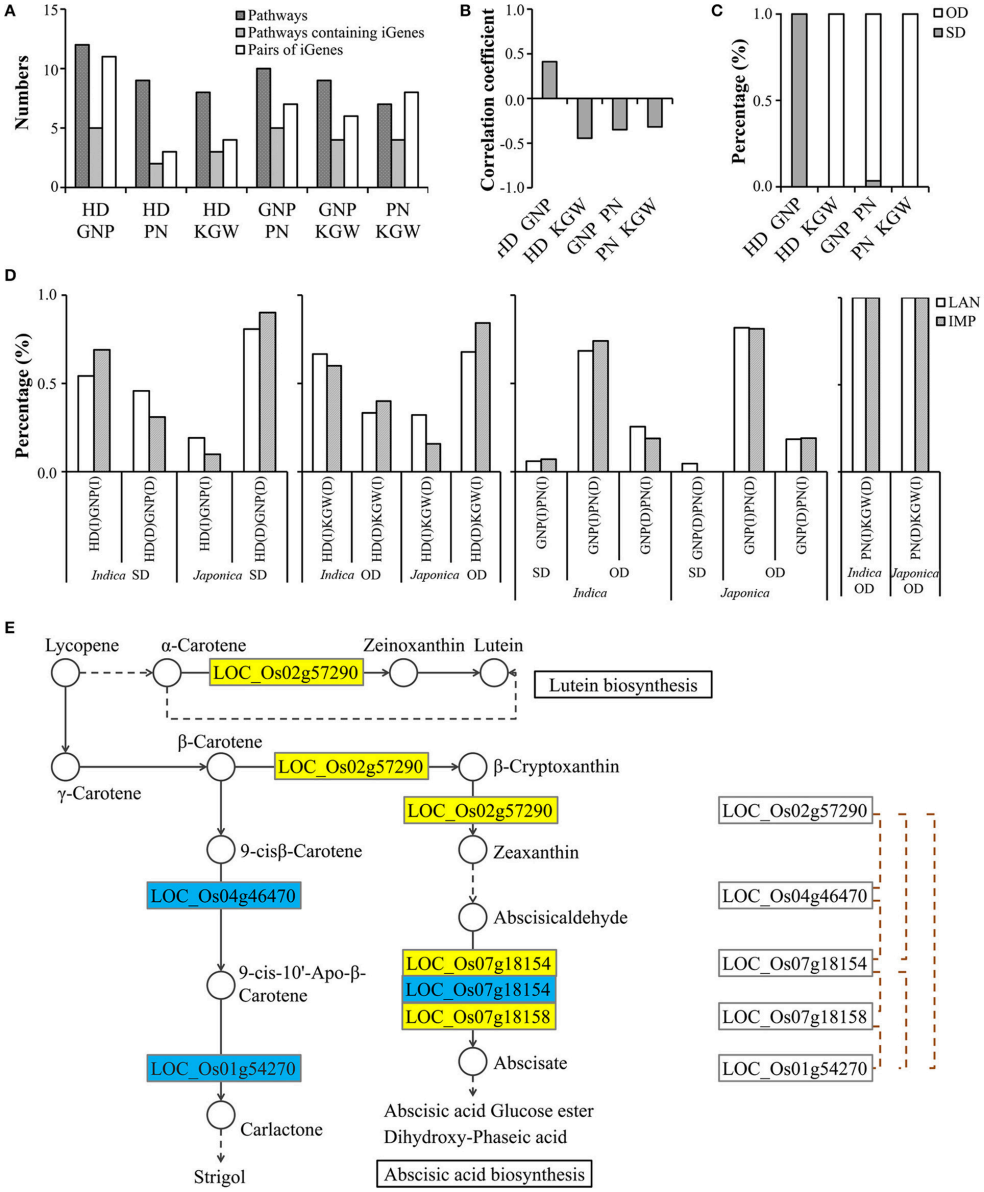
Analysis of shared pathways containing interactive genes. **(A)** Numbers of pathways, pathways containing interactive genes and pairs of interactive genes detected by GWAS. **(B)** Correlations of effect which were calculated using interactive genes among different traits. **(C)** Haplotype percentages of interactive genes with SD or OD. **(D)** Distribution of interactive genes from pathways with SD or OD in LAN and IMP for HD and GNP, HD and KGW, GNP and PN, GNP and KGW in *indica* and *japonica*. **(E)** Pleiotropy of genes regulating GNP (yellow) and PN (blue) by genetic interaction in the partial sketchy pathway of carotenoid biosynthesis based on the KEGG database (http://www.kegg.jp/), including in known genes and candidate genes from GWAS. Black solid arrows, direct synthetic steps; black dotted arrows, indirect synthetic steps; brown dotted lines, significant interactions among participating genes; I, increased effect; D, decreased effect; SD, same direction effect; OD, opposite direction effect; LAN, landrace varieties; IMP, improved varieties.

In addition to pQTL, it was reported that interaction between D53 and IPA1 in the strigolactone signal pathway can simultaneously control plant height and tiller number in rice (Song et al., 2017). We investigated the contribution of genetic interaction among genes involved in the same pathway to the phenotypic correlation. By pathway searching in the KEGG database (http://www.kegg.jp/) for all cloned genes and significantly associated genes related to the four traits, we discovered 15 pathways in which two or more genes were involved in regulation of multiple traits and in which at least one of them was cloned. In the 15 pathways (Supplementary Table 9) there were 75 new candidate genes (14 for HD, 28 for GNP, 15 for PN, and 18 for KGW) and 27 cloned genes. These pathways included arginine and proline metabolism, biosynthesis of amino acids, carbon fixation in photosynthetic organisms, carbon metabolism, carotenoid biosynthesis, circadian rhythm-plant, diterpenoid biosynthesis, glycolysis-gluconeogenesis, nitrogen metabolism, plant hormone signal transduction, plant-pathogen interaction, porphyrin and chlorophyll metabolism, starch and sucrose metabolism, ubiquitin mediated proteolysis and zeatin biosynthesis. Through interaction analysis among genes within the same pathway (Supplementary Table 10), we detected 39 pairs of iGenes in 8 pathways, including 11 iGenes in 5 pathways for HD and GNP, 3 in 2 for HD and PN, 4 in 3 for HD and KGW, 7 in 5 for GNP and PN, 6 in 4 for GNP and KGW, and 8 in 4 for PN and KGW. The higher number of iGenes and pathways represented a trend with stronger correlations among traits (Figure 6A), and apparently indicating their contribution to phenotypic correlations among the four traits. For example, in the carotenoid biosynthetic pathway (Figure 6E) we found that *OsCCD7* (LOC_Os04g46470) and *CYP97A4* (LOC_Os02g57290) interactively control PN and GNP by determining the production of carotenoid, i.e., strigolactone or abscisic acid. It has been shown that ABA is a regulator of strigolactone biosynthesis (López-Ráez et al., 2010; Cheng et al., 2017). *OsCCD7* (LOC_Os04g46470) was reported to negatively control tiller number, PN and GNP (Kulkarni et al., 2014), *CYP97A4* was expressed in the root and stalk junction from which tillers emerge (Lv et al., 2012). Data in the RiceXpro database (http://ricexpro.dna.affrc.go.jp/) also showed high expression level of *CYP97A4* in inflorescences of Nipponbare. The above results suggest that *OsCCD7* (LOC_Os04g46470) and *CYP97A4* (LOC_Os02g57290) might jointly control GNP and PN by interaction in the carotenoid biosynthetic pathway. The effect of haplotypes for each pair of iGenes on the traits (Figures 6B,C) indicated that 100.00% of haplotypes were SD for HD vs. GNP, and 100, 96.55, and 100% of haplotypes were OD for HD vs KGW, GNP vs. PN, and PN vs. KGW. These results implied that the contribution of iGenes to phenotypic correlation was higher than pQTLs with *r*^2^ LD > 0.2. The distribution of haplotypes with SD or OD in landraces and improved varieties (Figure 6D) indicated that breeders tended to select genotypes with longer growth duration with a hope to increase GNP of *indica* varieties grown at low latitudes, but select genotypes with shorter growth duration so as to guarantee timely maturity of *japonica* varieties grown at high latitudes (Londo et al., 2006). Moreover, breeders preferred the haplotypes with more GNP and fewer PN when the effects of their haplotypes were opposite direction, because increased planting density can compensate for disadvantages of fewer PN. Consequently, only a small proportion of varieties containing the haplotypes in iGenes that increase both GNP and PN.

## Discussion

A common phenomenon in crop production is phenotypic correlations among agronomic traits. Many of these phenotypic correlations are constraints to crop production and breeding progress. For example, the positive correlation between HD and yield traits usually limits planting region or reduces the production efficiency in regions with restricted light and heat resources, and the negative correlations among yield traits have been the critical constraints when yield reach certain levels. Using the diverse rice MCC and whole genome sequence, we investigated the possible genetic basis underlying correlations among HD and yield traits based on GWAS and interaction analysis. Our results provided not only more information about pSNP, pGene, or pQTL, but also theoretical guidance to develop the varieties with short growth duration and high yield.

### Genetic bases of correlations among HD and yield traits

Previous study supported at least two genetic bases underlying correlations among traits in rice, i.e., pGenes (Supplementary Figures 12–15) and linkage between two genes controlling different traits (Bai et al., 2011; Luo et al., 2013). Our results not only confirmed the two genetic bases, but also suggested a third one, i.e., the interaction between genes within the same biological pathway. Although it was not definitively reported that interactive genes within the same biological pathway contribute to correlations between traits, Song et al. (2017) reported that interaction between D53 and IPA1 in the strigolactone signal pathway can simultaneously control the plant height and tiller number. We identified 39 pairs of iGenes in 8 pathways contributing to phenotypic correlation among growth duration and yield traits. Among those pathways five contain at least five candidate genes involved in controlling correlations among traits by gene-gene interaction. These five pathways involved two classes of metabolism, one related to energy metabolism, including carbon metabolism, glycolysis / gluconeogenesis, and starch and sucrose metabolism; the other one related to plant hormones, including plant hormone - signal transduction and carotenoid biosynthesis. Energy metabolism is fundamental for development of almost all agronomic traits, and thus contributes to phenotypic correlations among traits. For example, the *Moc2* mutant has significantly reduced tiller numbers and a dwarf phenotype; *Moc2* encodes cytosolic fructose-1,6-bisphosphatase 1 (FBP1), which is a key enzyme in the sucrose biosynthesis pathway (Koumoto et al., 2013). Several genes involving in plant hormone biosynthesis and signal transduction are reported to control different traits. For example, *D10* encodes a carotenoid cleavage dioxygenase and functions in strigolactone biosynthesis, and can regulate the tiller number and plant height simultaneously (Zhang S. et al., 2010). Our results indicated that *OsCCD7* (LOC_Os04g46470) and *CYP97A4* (LOC_Os02g57290) might together control GNP and PN by interaction in carotenoid biosynthetic pathway. The contributions of these three genetic bases to correlations among traits varied. Percentages of SD and OD indicated that in general the contributions of pSNPs or pGenes were the strongest, followed by iGenes within pathways, and linkage between genes was the lowest. The number of pSNPs, pQTLs and iGenes among different traits (Supplementary Table 8) also indicated different genetic bases for correlations among traits. Correlations between HD and GNP, GNP, and PN can attribute to all three genetic bases, and correlation between PN and KGW is mainly ascribed to pSNPs or pGenes. Of course, the resolution of GWAS cannot distinguish pGenes from pQTL that contain more than two tightly linked genes and cannot validate iGenes, these need to be confirmed using additional mapping populations and molecular approaches.

### Implementation of genetic correlations in molecular breeding by marker assisted selection (MAS)

Disadvantageous correlations among agronomic traits are important constraints to maximizing the potential of each trait. Therefore, breaking or weakening correlations is one of the problems faced by breeders. Our results reveal the diverse genetic bases of phenotypic correlation in rice and different strategies for improving the related traits. It is a difficult task to develop varieties with short growth duration and more GNP due to the high proportion of pGenes, pQTLs and interactions in the same pathways, in which the genotype with short growth duration is accompanied by few GNP in majority of the cases. To overcome the contradictory effect of pGenes or iGenes, farmers and breeders tend to utilize genotypes with weak sensitivity to day-length so as to develop the varieties that adapt the long-day environment with the cost of moderately decreased GNP during the domestication of *japonica* (Xue et al., 2008). In pQTLs, genotypes with short growth duration and higher GNP were developed by breaking the linkage between genes controlling growth duration and genes controlling GNP (Figure 5D), except for the selection of genotypes with weak effect. This practice suggests a potential to overcome disadvantageous correlations between growth duration and GNP, that is, we can monitor the genotypic relationship between genes with tight linkage by molecular markers and thereby identify the rare advantageous genotypic combinations during breeding. It is undeniable that the positive correlation between growth duration and GNP is advantageous in environments where the light and temperature resources are adequate for the growth and harvest of *indica* varieties in low latitudes and altitudes (Khush, 1997; Londo et al., 2006). Our results indicated that the phenotypic correlations and their genetic bases (including pGenes, pQTLs and iGenes) vary in different environments and sometimesare environment-specific, and their utilization strategies during domestication and breeding as indicated above depends on the geographic location, especially the latitude and altitude. Our reported QTLs, pGenes, pQTLs and iGenes were clearly labeled by the locations where they detected (Supplementary Tables 4, 6, 7, 9, 10), this will facilitate their utilization in breeding and further study such as the molecular mechanism of GxE interaction.

In summary, phenotypic correlations among growth duration and yield traits can be advantageous or disadvantageous depending on variety types and environments. Selection for pGenes or iGenes needs to focus on the genotypes with weak effects when two traits show disadvantageous negative correlations or on genotypes with strong effect when two traits have advantageous positive correlations. Discovery of pQTLs attributed to different genes with strong LD or iGenes will provide breeders the opportunities to overcome the disadvantageous phenotypic correlations among traits by breaking the disadvantageous linkages or genotype combination for two genes, and also provide the opportunities to improve the breeding efficiency by simultaneously selecting two trait pairs with advantageous phenotypic correlation through MAS.

## Author contributions

FL and HZ designed the experiments. FL wrote the manuscript and performed data analysis. JX, XZ, XW, XM, ZhiZ, LZ, and SZ helped in data analysis. YZ, ZhaZ, and MR helped to prepare the manuscript. HZ, ZL, and JL conceived and supervised the project. HZ and ZL conceptualized and guided the study.

### Conflict of interest statement

The authors declare that the research was conducted in the absence of any commercial or financial relationships that could be construed as a potential conflict of interest.

## Abbreviations

3KRGP: 3000 Rice Genomes Project
CMLM: Compressed mixed linear model
CS: Changsha
GNP: Grain number per panicle
GWAS: Genome-wide association study
HD: Heading date
iGene: Interactive gene
KGW: Kilo-grain weight
LD: Linkage disequilibrium
MAS: Marker-assisted selection
MCC: Mini core collection
OD: Opposite direction effect
pGene: Pleiotropic gene
PN: Panicle number per plant
Pqtl: Pleiotropic QTL
pSNP: Pleiotropic SNP
PVC: Phenotypic variance contribution
QTL: Quantitative trait locus
SD: Same direction effect
SY: Sanya
SNP: Single nucleotide polymorphism.
