# Supplementary material for: Genetic Basis Underlying Correlations Among Growth Duration and Yield Traits Revealed by GWAS in Rice (Oryza sativa L.)
Source: Front Plant Sci. 2018 May 22;9:650. doi: 10.3389/fpls.2018.00650 (PMC5972282; doi:10.3389/fpls.2018.00650)
Supplement: Supplementary file 1 [file Table_1.DOCX]

**SUPPLEMENTARY TABLE 1 | Accessions in the mini core collection.**

| **ID (266)** | ***Indica / Japonica*** | **Variety name** | **LAN/**  **IMP/**  **IML** | **Country** | **Continent** | **Heading date** | **Paddy or Upland** | **Source of genotype** |
| --- | --- | --- | --- | --- | --- | --- | --- | --- |
| CH1305 | *Japonica* | Nipponbare | LAN | Japan | Asia | - | Paddy | Reference |
| CH1003 | *Japonica* | Heibiao | LAN | Korea | Asia | Early | Upland | 3KRGP |
| CH1004 | *Japonica* | Sansuijing | LAN | Japan | Asia | Intermediate | Paddy | 3KRGP |
| CH1005 | *Japonica* | Zaoshengbai | LAN | Japan | Asia | Early Intermediate | Paddy | 3KRGP |
| CH1008 | *Japonica* | Qiuguang Tengxi 104 | IMP | Japan | Asia | Intermediate | Paddy | 3KRGP |
| CH1009 | *Japonica* | Wanshi | LAN | Japan | Asia | Intermediate | Paddy | 3KRGP |
| CH1012 | *Indica* | Yikong | LAN | Vietnam | Asia | Late | Paddy | 3KRGP |
| CH1013 | *Indica* | Tianhan | LAN | Vietnam | Asia | Late | Paddy | 3KRGP |
| CH1014 | *Japonica* | Baxiang | LAN | Vietnam | Asia | Late | Paddy | 3KRGP |
| CH1015 | *Indica* | Yuenanzao | LAN | Vietnam | Asia | Early | Paddy | 3KRGP |
| CH1016 | *Indica* | Malaihong | LAN | Malaysia | Asia | Intermediate | Paddy | 3KRGP |
| CH1017 | *Indica* | - | IML | India | Asia | Intermediate | Paddy | 3KRGP |
| CH1018 | *Indica* | - | IML | India | Asia | Late | Paddy | 3KRGP |
| CH1019 | *Indica* | Sililanka 1 | IML | Sri Lanka | Asia | Intermediate | Paddy | 3KRGP |
| CH1021 | *Japonica* | Wuziluosi 215 | IML | Russia | Europe | Early | Paddy | 3KRGP |
| CH1022 | *Indica* | Kahamu | LAN | Romania | Europe | - | Paddy | 3KRGP |
| CH1023 | *Japonica* | Aomierte 168 | IML | Hungary | Europe | Early | Paddy | 3KRGP |
| CH1024 | *Japonica* | Aerjituo | LAN | Bulgaria | Europe | Early | Paddy | 3KRGP |
| CH1027 | *Japonica* | American Huangke | LAN | America | America | Intermediate | Paddy | 3KRGP |
| CH1028 | *Indica* | Buleida A-75 | IML | Mexico | America | Late | Paddy | 3KRGP |
| CH1029 | *Japonica* | Jineiya rice | LAN | Guinea | Africa | Early | Paddy | 3KRGP |
| CH1030 | *Indica* | - | IML | Australian | Oceania | Late | Paddy | 3KRGP |
| CH1032 | *Japonica* | Gaoliqiu | LAN | Korea | Asia | Late | Paddy | 3KRGP |
| CH1034 | *Indica* | Xianluosichi | LAN | Thailand | Asia | Intermediate | Paddy | 3KRGP |
| CH1035 | *Japonica* | - | LAN | Indonesia | Asia | Late | Paddy | 3KRGP |
| CH1037 | *Indica* | - | IMP | Indonesia | Asia | Late | Paddy | 3KRGP |
| CH1039 | *Indica* | - | IMP | Philippines | Asia | Late | Paddy | 3KRGP |
| CH1041 | *Indica* | - | IMP | IRRI | Asia | Early | Paddy | 3KRGP |
| CH1044 | *Indica* | - | LAN | India | Asia | Early | Paddy | 3KRGP |
| CH1045 | *Indica* | - | LAN | India | Asia | Late | Paddy | 3KRGP |
| CH1046 | *Indica* | - | LAN | India | Asia | Late | Paddy | 3KRGP |
| CH1048 | *Indica* | - | IMP | India | Asia | Late | Paddy | 3KRGP |
| CH1049 | *Indica* | - | IMP | Sri Lanka | Asia | Late | Paddy | 3KRGP |
| CH1054 | *Japonica* | Albania | LAN | Albania | Europe | Early | Paddy | 3KRGP |
| CH1056 | *Indica* | American rice | LAN | America | America | Intermediate | Paddy | 3KRGP |
| CH1059 | *Japonica* | - | LAN | Brazil | America | Intermediate | Paddy | 3KRGP |
| CH1060 | *Japonica* | - | LAN | Argentina | America | Intermediate | Paddy | 3KRGP |
| CH1061 | *Japonica* | - | LAN | Egypt | Africa | Late | Paddy | 3KRGP |
| CH1062 | *Indica* | - | IMP | Ivory Coast | Africa | Early | Paddy | 3KRGP |
| CH1063 | *Indica* | - | IML | Uganda | Africa | Intermediate | Paddy | 3KRGP |
| CH1067 | *Japonica* | - | IML | Australian | Oceania | Intermediate | Paddy | 3KRGP |
| CH1068 | *Indica* | Santiannuo | LAN | Japan | Asia | Late | Paddy | 3KRGP |
| CH1070 | *Japonica* | Gongchengxiang | LAN | Japan | Asia | Late | Paddy | 3KRGP |
| CH1071 | *Japonica* | Akitakomachi | IMP | Japan | Asia | Early | Paddy | 3KRGP |
| CH1072 | *Japonica* | Fuzhen 8 | IMP | Japan | Asia | Early | Paddy | 3KRGP |
| CH1076 | *Indica* | - | IMP | Indonesia | Asia | Late | Paddy | 3KRGP |
| CH1077 | *Indica* | Zachaodao 13 | IML | Nepal | Asia | Intermediate | Paddy | 3KRGP |
| CH1082 | *Indica* | - | IML | Egypt | Africa | Intermediate | Paddy | 3KRGP |
| CH1087 | *Indica* | - | IMP | Madagascar | Africa | Intermediate | Paddy | 3KRGP |
| CH1090 | *Japonica* | - | IML | Australian | Oceania | Intermediate | Paddy | 3KRGP |
| CH1091 | *Japonica* | - | IML | Australian | Oceania | Late | Paddy | 3KRGP |
| CH1092 | *Japonica* | - | IML | Australian | Oceania | Early | Paddy | 3KRGP |
| CH1097 | *Japonica* | Tieganwu | LAN | Zhejiang province of China | MCC | Late | Paddy | 3KRGP |
| CH1098 | *Japonica* | Xiushui 115 | IMP | Zhejiang province of China | MCC | Late | Paddy | 3KRGP |
| CH1099 | *Indica* | Erjiunan 1 | IMP | Zhejiang province of China | MCC | Early | Paddy | 3KRGP |
| CH1100 | *Indica* | Nanjing 11 | IMP | Jiangsu province of China | MCC | Intermediate | Paddy | 3KRGP |
| CH1101 | *Indica* | Aijiaoante | IMP | Guangdong province of China | MCC | Early | Paddy | 3KRGP |
| CH1102 | *Indica* | Gaungluai 4 | IMP | Guangdong province of China | MCC | Early | Paddy | 3KRGP |
| CH1103 | *Indica* | Nante | IMP | Jiangxi province of China | MCC | Early | Paddy | 3KRGP |
| CH1104 | *Indica* | Guichao 2 | IMP | Guangdong province of China | MCC | Early | Paddy | 3KRGP |
| CH1109 | *Indica* | Xiangzaoxian 7 | IMP | Hunan province of China | MCC | Intermediate | Paddy | 3KRGP |
| CH1110 | *Indica* | Huangsiguizhan | IMP | Guangdong province of China | MCC | Early | Paddy | 3KRGP |
| CH1113 | *Japonica* | Guangkexiangnuo | LAN | Guangxi province of China | MCC | Late | Paddy | 3KRGP |
| CH1116 | *Indica* | Wuningzipi | LAN | Hebei province of China | MCC | Early | Upland | 3KRGP |
| CH1118 | *Japonica* | Gaoyangdian rice | LAN | Hebei province of China | MCC | Intermediate | Paddy | 3KRGP |
| CH1119 | *Japonica* | Dandonglu rice | LAN | Liaoning province of China | MCC | Early | Upland | 3KRGP |
| CH1120 | *Japonica* | Laoguangtou 83 | LAN | Heilongjiang province of China | MCC | Early | Paddy | 3KRGP |
| CH1122 | *Japonica* | Muxiqiu | LAN | Shanghai city of China | MCC | Intermediate | Paddy | 3KRGP |
| CH1126 | *Indica* | Qiuqianbai | LAN | Anhui province of China | MCC | Intermediate | Paddy | 3KRGP |
| CH1128 | *Indica* | Jinxibai | LAN | Jiangxi province of China | MCC | Late | Paddy | 3KRGP |
| CH1129 | *Indica* | Taishannuo | LAN | Jiangxi province of China | MCC | Early | Paddy | 3KRGP |
| CH1131 | *Indica* | Jinbaoyin | LAN | Fujian province of China | MCC | Late | Paddy | 3KRGP |
| CH1132 | *Indica* | Minbeiwanxian | LAN | Fujian province of China | MCC | Late | Paddy | 3KRGP |
| CH1134 | *Japonica* | Yizhixiang | LAN | Fujian province of China | MCC | Late | Paddy | 3KRGP |
| CH1137 | *Indica* | Esiniu | LAN | Guangdong province of China | MCC | Late | Paddy | 3KRGP |
| CH1141 | *Indica* | Heidu 4 | LAN | Guangdong province of China | MCC | Early | Paddy | 3KRGP |
| CH1144 | *Indica* | Qiyuexian | LAN | Guangxi province of China | MCC | Late | Paddy | 3KRGP |
| CH1145 | *Indica* | Dongtingwanxian | LAN | Hubei province of China | MCC | Intermediate | Paddy | 3KRGP |
| CH1148 | *Japonica* | Bawangbian 1 | LAN | Hubei province of China | MCC | Intermediate | Paddy | 3KRGP |
| CH1149 | *Indica* | Xugunuo | LAN | Hunan province of China | MCC | Intermediate | Paddy | 3KRGP |
| CH1150 | *Japonica* | Muguanuo | LAN | Hunan province of China | MCC | Intermediate | Paddy | 3KRGP |
| CH1155 | *Indica* | Zhongnong 4 | LAN | Sichuan province of China | MCC | Intermediate | Paddy | 3KRGP |
| CH1158 | *Indica* | Three**Seventy | LAN | Yunnan province of China | MCC | Late | Upland | 3KRGP |
| CH1159 | *Indica* | Qitoubaigu | LAN | Yunnan province of China | MCC | Early | Paddy | 3KRGP |
| CH1161 | *Indica* | Zimi | LAN | Yunnan province of China | MCC | Intermediate | Upland | 3KRGP |
| CH1162 | *Indica* | Xiaohonggu | LAN | Yunnan province of China | MCC | Intermediate | Upland | 3KRGP |
| CH1164 | *Indica* | Gongju 73 | LAN | Yunnan province of China | MCC | Early | Upland | 3KRGP |
| CH1165 | *Indica* | Qitougu | LAN | Yunnan province of China | MCC | Late | Paddy | 3KRGP |
| CH1166 | *Indica* | Zinuo | LAN | Yunnan province of China | MCC | Intermediate | Paddy | 3KRGP |
| CH1167 | *Indica* | Muowanggunei | LAN | Yunnan province of China | MCC | Late | Upland | 3KRGP |
| CH1169 | *Indica* | Jinzhinuo | LAN | Yunnan province of China | MCC | Late | Paddy | 3KRGP |
| CH1170 | *Indica* | Jixuenuo | LAN | Yunnan province of China | MCC | Intermediate | Upland | 3KRGP |
| CH1179 | *Japonica* | Hongkezhenuo | LAN | Guizhou province of China | MCC | Intermediate | Paddy | 3KRGP |
| CH1183 | *Japonica* | Yangkenuo | LAN | Guizhou province of China | MCC | Intermediate | Paddy | 3KRGP |
| CH1184 | *Japonica* | Maguzi | LAN | Shanxi province of China | MCC | Early | Paddy | 3KRGP |
| CH1185 | *Japonica* | Laohong rice | LAN | Shanxi province of China | MCC | Intermediate | Paddy | 3KRGP |
| CH1186 | *Indica* | Jabala | LAN | Xizang province of China | MCC | Early | Paddy | 3KRGP |
| CH1190 | *Indica* | Baoxuan 21 | IMP | Guangdong province of China | MCC | Late | Paddy | 3KRGP |
| CH1191 | *Indica* | Wenxiangnuo | LAN | Yunnan province of China | MCC | Late | Paddy | 3KRGP |
| CH1193 | *Indica* | Dawannuo | LAN | Yunnan province of China | MCC | Late | Paddy | 3KRGP |
| CH1194 | *Indica* | Xianggu | LAN | Yunnan province of China | MCC | Intermediate | Upland | 3KRGP |
| CH1196 | *Japonica* | Lengshuigu 2 | LAN | Yunnan province of China | MCC | Late | Paddy | 3KRGP |
| CH1198 | *Japonica* | Huangpinuo | LAN | Yunnan province of China | MCC | Late | Paddy | 3KRGP |
| CH1202 | *Japonica* | Zimangfeie | LAN | Guizhou province of China | MCC | Late | Upland | 3KRGP |
| CH1204 | *Indica* | Liusha 1 | IMP | Guangxi province of China | MCC | Early | Paddy | 3KRGP |
| CH1205 | *Indica* | Binwan 3 | IMP | Hunan province of China | MCC | Late | Paddy | 3KRGP |
| CH1208 | *Indica* | Chengduai 3 | IMP | Sichuan province of China | MCC | Intermediate | Paddy | 3KRGP |
| CH1209 | *Indica* | Aimakang | IMP | Sichuan province of China | MCC | Intermediate | Paddy | 3KRGP |
| CH1210 | *Indica* | Shufeng 101 | IMP | Sichuan province of China | MCC | Intermediate | Paddy | 3KRGP |
| CH1211 | *Japonica* | Lixinggeng | IMP | Sichuan province of China | MCC | Late | Paddy | 3KRGP |
| CH1213 | *Indica* | Ergangai | IMP | Guangdong province of China | MCC | Late | Paddy | 3KRGP |
| CH1214 | *Indica* | Guangluai 15 | IMP | Guangxi province of China | MCC | Early | Paddy | 3KRGP |
| CH1215 | *Indica* | Hongwan 1 | IMP | Fujian province of China | MCC | Late | Paddy | 3KRGP |
| CH1219 | *Indica* | Huke 3 | IMP | Sichuan province of China | MCC | Intermediate | Paddy | 3KRGP |
| CH1223 | *Japonica* | Liaogeng 287 | IMP | Liaoning province of China | MCC | Late | Paddy | 3KRGP |
| CH1224 | *Indica* | Zaoshuxianghei | IMP | Guangxi province of China | MCC | Early | Paddy | 3KRGP |
| CH1226 | *Japonica* | Geng 87-304 | IMP | Hunan province of China | MCC | Late | Paddy | 3KRGP |
| CH1227 | *Indica* | Xiangwanxian 3 | IMP | Hunan province of China | MCC | Late | Paddy | 3KRGP |
| CH1229 | *Indica* | Zaoxian 240 | IMP | Anhui province of China | MCC | Early | Paddy | 3KRGP |
| CH1230 | *Indica* | Dangyu 5 | IMP | Anhui province of China | MCC | Late | Paddy | 3KRGP |
| CH1232 | *Indica* | Sibeitichao 6 | IMP | Beijing city of China | MCC | Intermediate | Paddy | 3KRGP |
| CH1233 | *Indica* | Hongainuo | LAN | Guangxi province of China | MCC | Late | Paddy | 3KRGP |
| CH1234 | *Indica* | Wanlixian | LAN | Hunan province of China | MCC | Intermediate | Paddy | 3KRGP |
| CH1235 | *Indica* | Aizaizhan | LAN | Guangxi province of China | MCC | Late | Paddy | 3KRGP |
| CH1236 | *Indica* | Xiaobaimi | LAN | Guizhou province of China | MCC | Intermediate | Paddy | 3KRGP |
| CH1237 | *Indica* | Yanshuichi | LAN | Fujian province of China | MCC | Late | Paddy | 3KRGP |
| CH1238 | *Japonica* | Xishen 15 | LAN | Guangdong province of China | MCC | Late | Upland | 3KRGP |
| CH1239 | *Japonica* | Honggenghangu | LAN | Guangxi province of China | MCC | Late | Upland | 3KRGP |
| CH1241 | *Japonica* | Yuyannuo | LAN | Yunnan province of China | MCC | Late | Paddy | 3KRGP |
| CH1242 | *Indica* | 80B | IMP | Hunan province of China | MCC | Early | Paddy | 3KRGP |
| CH1243 | *Indica* | Gu 154 | IMP | Hunan province of China | MCC | Intermediate | Paddy | 3KRGP |
| CH1245 | *Indica* | IR 661-1 | IMP | Hunan province of China | MCC | Intermediate | Paddy | 3KRGP |
| CH1246 | *Indica* | Pei C122 | IMP | Hunan province of China | MCC | Intermediate | Paddy | 3KRGP |
| CH1247 | *Japonica* | Geng 7623 | IMP | Shanghai city of China | MCC | Early | Paddy | 3KRGP |
| CH1248 | *Japonica* | Ninghui 21 | IMP | Jiangsu province of China | MCC | Intermediate | Paddy | 3KRGP |
| CH1249 | *Japonica* | 76-1 | IMP | Liaoning province of China | MCC | Intermediate | Paddy | 3KRGP |
| CH1250 | *Japonica* | Huhui 628 | IMP | Hunan province of China | MCC | Intermediate | Paddy | 3KRGP |
| CH1251 | *Indica* | Teqingxuanhui | IMP | Hunan province of China | MCC | Intermediate | Paddy | 3KRGP |
| CH1253 | *Indica* | JWR 221 | IMP | Jiangsu province of China | MCC | Intermediate | Paddy | 3KRGP |
| CH1255 | *Indica* | Baikehanhe | LAN | Hunan province of China | MCC | - | - | 3KRGP |
| CH1259 | *Japonica* | Lengshuinuo | LAN | Yunnan province of China | MCC | Late | Paddy | 3KRGP |
| CH1260 | *Indica* | Haoxiang | LAN | Yunnan province of China | MCC | Late | Paddy | 3KRGP |
| CH1265 | *Indica* | L301B | IMP | Hunan province of China | MCC | Early | Paddy | 3KRGP |
| CH1267 | *Indica* | Jinnante 43B | IMP | Hunan province of China | MCC | Early | Paddy | 3KRGP |
| CH1268 | *Japonica* | Zaoshunonghu 6 | IMP | Hunan province of China | MCC | Late | Paddy | 3KRGP |
| CH1269 | *Indica* | Qingsiai 16B | IMP | Guangdong province of China | MCC | Intermediate | Paddy | 3KRGP |
| CH1274 | *Japonica* | Liming B | IMP | Liaoning province of China | MCC | Intermediate | Paddy | 3KRGP |
| CH1276 | *Indica* | Baoxie-7B | IMP | Hunan province of China | MCC | Early | Paddy | 3KRGP |
| CH1277 | *Indica* | Gzhenshan 97B | IMP | Sichuan province of China | MCC | Intermediate | Paddy | 3KRGP |
| CH1278 | *Indica* | 88B | IMP | Jiangsu province of China | MCC | Intermediate | Paddy | 3KRGP |
| CH1283 | *Japonica* | Taizhong 65 | LAN | Taiwan province of China | MCC | Early | Paddy | 3KRGP |
| CH1284 | *Indica* | Taichung Native 1 | LAN | Taiwan province of China | MCC | Early | Paddy | 3KRGP |
| CH1288 | *Japonica* | Shuiyuan 300 grain | LAN | Hebei province of China | MCC | Intermediate | Paddy | 3KRGP |
| CH1289 | *Japonica* | Yelicanghua | LAN | Hebei province of China | MCC | Intermediate | Paddy | 3KRGP |
| CH1291 | *Japonica* | Baige rice | LAN | Jiangsu province of China | MCC | Late | Paddy | 3KRGP |
| CH1293 | *Indica* | Liushizao | LAN | Anhui province of China | MCC | Early | Paddy | 3KRGP |
| CH1297 | *Indica* | Qingke | LAN | Yunnan province of China | MCC | Intermediate | Upland | 3KRGP |
| CH1298 | *Indica* | Haohuangla | LAN | Yunnan province of China | MCC | - | Paddy | 3KRGP |
| CH1001 | *Japonica* | Jianghua rice | LAN | Korea | Asia | Early | Paddy | 3KRGP |
| CH1002 | *Japonica* | Qingjingzaosheng | LAN | Korea | Asia | Early | Paddy | 3KRGP |
| CH1006 | *Japonica* | Zhuyuan | LAN | Japan | Asia | Early | Paddy | 3KRGP |
| CH1010 | *Japonica* | Ailuyu | LAN | Japan | Asia | Early Intermediate | Paddy | 3KRGP |
| CH1020 | *Japonica* | Red 90 | IML | Russia | Europe | Early | Paddy | 3KRGP |
| CH1026 | *Japonica* | Linguo | LAN | Italian | Europe | Early | Paddy | 3KRGP |
| CH1038 | *Indica* | - | LAN | Indonesia | Asia | Late | Paddy | 3KRGP |
| CH1050 | *Indica* | - | IMP | Bangladesh | Asia | Late | Paddy | 3KRGP |
| CH1055 | *Japonica* | Keluoduo B | IML | France | Europe | Early | Paddy | 3KRGP |
| CH1057 | *Indica* | - | IML | Columbia | America | Intermediate | Paddy | 3KRGP |
| CH1064 | *Indica* | - | IML | Australian | Oceania | Intermediate | Paddy | 3KRGP |
| CH1069 | *Japonica* | Chimao | LAN | Japan | Asia | Early | Paddy | 3KRGP |
| CH1073 | *Japonica* | Qinnuo | LAN | Japan | Asia | Intermediate | Upland | 3KRGP |
| CH1074 | *Indica* | - | IML | Vietnam | Asia | Intermediate | Paddy | 3KRGP |
| CH1075 | *Indica* | - | IML | Laos | Asia | Late | Paddy | 3KRGP |
| CH1081 | *Indica* | - | IMP | India | Asia | Late | Paddy | 3KRGP |
| CH1083 | *Japonica* | - | IMP | Ivory Coast | Africa | Intermediate | Paddy | 3KRGP |
| CH1085 | *Japonica* | - | IMP | Ivory Coast | Africa | Late | Paddy | 3KRGP |
| CH1086 | *Japonica* | - | IMP | Nigeria | Africa | Intermediate | Paddy | 3KRGP |
| CH1088 | *Japonica* | - | IML | Australian | Oceania | Intermediate | Paddy | 3KRGP |
| CH1093 | *Japonica* | - | IML | America | America | Intermediate | Paddy | 3KRGP |
| CH1094 | *Indica* | - | IML | Cuba | America | Late | Paddy | 3KRGP |
| CH1095 | *Indica* | - | IMP | Cuba | America | Intermediate | Paddy | 3KRGP |
| CH1096 | *Indica* | - | IML | India | Asia | Late | Paddy | 3KRGP |
| CH1105 | *Japonica* | Taidonglu rice | LAN | Taiwan province of China | MCC | Early | Upland | 3KRGP |
| CH1106 | *Indica* | Taizhongxianxuan 2 | LAN | Taiwan province of China | MCC | Early | Paddy | 3KRGP |
| CH1107 | *Indica* | Jiefangxian | LAN | Jiangxi province of China | MCC | Early | Paddy | 3KRGP |
| CH1108 | *Japonica* | Hongmisandan | LAN | Jiangxi province of China | MCC | Late | Paddy | 3KRGP |
| CH1111 | *Indica* | Jinyou 1 | IMP | Fujian province of China | MCC | Intermediate | Paddy | 3KRGP |
| CH1112 | *Indica* | Chengnongshuijin | IMP | Sichuan province of China | MCC | Intermediate | Paddy | 3KRGP |
| CH1114 | *Indica* | Baoxie 123B | IMP | Hunan province of China | MCC | Early | Paddy | 3KRGP |
| CH1115 | *Indica* | Piwusheng | LAN | Yunnan province of China | MCC | Late | Paddy | 3KRGP |
| CH1117 | *Japonica* | Longhuamaohu | LAN | Hebei province of China | MCC | Early | Paddy | 3KRGP |
| CH1125 | *Japonica* | Cunsanli | LAN | Jiangsu province of China | MCC | Intermediate | Upland | 3KRGP |
| CH1130 | *Indica* | Aihechi | LAN | Jiangxi province of China | MCC | Intermediate | Paddy | 3KRGP |
| CH1133 | *Indica* | Lucaihao | LAN | Fujian province of China | MCC | Early | Paddy | 3KRGP |
| CH1139 | *Indica* | Nanxiongzaoyou | LAN | Guangdong province of China | MCC | Early | Paddy | 3KRGP |
| CH1142 | *Japonica* | Chikenuo | LAN | Guangdong province of China | MCC | Early | Paddy | 3KRGP |
| CH1146 | *Indica* | Liuyezhan | LAN | Hubei province of China | MCC | Intermediate | Paddy | 3KRGP |
| CH1147 | *Indica* | Xuanenchangtan | LAN | Hubei province of China | MCC | Intermediate | Paddy | 3KRGP |
| CH1151 | *Japonica* | Hongqi 5 | LAN | Hunan province of China | MCC | Late | Paddy | 3KRGP |
| CH1152 | *Indica* | Zaoma rice | LAN | Henan province of China | MCC | Intermediate | Upland | 3KRGP |
| CH1156 | *Indica* | Honggu | LAN | Sichuan province of China | MCC | Intermediate | Paddy | 3KRGP |
| CH1163 | *Japonica* | Wuzidui | LAN | Yunnan province of China | MCC | Late | Paddy | 3KRGP |
| CH1168 | *Indica* | Haolai | LAN | Yunnan province of China | MCC | Late | Paddy | 3KRGP |
| CH1171 | *Indica* | Fanhaopi | LAN | Yunnan province of China | MCC | Intermediate | Upland | 3KRGP |
| CH1173 | *Indica* | Beizinuo | LAN | Yunnan province of China | MCC | Late | Paddy | 3KRGP |
| CH1180 | *Japonica* | Cungunuo | LAN | Guizhou province of China | MCC | Intermediate | Paddy | 3KRGP |
| CH1181 | *Indica* | Youzhan | LAN | Guizhou province of China | MCC | Late | Upland | 3KRGP |
| CH1182 | *Japonica* | Guantuibaihe 1 | LAN | Guizhou province of China | MCC | Late | Paddy | 3KRGP |
| CH1187 | *Japonica* | Heimang rice | LAN | Ningxia province of China | MCC | Early | Paddy | 3KRGP |
| CH1189 | *Indica* | Menjiagao 1 | LAN | Hainan province of China | MCC | Late | Upland | 3KRGP |
| CH1195 | *Japonica* | Haobayong 1 | LAN | Yunnan province of China | MCC | Intermediate | Upland | 3KRGP |
| CH1197 | *Indica* | Menjiading 2 | LAN | Hainan province of China | MCC | Late | Upland | 3KRGP |
| CH1199 | *Japonica* | Banjiemang | LAN | Yunnan province of China | MCC | Intermediate | Paddy | 3KRGP |
| CH1203 | *Indica* | Dongtinwanxian | LAN | Hubei province of China | MCC | Intermediate | Paddy | 3KRGP |
| CH1216 | *Indica* | Xiangaizao 10 | IMP | Hunan province of China | MCC | Early | Paddy | 3KRGP |
| CH1217 | *Indica* | Xiangwanxian 1 | IMP | Hunan province of China | MCC | Late | Paddy | 3KRGP |
| CH1220 | *Indica* | Aituogu 151 | IMP | Sichuan province of China | MCC | Intermediate | Paddy | 3KRGP |
| CH1221 | *Japonica* | Zhonghua 8 | IMP | Beijing city of China | MCC | Intermediate | Paddy | 3KRGP |
| CH1222 | *Japonica* | Jindao 1 | IMP | Shanxi province of China | MCC | Intermediate | Paddy | 3KRGP |
| CH1225 | *Indica* | Momi | IMP | Guangxi province of China | MCC | Late | Paddy | 3KRGP |
| CH1228 | *Indica* | Zhenxian 232 | IMP | Jiangsu province of China | MCC | Intermediate | Paddy | 3KRGP |
| CH1231 | *Japonica* | Zhendao 5 | IMP | Henan province of China | MCC | Intermediate | Paddy | 3KRGP |
| CH1240 | *Japonica* | Lamujia | LAN | Yunnan province of China | MCC | Late | Upland | 3KRGP |
| CH1244 | *Indica* | Gui 630 | IMP | Hunan province of China | MCC | Intermediate | Paddy | 3KRGP |
| CH1252 | *Indica* | Xianghui 91269 | IMP | Hunan province of China | MCC | Intermediate | Paddy | 3KRGP |
| CH1256 | *Indica* | Scented rice | LAN | Henan province of China | MCC | Early | Paddy | 3KRGP |
| CH1257 | *Japonica* | Shanjiugu | LAN | Sichuan province of China | MCC | Intermediate | Upland | 3KRGP |
| CH1258 | *Indica* | Laozaogu | LAN | Yunnan province of China | MCC | - | Upland | 3KRGP |
| CH1262 | *Indica* | Jinnante B | IMP | Hunan province of China | MCC | Early | Paddy | 3KRGP |
| CH1263 | *Indica* | Zhuzhen B | IMP | Hunan province of China | MCC | Early | Paddy | 3KRGP |
| CH1264 | *Indica* | Chaoyangyihao B | IMP | Hunan province of China | MCC | Early | Paddy | 3KRGP |
| CH1266 | *Japonica* | Annongwangeng B | IMP | Hunan province of China | MCC | Late | Paddy | 3KRGP |
| CH1271 | *Indica* | Xiangai B | IMP | Jiangxi province of China | MCC | Late | Paddy | 3KRGP |
| CH1272 | *Indica* | Jiangnongzao 1 | IMP | Jiangxi province of China | MCC | Early | Paddy | 3KRGP |
| CH1273 | *Indica* | Jinghu B | IMP | Anhui province of China | MCC | Intermediate | Paddy | 3KRGP |
| CH1275 | *Indica* | Dianrui 409B | IMP | Yunnan province of China | MCC | Intermediate | Paddy | 3KRGP |
| CH1281 | *Japonica* | Xingguo | LAN | Jilin province of China | MCC | Early | Paddy | 3KRGP |
| CH1282 | *Indica* | Leihuozhan | LAN | Anhui province of China | MCC | Early | Paddy | 3KRGP |
| CH1285 | *Indica* | Mamagu | LAN | Sichuan province of China | MCC | Intermediate | Paddy | 3KRGP |
| CH1286 | *Indica* | Meihuanuo | LAN | Sichuan province of China | MCC | Intermediate | Paddy | 3KRGP |
| CH1290 | *Japonica* | Weiguo | LAN | Liaoning province of China | MCC | Intermediate | Paddy | 3KRGP |
| CH1294 | *Indica* | Sanbaili | LAN | Jiangxi province of China | MCC | Early | Paddy | 3KRGP |
| CH1295 | *Indica* | Wukezhan | LAN | Fujian province of China | MCC | Late | Paddy | 3KRGP |
| CH1296 | *Japonica* | Haomake (K) | LAN | Yunnan province of China | MCC | - | Upland | 3KRGP |
| CH1299 | *Indica* | Nangaogu | LAN | Yunnan province of China | MCC | - | Paddy | 3KRGP |
| CH1302 | *Japonica* | Yuefu | LAN | Japan | Asia | Intermediate | Upland | 3KRGP |
| CH1058 | *Japonica* | - | IMP | Brazil | America | Intermediate | Upland | 3KRGP |
| CH1084 | *Japonica* | - | IMP | Ivory Coast | Africa | Intermediate | Upland | 3KRGP |
| CH1192 | *Japonica* | Haobuka | LAN | Yunnan province of China | MCC | Late | Upland | 3KRGP |
| CH1033 | *Japonica* | - | LAN | Laos | Asia | Late | Paddy | 3KRGP |
| CH1178 | *Indica* | Maweizhan | LAN | Guizhou province of China | MCC | Early | Paddy | 3KRGP |
| CH1011 | *Indica* | Aiyisi | LAN | Vietnam | Asia | Late | Paddy | 3KRGP |
| CH1052 | *Japonica* | - | - | Pakistan | Asia | Intermediate | Paddy | 3KRGP |
| CH1136 | *Indica* | Simiao | LAN | Guangdong province of China | MCC | Late | Paddy | 3KRGP |
| CH1154 | *Japonica* | Nantiangangjiu | LAN | Sichuan province of China | MCC | Intermediate | Paddy | 3KRGP |
| CH1089 | *Japonica* | - | IML | Australian | Oceania | Intermediate | Paddy | 3KRGP |
| CH1300 | *Japonica* | Ximaxian | LAN | Yunnan province of China | MCC | Late | Paddy | 3KRGP |
| CH1301 | *Japonica* | IRAT109 | IMP | Philippines | Asia | - | Paddy | 3KRGP |
| CH1303 | *Japonica* | Haogelao | IMP | Yunnan province of China | MCC | - | Upland | 3KRGP |
| CH1160 | *Japonica* | Benbanggu | LAN | Yunnan province of China | MCC | Intermediate | Upland | 3KRGP |
| CH1047 | *Indica* | - | IMP | India | Asia | Early | Paddy | 3KRGP |
| CH1157 | *Indica* | Sankecun | LAN | Sichuan province of China | MCC | Intermediate | Paddy | 3KRGP |
| CH1025 | *Japonica* | - | LAN | - | Europe | Late | Paddy | 3KRGP |
| CH1121 | *Japonica* | Baimao rice | LAN | Heilongjiang province of China | MCC | Early | Upland | 3KRGP |
| CH1201 | *Japonica* | Feienuo 2 | LAN | Guizhou province of China | MCC | Late | Upland | 3KRGP |
| CH1079 | *Indica* | - | IML | India | Asia | Late | Paddy | 3KRGP |

"-": the missing information in accessions; MCC: mini core collection; 3KRGP: 3000 rice genomes project.
