# Supplementary material for: Genetic Basis Underlying Correlations Among Growth Duration and Yield Traits Revealed by GWAS in Rice (Oryza sativa L.)
Source: Front Plant Sci. 2018 May 22;9:650. doi: 10.3389/fpls.2018.00650 (PMC5972282; doi:10.3389/fpls.2018.00650)
Supplement: Supplementary file 2 [file Table_2.DOCX]

**SUPPLEMENTARY TABLE 2 | Phenotypic variation for four agronomic traits at Sanya and Changsha.**

|  | **SY** | | | | | | **CS** | | | | | |
| --- | --- | --- | --- | --- | --- | --- | --- | --- | --- | --- | --- | --- |
|  | **Mean** | | | **Min** | **Max** | **CV** | **Mean** | | | **Min** | **Max** | **CV** |
|  | **Full** | ***Indica*** | ***Japonica*** |  |  |  | **Full** | ***Indica*** | ***japonica*** |  |  |  |
| HD | 85.57±13.36 | 76.10±14.06 | 79.85±13.38 | 60.00 | 116.00 | 0.156 | 87.62±12.13 | 90.81±20.75 | 92.32±23.45 | 73.00 | 117.00 | 0.138 |
| GNP | 163.86±59.17 | 147.49±62.50 | 176.72±60.00 | 48.75 | 336.00 | 0.361 | 184.14±51.66 | 187.57±56.43 | 184.38±54.87 | 63.83 | 319.30 | 0.281 |
| PN | 10.65±3.87 | 9.16±3.79 | 11.60±3.64 | 3.00 | 24.30 | 0.364 | 14.20±4.14 | 12.92±4.27 | 15.21±4.05 | 5.30 | 25.00 | 0.292 |
| KGW | 24.94±3.47 | 25.82±3.75 | 24.53±3.43 | 14.55 | 33.97 | 0.139 | 20.86±2.65 | 21.60±3.27 | 20.47±2.33 | 13.27 | 30.81 | 0.127 |

HD: heading date; GNP: grain number per plant; PN: panicle number; KGW: kilo-grain weight; SY: Sanya; CS: Changsha; Min: minimum; Max: maximum; CV: coefficient of variation.
