# Supplementary material for: Genetic Basis Underlying Correlations Among Growth Duration and Yield Traits Revealed by GWAS in Rice (Oryza sativa L.)
Source: Front Plant Sci. 2018 May 22;9:650. doi: 10.3389/fpls.2018.00650 (PMC5972282; doi:10.3389/fpls.2018.00650)
Supplement: Supplementary file 3 [file Table_3.DOCX]

**SUPPLEMENTARY TABLE 3 | Correlations among four traits in different environments and populations.**

|  |  |  | **HD** | **GNP** | **PN** | **KGW** |
| --- | --- | --- | --- | --- | --- | --- |
| **SY** | ***Indica*** | HD | 1 |  |  |  |
|  |  | GNP | 0.635** | 1 |  |  |
|  |  | PN | -0.332** | -0.423** | 1 |  |
|  |  | KGW | -0.237** | -0.122 | -0.221** | 1 |
|  | ***Japonica*** | HD | 1 |  |  |  |
|  |  | GNP | 0.580** | 1 |  |  |
|  |  | PN | -0.306** | -0.414** | 1 |  |
|  |  | KGW | -0.347** | -0.420** | 0.048 | 1 |
| **CS** | ***Indica*** | HD | 1 |  |  |  |
|  |  | GNP | 0.443** | 1 |  |  |
|  |  | PN | -0.106 | -0.235** | 1 |  |
|  |  | KGW | -0.268** | -0.026 | -0.096 | 1 |
|  | ***Japonica*** | HD | 1 |  |  |  |
|  |  | GNP | 0.420** | 1 |  |  |
|  |  | PN | -0.435** | -0.365** | 1 |  |
|  |  | KGW | -0.139 | -0.240* | 0.054 | 1 |

"*": significant correlation at *P* = 0.05; "**": significant correlation at the *P* = 0.01 level; SY: Sanya; CS: Changsha; HD: heading date; GNP: grain number per plant; PN: panicle number; KGW: kilo-grain weight.
