# Supplementary material for: Genetic Basis Underlying Correlations Among Growth Duration and Yield Traits Revealed by GWAS in Rice (Oryza sativa L.)
Source: Front Plant Sci. 2018 May 22;9:650. doi: 10.3389/fpls.2018.00650 (PMC5972282; doi:10.3389/fpls.2018.00650)
Supplement: Supplementary file 5 [file Table_5.DOCX]

**SUPPLEMENTARY TABLE 5 | Numbers of QTLs for different traits identified by the genome-wide association study in different populations and environments.**

|  | **SY** | | | **CS** | | |
| --- | --- | --- | --- | --- | --- | --- |
|  | **Full** | ***Indica*** | ***Japonica*** | **Full** | ***Indica*** | ***Japonica*** |
| HD | 24 | 25 | 11 | 18 | 28 | 5 |
| GNP | 33 | 14 | 20 | 22 | 10 | 7 |
| PN | 34 | 17 | 14 | 9 | 9 | 14 |
| KGW | 28 | 18 | 11 | 27 | 17 | 11 |

SY: Sanya; CS: Changsha; HD: heading date; GNP: grain number per plant; PN: panicle number; KGW: kilo-grain weight.
