# Supplementary material for: Genetic Basis Underlying Correlations Among Growth Duration and Yield Traits Revealed by GWAS in Rice (Oryza sativa L.)
Source: Front Plant Sci. 2018 May 22;9:650. doi: 10.3389/fpls.2018.00650 (PMC5972282; doi:10.3389/fpls.2018.00650)
Supplement: Supplementary file 6 [file Table_6.DOCX]

**SUPPLEMENTARY TABLE 6 | Frequency of each bin covered by reported QTLs and pleiotropic QTLs that were mapped using a common SSR marker.**

| **Position** | | | **Frequency covered by reported QTLs** | | | | **pQTLs between different traits in previous studies** | | | | | |
| --- | --- | --- | --- | --- | --- | --- | --- | --- | --- | --- | --- | --- |
|  |  |  | **HD** | **GNP** | **PN** | **KGW** | **HD GNP** | **HD PN** | **HD KGW** | **GNP PN** | **GNP KGW** | **PN KGW** |
| 1 | 0 | 500000 | 0 | 0 | 0 | 0 |  |  |  |  |  |  |
| 1 | 500000 | 1500000 | 1 | 2 | 0 | 2 | pQTL1 |  | pQTL1 |  | pQTL1 |  |
| 1 | 1500000 | 2500000 | 1 | 2 | 0 | 4 | pQTL1 |  | pQTL1 |  | pQTL1 |  |
| 1 | 2500000 | 3500000 | 1 | 4 | 0 | 3 | pQTL1 |  | pQTL1 |  | pQTL1 |  |
| 1 | 3500000 | 4500000 | 1 | 5 | 0 | 2 | pQTL1 |  | pQTL1 |  | pQTL1 |  |
| 1 | 4500000 | 5500000 | 2 | 9 | 3 | 5 | pQTL1 | pQTL2 | pQTL1 | pQTL2 | pQTL1 | pQTL2 |
| 1 | 5500000 | 6500000 | 2 | 7 | 1 | 5 | pQTL1 |  |  | pQTL2 | pQTL1 | pQTL2 |
| 1 | 6500000 | 7500000 | 1 | 3 | 2 | 3 | pQTL1 |  |  | pQTL2 | pQTL1 | pQTL2 |
| 1 | 7500000 | 8500000 | 0 | 3 | 2 | 2 |  |  |  | pQTL2 | pQTL1 | pQTL2 |
| 1 | 8500000 | 9500000 | 0 | 1 | 1 | 0 |  |  |  | pQTL2 |  |  |
| 1 | 9500000 | 10500000 | 1 | 1 | 0 | 0 | pQTL2 |  |  |  |  |  |
| 1 | 10500000 | 11500000 | 3 | 0 | 1 | 1 |  |  |  |  |  |  |
| 1 | 11500000 | 12500000 | 2 | 1 | 1 | 0 | pQTL3 |  |  |  |  |  |
| 1 | 12500000 | 13500000 | 1 | 0 | 1 | 0 |  |  |  |  |  |  |
| 1 | 13500000 | 14500000 | 0 | 0 | 1 | 0 |  |  |  |  |  |  |
| 1 | 14500000 | 15500000 | 1 | 0 | 1 | 1 |  |  |  |  |  |  |
| 1 | 20500000 | 21500000 | 0 | 0 | 0 | 0 |  |  |  |  |  |  |
| 1 | 21500000 | 22500000 | 0 | 0 | 1 | 1 |  |  |  |  |  | pQTL3 |
| 1 | 22500000 | 23500000 | 0 | 0 | 1 | 0 |  |  |  |  |  |  |
| 1 | 23500000 | 24500000 | 0 | 0 | 1 | 0 |  |  |  |  |  |  |
| 1 | 24500000 | 25500000 | 0 | 0 | 1 | 2 |  |  |  |  |  | pQTL4 |
| 1 | 25500000 | 26500000 | 0 | 0 | 0 | 1 |  |  |  |  |  |  |
| 1 | 26500000 | 27500000 | 1 | 1 | 0 | 1 | pQTL4 |  | pQTL2 |  | pQTL2 |  |
| 1 | 27500000 | 28500000 | 2 | 0 | 2 | 0 |  | pQTL3 |  |  |  |  |
| 1 | 28500000 | 29500000 | 1 | 1 | 2 | 1 | pQTL5 | pQTL3 | pQTL3 |  | pQTL3 |  |
| 1 | 29500000 | 30500000 | 1 | 0 | 0 | 0 |  |  |  |  |  |  |
| 1 | 30500000 | 31500000 | 0 | 0 | 0 | 0 |  |  |  |  |  |  |
| 1 | 31500000 | 32500000 | 1 | 0 | 2 | 2 |  | pQTL4 | pQTL4 |  |  | pQTL5 |
| 1 | 32500000 | 33500000 | 2 | 0 | 1 | 3 |  | pQTL4 | pQTL4 |  |  | pQTL5 |
| 1 | 33500000 | 34500000 | 0 | 0 | 1 | 2 |  |  |  |  |  | pQTL5 |
| 1 | 34500000 | 35500000 | 0 | 1 | 3 | 1 |  |  |  |  | pQTL4 | pQTL5 |
| 1 | 35500000 | 36500000 | 0 | 2 | 0 | 0 |  |  |  |  |  |  |
| 1 | 36500000 | 37500000 | 0 | 3 | 0 | 0 |  |  |  |  |  |  |
| 1 | 37500000 | 38500000 | 0 | 5 | 0 | 0 |  |  |  |  |  |  |
| 1 | 38500000 | 39500000 | 1 | 1 | 0 | 0 |  |  |  |  |  |  |
| 1 | 39500000 | 40500000 | 0 | 1 | 0 | 0 |  |  |  |  |  |  |
| 1 | 40500000 | 41500000 | 2 | 2 | 1 | 2 | pQTL6 | pQTL5 | pQTL5 |  | pQTL5 |  |
| 1 | 41500000 | 42500000 | 3 | 0 | 0 | 1 |  |  | pQTL5 |  |  |  |
| 1 | 42500000 | 43500000 | 1 | 0 | 0 | 1 |  |  |  |  |  |  |
| 2 | 0 | 500000 | 0 | 0 | 0 | 0 |  |  |  |  |  |  |
| 2 | 500000 | 1500000 | 2 | 0 | 0 | 3 |  |  | pQTL6 |  |  |  |
| 2 | 1500000 | 2500000 | 2 | 0 | 0 | 1 |  |  | pQTL6 |  |  |  |
| 2 | 2500000 | 3500000 | 0 | 1 | 0 | 3 |  |  |  |  | pQTL6 |  |
| 2 | 3500000 | 4500000 | 1 | 1 | 0 | 2 |  |  | pQTL7 |  | pQTL6 |  |
| 2 | 4500000 | 5500000 | 0 | 1 | 0 | 1 |  |  |  |  | pQTL6 |  |
| 2 | 5500000 | 6500000 | 1 | 1 | 0 | 1 |  |  |  |  | pQTL6 |  |
| 2 | 6500000 | 7500000 | 0 | 1 | 0 | 2 |  |  |  |  | pQTL6 |  |
| 2 | 7500000 | 8500000 | 0 | 1 | 0 | 3 |  |  |  |  | pQTL6 |  |
| 2 | 8500000 | 9500000 | 0 | 1 | 0 | 2 |  |  |  |  | pQTL6 |  |
| 2 | 9500000 | 10500000 | 0 | 1 | 0 | 2 |  |  |  |  | pQTL6 |  |
| 2 | 10500000 | 11500000 | 2 | 3 | 2 | 3 | pQTL7 | pQTL6 | pQTL8 | pQTL3 | pQTL6 | pQTL6 |
| 2 | 16500000 | 17500000 | 1 | 0 | 0 | 1 |  |  |  |  |  |  |
| 2 | 17500000 | 18500000 | 1 | 0 | 0 | 0 |  |  |  |  |  |  |
| 2 | 18500000 | 19500000 | 3 | 1 | 0 | 0 | pQTL8 |  |  |  |  |  |
| 2 | 19500000 | 20500000 | 4 | 1 | 0 | 1 | pQTL8 |  | pQTL9 |  | pQTL7 |  |
| 2 | 20500000 | 21500000 | 1 | 1 | 0 | 0 | pQTL8 |  |  |  |  |  |
| 2 | 21500000 | 22500000 | 0 | 1 | 0 | 2 |  |  |  |  | pQTL8 |  |
| 2 | 22500000 | 23500000 | 1 | 0 | 0 | 0 |  |  |  |  |  |  |
| 2 | 23500000 | 24500000 | 0 | 0 | 0 | 0 |  |  |  |  |  |  |
| 2 | 24500000 | 25500000 | 0 | 0 | 0 | 0 |  |  |  |  |  |  |
| 2 | 25500000 | 26500000 | 0 | 1 | 1 | 2 |  |  |  | pQTL4 | pQTL9 | pQTL7 |
| 2 | 26500000 | 27500000 | 0 | 2 | 2 | 2 |  |  |  | pQTL4 | pQTL9 | pQTL7 |
| 2 | 27500000 | 28500000 | 0 | 1 | 2 | 3 |  |  |  | pQTL4 | pQTL9 | pQTL7 |
| 2 | 28500000 | 29500000 | 1 | 0 | 0 | 2 |  |  |  |  |  |  |
| 2 | 29500000 | 30500000 | 3 | 1 | 1 | 3 | pQTL9 | pQTL7 | pQTL10 | pQTL5 | pQTL10 | pQTL8 |
| 2 | 30500000 | 31500000 | 1 | 0 | 1 | 4 |  | pQTL7 | pQTL10 |  |  | pQTL8 |
| 2 | 31500000 | 32500000 | 1 | 0 | 1 | 0 |  |  |  |  |  |  |
| 2 | 32500000 | 33500000 | 1 | 1 | 0 | 3 | pQTL10 |  |  |  | pQTL11 |  |
| 2 | 33500000 | 34500000 | 1 | 2 | 2 | 6 | pQTL10 |  |  | pQTL6 | pQTL11 | pQTL9 |
| 2 | 34500000 | 35500000 | 1 | 3 | 1 | 7 |  |  |  | pQTL6 | pQTL11 | pQTL9 |
| 2 | 35500000 | 36500000 | 0 | 0 | 0 | 0 |  |  |  |  |  |  |
| 3 | 0 | 500000 | 2 | 0 | 0 | 0 |  |  |  |  |  |  |
| 3 | 500000 | 1500000 | 5 | 3 | 0 | 0 | pQTL11 |  |  |  |  |  |
| 3 | 1500000 | 2500000 | 9 | 4 | 2 | 1 | pQTL11 | pQTL8 | pQTL11 | pQTL7 |  | pQTL10 |
| 3 | 2500000 | 3500000 | 6 | 2 | 0 | 1 | pQTL11 |  |  |  |  |  |
| 3 | 3500000 | 4500000 | 4 | 1 | 0 | 1 | pQTL11 |  | pQTL12 |  |  |  |
| 3 | 4500000 | 5500000 | 4 | 2 | 1 | 0 | pQTL11 | pQTL9 |  | pQTL8 |  |  |
| 3 | 5500000 | 6500000 | 3 | 1 | 0 | 0 |  |  |  |  |  |  |
| 3 | 6500000 | 7500000 | 2 | 0 | 0 | 1 |  |  | pQTL13 |  |  |  |
| 3 | 7500000 | 8500000 | 4 | 0 | 0 | 1 |  |  | pQTL13 |  |  |  |
| 3 | 8500000 | 9500000 | 4 | 0 | 0 | 0 |  |  |  |  |  |  |
| 3 | 9500000 | 10500000 | 5 | 0 | 1 | 3 |  | pQTL10 | pQTL14 |  |  |  |
| 3 | 10500000 | 11500000 | 2 | 0 | 0 | 1 |  |  | pQTL14 |  |  |  |
| 3 | 11500000 | 12500000 | 1 | 2 | 1 | 2 |  |  | pQTL14 |  | pQTL12 | pQTL11 |
| 3 | 12500000 | 13500000 | 1 | 1 | 0 | 1 |  |  | pQTL14 |  | pQTL12 |  |
| 3 | 13500000 | 14500000 | 1 | 1 | 0 | 1 |  |  | pQTL14 |  | pQTL12 |  |
| 3 | 14500000 | 15500000 | 1 | 2 | 0 | 4 |  |  | pQTL14 |  | pQTL12 |  |
| 3 | 15500000 | 16500000 | 0 | 1 | 0 | 1 |  |  |  |  | pQTL12 |  |
| 3 | 16500000 | 17500000 | 0 | 2 | 0 | 4 |  |  |  |  | pQTL12 |  |
| 3 | 17500000 | 18500000 | 0 | 0 | 0 | 3 |  |  |  |  |  |  |
| 3 | 18500000 | 19500000 | 0 | 0 | 0 | 1 |  |  |  |  |  |  |
| 3 | 19500000 | 20500000 | 0 | 0 | 0 | 1 |  |  |  |  |  |  |
| 3 | 20500000 | 21500000 | 0 | 0 | 0 | 1 |  |  |  |  |  |  |
| 3 | 21500000 | 22500000 | 0 | 1 | 0 | 1 |  |  |  |  | pQTL13 |  |
| 3 | 22500000 | 23500000 | 0 | 2 | 0 | 3 |  |  |  |  | pQTL13 |  |
| 3 | 23500000 | 24500000 | 1 | 0 | 0 | 1 |  |  |  |  |  |  |
| 3 | 24500000 | 25500000 | 2 | 0 | 0 | 1 |  |  |  |  |  |  |
| 3 | 25500000 | 26500000 | 1 | 0 | 0 | 0 |  |  |  |  |  |  |
| 3 | 26500000 | 27500000 | 1 | 2 | 3 | 3 |  |  |  | pQTL9 | pQTL14 | pQTL12 |
| 3 | 27500000 | 28500000 | 0 | 4 | 5 | 7 |  |  |  | pQTL9 | pQTL14 | pQTL12 |
| 3 | 28500000 | 29500000 | 2 | 1 | 0 | 4 |  |  | pQTL15 |  | pQTL14 |  |
| 3 | 29500000 | 30500000 | 2 | 1 | 0 | 1 | pQTL12 |  | pQTL15 |  | pQTL14 |  |
| 3 | 30500000 | 31500000 | 5 | 6 | 3 | 3 | pQTL12 | pQTL11 | pQTL15 | pQTL10 | pQTL14 | pQTL13 |
| 3 | 31500000 | 32500000 | 3 | 2 | 0 | 1 | pQTL12 |  | pQTL15 |  | pQTL14 |  |
| 3 | 32500000 | 33500000 | 4 | 1 | 1 | 4 | pQTL12 | pQTL12 | pQTL15 | pQTL11 | pQTL14 | pQTL14 |
| 3 | 33500000 | 34500000 | 0 | 1 | 0 | 2 |  |  |  |  | pQTL14 |  |
| 3 | 34500000 | 35500000 | 0 | 6 | 0 | 3 |  |  |  |  | pQTL14 |  |
| 3 | 35500000 | 36500000 | 0 | 6 | 0 | 6 |  |  |  |  | pQTL14 |  |
| 3 | 36500000 | 37500000 | 0 | 0 | 0 | 0 |  |  |  |  |  |  |
| 4 | 0 | 500000 | 1 | 1 | 0 | 0 |  |  |  |  |  |  |
| 4 | 500000 | 1500000 | 0 | 4 | 0 | 5 |  |  |  |  | pQTL15 |  |
| 4 | 1500000 | 2500000 | 0 | 0 | 0 | 0 |  |  |  |  |  |  |
| 4 | 2500000 | 3500000 | 1 | 0 | 0 | 0 |  |  |  |  |  |  |
| 4 | 3500000 | 4500000 | 1 | 0 | 0 | 0 |  |  |  |  |  |  |
| 4 | 6500000 | 7500000 | 0 | 1 | 0 | 1 |  |  |  |  |  |  |
| 4 | 7500000 | 8500000 | 0 | 1 | 0 | 0 |  |  |  |  |  |  |
| 4 | 8500000 | 9500000 | 0 | 1 | 0 | 0 |  |  |  |  |  |  |
| 4 | 9500000 | 10500000 | 0 | 0 | 0 | 0 |  |  |  |  |  |  |
| 4 | 10500000 | 11500000 | 0 | 0 | 0 | 0 |  |  |  |  |  |  |
| 4 | 11500000 | 12500000 | 0 | 0 | 0 | 0 |  |  |  |  |  |  |
| 4 | 12500000 | 13500000 | 1 | 1 | 0 | 0 | pQTL13 |  |  |  |  |  |
| 4 | 13500000 | 14500000 | 1 | 0 | 0 | 0 |  |  |  |  |  |  |
| 4 | 17500000 | 18500000 | 0 | 1 | 0 | 0 |  |  |  |  |  |  |
| 4 | 18500000 | 19500000 | 0 | 1 | 0 | 0 |  |  |  |  |  |  |
| 4 | 19500000 | 20500000 | 0 | 3 | 0 | 0 |  |  |  |  |  |  |
| 4 | 20500000 | 21500000 | 0 | 1 | 0 | 1 |  |  |  |  |  |  |
| 4 | 21500000 | 22500000 | 0 | 0 | 0 | 2 |  |  |  |  |  |  |
| 4 | 22500000 | 23500000 | 0 | 0 | 0 | 1 |  |  |  |  |  |  |
| 4 | 23500000 | 24500000 | 0 | 1 | 1 | 2 |  |  |  |  | pQTL16 |  |
| 4 | 24500000 | 25500000 | 0 | 1 | 1 | 0 |  |  |  |  |  |  |
| 4 | 25500000 | 26500000 | 0 | 0 | 1 | 0 |  |  |  |  |  |  |
| 4 | 26500000 | 27500000 | 0 | 1 | 1 | 0 |  |  |  | pQTL12 |  |  |
| 4 | 27500000 | 28500000 | 0 | 1 | 3 | 0 |  |  |  | pQTL12 |  |  |
| 4 | 28500000 | 29500000 | 2 | 4 | 2 | 0 | pQTL14 | pQTL13 |  | pQTL12 |  |  |
| 4 | 29500000 | 30500000 | 2 | 2 | 1 | 0 | pQTL14 | pQTL13 |  | pQTL12 |  |  |
| 4 | 30500000 | 31500000 | 3 | 5 | 1 | 0 | pQTL14 | pQTL13 |  | pQTL12 |  |  |
| 4 | 31500000 | 32500000 | 2 | 7 | 1 | 1 |  |  |  | pQTL12 | pQTL17 |  |
| 4 | 32500000 | 33500000 | 1 | 4 | 0 | 1 |  |  |  |  | pQTL17 |  |
| 4 | 33500000 | 34500000 | 1 | 2 | 0 | 1 |  |  | pQTL16 |  |  |  |
| 4 | 34500000 | 35500000 | 1 | 1 | 0 | 2 |  |  | pQTL16 |  |  |  |
| 5 | 0 | 500000 | 3 | 3 | 0 | 2 | pQTL15 |  | pQTL16 |  | pQTL18 |  |
| 5 | 500000 | 1500000 | 3 | 0 | 1 | 1 |  | pQTL14 | pQTL16 |  |  | pQTL15 |
| 5 | 1500000 | 2500000 | 3 | 1 | 2 | 2 | pQTL16 | pQTL14 | pQTL16 |  |  | pQTL15 |
| 5 | 2500000 | 3500000 | 2 | 0 | 1 | 3 |  | pQTL14 | pQTL16 |  |  | pQTL15 |
| 5 | 3500000 | 4500000 | 0 | 1 | 0 | 2 |  |  |  |  |  |  |
| 5 | 4500000 | 5500000 | 0 | 0 | 0 | 2 |  |  |  |  |  |  |
| 5 | 5500000 | 6500000 | 1 | 0 | 0 | 2 |  |  | pQTL17 |  |  |  |
| 5 | 6500000 | 7500000 | 0 | 1 | 2 | 4 |  |  |  | pQTL13 | pQTL19 | pQTL16 |
| 5 | 7500000 | 8500000 | 1 | 0 | 0 | 2 |  |  | pQTL18 |  |  |  |
| 5 | 8500000 | 9500000 | 0 | 0 | 0 | 1 |  |  |  |  |  |  |
| 5 | 9500000 | 10500000 | 0 | 0 | 0 | 1 |  |  |  |  |  |  |
| 5 | 10500000 | 11500000 | 0 | 0 | 0 | 1 |  |  |  |  |  |  |
| 5 | 14500000 | 15500000 | 0 | 0 | 0 | 2 |  |  |  |  |  |  |
| 5 | 16500000 | 17500000 | 0 | 0 | 0 | 1 |  |  |  |  |  |  |
| 5 | 17500000 | 18500000 | 0 | 0 | 0 | 1 |  |  |  |  |  |  |
| 5 | 18500000 | 19500000 | 0 | 1 | 1 | 1 |  |  |  |  | pQTL20 | pQTL17 |
| 5 | 19500000 | 20500000 | 1 | 0 | 0 | 2 |  |  | pQTL19 |  |  |  |
| 5 | 20500000 | 21500000 | 1 | 0 | 0 | 0 |  |  |  |  |  |  |
| 5 | 21500000 | 22500000 | 1 | 1 | 0 | 1 | pQTL17 |  | pQTL20 |  | pQTL21 |  |
| 5 | 22500000 | 23500000 | 0 | 1 | 0 | 1 |  |  |  |  | pQTL21 |  |
| 5 | 23500000 | 24500000 | 1 | 1 | 0 | 2 |  |  | pQTL21 |  | pQTL21 |  |
| 5 | 24500000 | 25500000 | 2 | 2 | 0 | 1 | pQTL18 |  | pQTL21 |  | pQTL21 |  |
| 5 | 25500000 | 26500000 | 3 | 4 | 2 | 2 | pQTL18 |  | pQTL21 | pQTL14 | pQTL21 | pQTL18 |
| 5 | 26500000 | 27500000 | 3 | 3 | 1 | 2 | pQTL18 |  | pQTL21 | pQTL14 | pQTL21 | pQTL18 |
| 5 | 27500000 | 28500000 | 2 | 2 | 1 | 1 |  |  | pQTL21 | pQTL14 |  |  |
| 5 | 28500000 | 29500000 | 0 | 3 | 1 | 0 |  |  |  |  |  |  |
| 6 | 0 | 500000 | 3 | 2 | 0 | 1 | pQTL19 |  | pQTL22 |  | pQTL22 |  |
| 6 | 500000 | 1500000 | 7 | 2 | 1 | 1 | pQTL19 | pQTL15 | pQTL22 |  | pQTL22 | pQTL19 |
| 6 | 1500000 | 2500000 | 4 | 3 | 0 | 1 | pQTL19 |  | pQTL22 |  | pQTL22 |  |
| 6 | 2500000 | 3500000 | 7 | 7 | 0 | 4 | pQTL19 |  | pQTL22 |  | pQTL22 |  |
| 6 | 3500000 | 4500000 | 3 | 6 | 0 | 2 | pQTL19 |  | pQTL22 |  | pQTL22 |  |
| 6 | 4500000 | 5500000 | 2 | 6 | 1 | 3 | pQTL19 | pQTL16 | pQTL22 | pQTL15 | pQTL22 | pQTL20 |
| 6 | 5500000 | 6500000 | 4 | 10 | 1 | 7 | pQTL19 | pQTL16 | pQTL22 | pQTL15 | pQTL22 | pQTL20 |
| 6 | 6500000 | 7500000 | 3 | 5 | 0 | 3 | pQTL19 |  | pQTL22 |  | pQTL22 |  |
| 6 | 7500000 | 8500000 | 5 | 5 | 0 | 3 | pQTL19 |  | pQTL22 |  | pQTL22 |  |
| 6 | 8500000 | 9500000 | 7 | 6 | 2 | 1 | pQTL19 | pQTL17 | pQTL22 | pQTL16 | pQTL22 |  |
| 6 | 9500000 | 10500000 | 3 | 1 | 0 | 3 | pQTL19 |  | pQTL22 |  | pQTL22 |  |
| 6 | 10500000 | 11500000 | 2 | 0 | 0 | 0 |  |  |  |  |  |  |
| 6 | 11500000 | 12500000 | 2 | 0 | 0 | 0 |  |  |  |  |  |  |
| 6 | 15500000 | 16500000 | 0 | 1 | 0 | 0 |  |  |  |  |  |  |
| 6 | 16500000 | 17500000 | 0 | 1 | 0 | 0 |  |  |  |  |  |  |
| 6 | 17500000 | 18500000 | 0 | 1 | 0 | 0 |  |  |  |  |  |  |
| 6 | 18500000 | 19500000 | 1 | 2 | 0 | 2 | pQTL20 |  | pQTL23 |  | pQTL23 |  |
| 6 | 19500000 | 20500000 | 1 | 2 | 0 | 2 | pQTL20 |  | pQTL23 |  | pQTL23 |  |
| 6 | 20500000 | 21500000 | 1 | 1 | 0 | 1 | pQTL20 |  | pQTL23 |  | pQTL23 |  |
| 6 | 21500000 | 22500000 | 1 | 1 | 0 | 0 | pQTL20 |  |  |  |  |  |
| 6 | 22500000 | 23500000 | 2 | 1 | 0 | 0 | pQTL20 |  |  |  |  |  |
| 6 | 23500000 | 24500000 | 3 | 1 | 0 | 0 | pQTL20 |  |  |  |  |  |
| 6 | 24500000 | 25500000 | 1 | 0 | 0 | 1 |  |  |  |  |  |  |
| 6 | 25500000 | 26500000 | 1 | 0 | 1 | 0 |  |  |  |  |  |  |
| 6 | 26500000 | 27500000 | 3 | 3 | 1 | 3 | pQTL21 |  | pQTL24 | pQTL17 | pQTL24 | pQTL21 |
| 6 | 27500000 | 28500000 | 2 | 2 | 1 | 2 | pQTL21 |  | pQTL24 | pQTL17 | pQTL24 | pQTL21 |
| 6 | 28500000 | 29500000 | 3 | 6 | 3 | 6 | pQTL21 | pQTL18 | pQTL24 | pQTL17 | pQTL24 | pQTL21 |
| 6 | 29500000 | 30500000 | 0 | 4 | 1 | 3 |  |  |  | pQTL17 | pQTL24 | pQTL21 |
| 6 | 30500000 | 31500000 | 0 | 3 | 0 | 6 |  |  |  |  | pQTL24 |  |
| 6 | 31500000 | 32500000 | 0 | 0 | 0 | 0 |  |  |  |  |  |  |
| 7 | 500000 | 1500000 | 0 | 0 | 0 | 1 |  |  |  |  |  |  |
| 7 | 1500000 | 2500000 | 0 | 0 | 0 | 3 |  |  |  |  |  |  |
| 7 | 2500000 | 3500000 | 0 | 1 | 0 | 3 |  |  |  |  | pQTL25 |  |
| 7 | 3500000 | 4500000 | 2 | 0 | 0 | 1 |  |  |  |  |  |  |
| 7 | 4500000 | 5500000 | 1 | 0 | 0 | 1 |  |  |  |  |  |  |
| 7 | 5500000 | 6500000 | 2 | 0 | 1 | 1 |  | pQTL19 |  |  |  |  |
| 7 | 6500000 | 7500000 | 3 | 0 | 0 | 1 |  |  | pQTL25 |  |  |  |
| 7 | 7500000 | 8500000 | 2 | 0 | 0 | 0 |  |  |  |  |  |  |
| 7 | 8500000 | 9500000 | 4 | 1 | 2 | 0 | pQTL22 | pQTL20 |  | pQTL18 |  |  |
| 7 | 9500000 | 10500000 | 1 | 1 | 2 | 0 | pQTL22 | pQTL20 |  | pQTL18 |  |  |
| 7 | 10500000 | 11500000 | 1 | 1 | 2 | 0 | pQTL22 | pQTL20 |  | pQTL18 |  |  |
| 7 | 11500000 | 12500000 | 2 | 1 | 2 | 0 | pQTL22 | pQTL20 |  | pQTL18 |  |  |
| 7 | 12500000 | 13500000 | 4 | 3 | 2 | 0 | pQTL22 | pQTL20 |  | pQTL18 |  |  |
| 7 | 13500000 | 14500000 | 2 | 0 | 0 | 0 |  |  |  |  |  |  |
| 7 | 14500000 | 15500000 | 2 | 0 | 0 | 0 |  |  |  |  |  |  |
| 7 | 15500000 | 16500000 | 2 | 0 | 0 | 3 |  |  | pQTL26 |  |  |  |
| 7 | 16500000 | 17500000 | 1 | 0 | 0 | 0 |  |  |  |  |  |  |
| 7 | 17500000 | 18500000 | 0 | 0 | 0 | 0 |  |  |  |  |  |  |
| 7 | 18500000 | 19500000 | 1 | 1 | 2 | 2 |  | pQTL21 | pQTL27 |  |  | pQTL22 |
| 7 | 19500000 | 20500000 | 0 | 1 | 1 | 1 |  |  |  |  | pQTL26 |  |
| 7 | 20500000 | 21500000 | 0 | 2 | 0 | 4 |  |  |  |  | pQTL26 |  |
| 7 | 21500000 | 22500000 | 0 | 1 | 0 | 2 |  |  |  |  | pQTL26 |  |
| 7 | 22500000 | 23500000 | 1 | 1 | 1 | 0 | pQTL23 | pQTL22 |  | pQTL19 |  |  |
| 7 | 23500000 | 24500000 | 0 | 1 | 1 | 0 |  |  |  | pQTL19 |  |  |
| 7 | 24500000 | 25500000 | 4 | 1 | 1 | 1 | pQTL24 | pQTL23 | pQTL28 | pQTL19 | pQTL27 | pQTL23 |
| 7 | 25500000 | 26500000 | 2 | 2 | 4 | 5 | pQTL24 | pQTL23 | pQTL28 | pQTL19 | pQTL27 | pQTL23 |
| 7 | 26500000 | 27500000 | 3 | 3 | 0 | 1 | pQTL24 |  | pQTL28 |  | pQTL27 |  |
| 7 | 27500000 | 28500000 | 5 | 4 | 0 | 0 | pQTL24 |  |  |  |  |  |
| 7 | 28500000 | 29500000 | 6 | 4 | 0 | 0 | pQTL24 |  |  |  |  |  |
| 7 | 29500000 | 30500000 | 4 | 2 | 0 | 0 |  |  |  |  |  |  |
| 8 | 0 | 500000 | 1 | 0 | 0 | 0 |  |  |  |  |  |  |
| 8 | 500000 | 1500000 | 1 | 1 | 0 | 0 |  |  |  |  |  |  |
| 8 | 1500000 | 2500000 | 5 | 3 | 0 | 0 |  |  |  |  |  |  |
| 8 | 2500000 | 3500000 | 2 | 2 | 0 | 0 |  |  |  |  |  |  |
| 8 | 3500000 | 4500000 | 5 | 0 | 0 | 0 |  |  |  |  |  |  |
| 8 | 4500000 | 5500000 | 5 | 1 | 2 | 1 | pQTL25 | pQTL24 | pQTL29 | pQTL20 |  |  |
| 8 | 5500000 | 6500000 | 3 | 0 | 1 | 0 |  | pQTL24 |  |  |  |  |
| 8 | 6500000 | 7500000 | 1 | 0 | 0 | 0 |  |  |  |  |  |  |
| 8 | 7500000 | 8500000 | 1 | 0 | 0 | 0 |  |  |  |  |  |  |
| 8 | 9500000 | 10500000 | 0 | 1 | 0 | 0 |  |  |  |  |  |  |
| 8 | 10500000 | 11500000 | 0 | 1 | 0 | 0 |  |  |  |  |  |  |
| 8 | 11500000 | 12500000 | 0 | 1 | 0 | 1 |  |  |  |  | pQTL28 |  |
| 8 | 12500000 | 13500000 | 0 | 1 | 0 | 1 |  |  |  |  | pQTL28 |  |
| 8 | 13500000 | 14500000 | 0 | 1 | 0 | 1 |  |  |  |  | pQTL28 |  |
| 8 | 14500000 | 15500000 | 0 | 1 | 1 | 1 |  |  |  |  | pQTL28 |  |
| 8 | 16500000 | 17500000 | 1 | 1 | 0 | 0 | pQTL26 |  |  |  |  |  |
| 8 | 17500000 | 18500000 | 1 | 0 | 0 | 0 |  |  |  |  |  |  |
| 8 | 18500000 | 19500000 | 2 | 0 | 0 | 0 |  |  |  |  |  |  |
| 8 | 19500000 | 20500000 | 1 | 1 | 0 | 0 | pQTL27 |  |  |  |  |  |
| 8 | 20500000 | 21500000 | 2 | 2 | 0 | 2 | pQTL27 |  | pQTL30 |  | pQTL29 |  |
| 8 | 21500000 | 22500000 | 2 | 1 | 0 | 2 | pQTL27 |  | pQTL30 |  | pQTL29 |  |
| 8 | 22500000 | 23500000 | 0 | 1 | 0 | 1 |  |  |  |  |  |  |
| 8 | 23500000 | 24500000 | 0 | 2 | 0 | 1 |  |  |  |  |  |  |
| 8 | 24500000 | 25500000 | 1 | 4 | 0 | 1 | pQTL28 |  | pQTL31 |  | pQTL30 |  |
| 8 | 25500000 | 26500000 | 1 | 1 | 0 | 0 | pQTL28 |  |  |  |  |  |
| 8 | 26500000 | 27500000 | 2 | 2 | 1 | 3 | pQTL28 | pQTL25 | pQTL32 | pQTL21 | pQTL31 | pQTL24 |
| 8 | 27500000 | 28500000 | 2 | 1 | 1 | 1 | pQTL28 | pQTL25 | pQTL32 | pQTL21 | pQTL31 | pQTL24 |
| 9 | 500000 | 1500000 | 0 | 1 | 0 | 0 |  |  |  |  |  |  |
| 9 | 1500000 | 2500000 | 0 | 1 | 0 | 0 |  |  |  |  |  |  |
| 9 | 2500000 | 3500000 | 0 | 0 | 0 | 0 |  |  |  |  |  |  |
| 9 | 3500000 | 4500000 | 0 | 0 | 0 | 0 |  |  |  |  |  |  |
| 9 | 4500000 | 5500000 | 0 | 0 | 0 | 0 |  |  |  |  |  |  |
| 9 | 5500000 | 6500000 | 0 | 0 | 0 | 1 |  |  |  |  |  |  |
| 9 | 6500000 | 7500000 | 1 | 0 | 0 | 1 |  |  | pQTL33 |  |  |  |
| 9 | 7500000 | 8500000 | 1 | 1 | 0 | 1 | pQTL29 |  | pQTL33 |  | pQTL32 |  |
| 9 | 8500000 | 9500000 | 2 | 1 | 0 | 1 | pQTL29 |  | pQTL33 |  | pQTL32 |  |
| 9 | 9500000 | 10500000 | 2 | 1 | 0 | 1 | pQTL29 |  | pQTL33 |  | pQTL32 |  |
| 9 | 10500000 | 11500000 | 1 | 1 | 0 | 1 | pQTL29 |  | pQTL33 |  | pQTL32 |  |
| 9 | 11500000 | 12500000 | 1 | 1 | 0 | 0 |  |  |  |  |  |  |
| 9 | 12500000 | 13500000 | 1 | 1 | 0 | 0 |  |  |  |  |  |  |
| 9 | 13500000 | 14500000 | 1 | 0 | 0 | 1 |  |  |  |  |  |  |
| 9 | 14500000 | 15500000 | 1 | 1 | 0 | 1 | pQTL30 |  | pQTL34 |  | pQTL33 |  |
| 9 | 15500000 | 16500000 | 2 | 1 | 0 | 0 | pQTL30 |  |  |  |  |  |
| 9 | 16500000 | 17500000 | 1 | 1 | 0 | 0 | pQTL30 |  |  |  |  |  |
| 9 | 17500000 | 18500000 | 2 | 4 | 0 | 2 | pQTL30 |  | pQTL35 |  | pQTL34 |  |
| 9 | 18500000 | 19500000 | 5 | 5 | 2 | 3 | pQTL30 | pQTL26 | pQTL35 | pQTL22 | pQTL34 | pQTL25 |
| 9 | 19500000 | 20500000 | 4 | 6 | 2 | 2 | pQTL30 | pQTL26 | pQTL35 | pQTL22 | pQTL34 | pQTL25 |
| 9 | 20500000 | 21500000 | 3 | 1 | 0 | 1 | pQTL30 |  | pQTL35 |  | pQTL34 |  |
| 9 | 21500000 | 22500000 | 0 | 1 | 0 | 1 |  |  |  |  | pQTL34 |  |
| 9 | 22500000 | 23500000 | 0 | 0 | 0 | 0 |  |  |  |  |  |  |
| 10 | 1500000 | 2500000 | 1 | 2 | 0 | 0 | pQTL31 |  |  |  |  |  |
| 10 | 2500000 | 3500000 | 1 | 0 | 0 | 0 |  |  |  |  |  |  |
| 10 | 3500000 | 4500000 | 1 | 0 | 0 | 0 |  |  |  |  |  |  |
| 10 | 4500000 | 5500000 | 3 | 1 | 1 | 1 | pQTL32 | pQTL27 | pQTL36 | pQTL23 | pQTL35 | pQTL26 |
| 10 | 5500000 | 6500000 | 1 | 1 | 0 | 0 | pQTL32 |  |  |  |  |  |
| 10 | 6500000 | 7500000 | 1 | 1 | 0 | 0 | pQTL32 |  |  |  |  |  |
| 10 | 7500000 | 8500000 | 1 | 1 | 0 | 0 | pQTL32 |  |  |  |  |  |
| 10 | 8500000 | 9500000 | 1 | 1 | 0 | 0 | pQTL32 |  |  |  |  |  |
| 10 | 9500000 | 10500000 | 2 | 1 | 1 | 0 | pQTL32 | pQTL28 |  | pQTL24 |  |  |
| 10 | 12500000 | 13500000 | 1 | 0 | 0 | 0 |  |  |  |  |  |  |
| 10 | 13500000 | 14500000 | 0 | 1 | 0 | 1 |  |  |  |  | pQTL36 |  |
| 10 | 14500000 | 15500000 | 0 | 0 | 0 | 0 |  |  |  |  |  |  |
| 10 | 15500000 | 16500000 | 2 | 2 | 2 | 1 | pQTL33 | pQTL29 | pQTL37 | pQTL25 | pQTL37 |  |
| 10 | 16500000 | 17500000 | 1 | 0 | 0 | 2 |  |  | pQTL37 |  |  |  |
| 10 | 17500000 | 18500000 | 3 | 0 | 1 | 5 |  | pQTL30 | pQTL37 |  |  | pQTL27 |
| 10 | 18500000 | 19500000 | 2 | 0 | 1 | 6 |  |  | pQTL37 |  |  | pQTL27 |
| 10 | 19500000 | 20500000 | 1 | 0 | 1 | 3 |  |  | pQTL37 |  |  | pQTL27 |
| 10 | 20500000 | 21500000 | 1 | 4 | 1 | 1 | pQTL34 | pQTL31 | pQTL37 | pQTL26 | pQTL38 | pQTL27 |
| 10 | 21500000 | 22500000 | 1 | 0 | 1 | 1 |  | pQTL31 | pQTL37 |  |  | pQTL27 |
| 10 | 22500000 | 23500000 | 0 | 1 | 0 | 2 |  |  |  |  |  |  |
| 11 | 0 | 500000 | 1 | 0 | 0 | 0 |  |  |  |  |  |  |
| 11 | 500000 | 1500000 | 1 | 0 | 0 | 1 |  |  |  |  |  |  |
| 11 | 1500000 | 2500000 | 1 | 0 | 0 | 0 |  |  |  |  |  |  |
| 11 | 2500000 | 3500000 | 0 | 0 | 0 | 1 |  |  |  |  |  |  |
| 11 | 3500000 | 4500000 | 1 | 1 | 0 | 1 | pQTL35 |  | pQTL38 |  |  |  |
| 11 | 4500000 | 5500000 | 1 | 0 | 0 | 1 |  |  | pQTL38 |  |  |  |
| 11 | 5500000 | 6500000 | 2 | 0 | 0 | 0 |  |  |  |  |  |  |
| 11 | 6500000 | 7500000 | 2 | 0 | 0 | 0 |  |  |  |  |  |  |
| 11 | 7500000 | 8500000 | 2 | 0 | 0 | 1 |  |  | pQTL39 |  |  |  |
| 11 | 8500000 | 9500000 | 2 | 0 | 0 | 0 |  |  |  |  |  |  |
| 11 | 9500000 | 10500000 | 1 | 0 | 0 | 0 |  |  |  |  |  |  |
| 11 | 16500000 | 17500000 | 1 | 0 | 3 | 0 |  |  |  |  |  |  |
| 11 | 17500000 | 18500000 | 1 | 0 | 0 | 2 |  |  | pQTL40 |  |  |  |
| 11 | 18500000 | 19500000 | 0 | 0 | 0 | 1 |  |  |  |  |  |  |
| 11 | 19500000 | 20500000 | 1 | 0 | 0 | 0 |  |  |  |  |  |  |
| 11 | 20500000 | 21500000 | 1 | 1 | 0 | 0 |  |  |  |  |  |  |
| 11 | 21500000 | 22500000 | 0 | 1 | 0 | 0 |  |  |  |  |  |  |
| 11 | 22500000 | 23500000 | 0 | 1 | 0 | 0 |  |  |  |  |  |  |
| 11 | 23500000 | 24500000 | 0 | 1 | 1 | 0 |  |  |  |  |  |  |
| 11 | 24500000 | 25500000 | 0 | 0 | 1 | 0 |  |  |  |  |  |  |
| 11 | 25500000 | 26500000 | 0 | 1 | 1 | 0 |  |  |  |  |  |  |
| 11 | 26500000 | 27500000 | 0 | 1 | 1 | 0 |  |  |  |  |  |  |
| 11 | 27500000 | 28500000 | 1 | 2 | 1 | 3 |  | pQTL32 | pQTL41 |  |  |  |
| 11 | 28500000 | 29500000 | 0 | 1 | 0 | 0 |  |  |  |  |  |  |
| 11 | 39500000 | 40500000 | 1 | 0 | 0 | 0 |  |  |  |  |  |  |
| 12 | 500000 | 1500000 | 1 | 1 | 0 | 3 |  |  |  |  |  |  |
| 12 | 1500000 | 2500000 | 0 | 2 | 1 | 0 |  |  |  |  |  |  |
| 12 | 2500000 | 3500000 | 1 | 0 | 0 | 0 |  |  |  |  |  |  |
| 12 | 3500000 | 4500000 | 0 | 0 | 1 | 0 |  |  |  |  |  |  |
| 12 | 8500000 | 9500000 | 0 | 1 | 0 | 0 |  |  |  |  |  |  |
| 12 | 9500000 | 10500000 | 0 | 1 | 1 | 0 |  |  |  |  |  |  |
| 12 | 13500000 | 14500000 | 0 | 0 | 0 | 1 |  |  |  |  |  |  |
| 12 | 15500000 | 16500000 | 0 | 0 | 0 | 0 |  |  |  |  |  |  |
| 12 | 16500000 | 17500000 | 0 | 3 | 0 | 3 |  |  |  |  | pQTL39 |  |
| 12 | 17500000 | 18500000 | 0 | 0 | 0 | 1 |  |  |  |  |  |  |
| 12 | 18500000 | 19500000 | 0 | 0 | 0 | 1 |  |  |  |  |  |  |
| 12 | 19500000 | 20500000 | 1 | 0 | 0 | 2 |  |  |  |  |  |  |
| 12 | 20500000 | 21500000 | 0 | 1 | 0 | 2 |  |  |  |  | pQTL40 |  |
| 12 | 21500000 | 22500000 | 1 | 2 | 0 | 2 | pQTL36 |  | pQTL42 |  | pQTL40 |  |
| 12 | 22500000 | 23500000 | 0 | 1 | 0 | 1 |  |  |  |  | pQTL40 |  |
| 12 | 23500000 | 24500000 | 0 | 1 | 0 | 1 |  |  |  |  | pQTL40 |  |
| 12 | 24500000 | 25500000 | 0 | 1 | 1 | 1 |  |  |  | pQTL27 | pQTL40 | pQTL28 |
| 12 | 25500000 | 26500000 | 0 | 1 | 7 | 2 |  |  |  | pQTL27 | pQTL40 | pQTL28 |
| 12 | 26500000 | 27500000 | 0 | 0 | 0 | 2 |  |  |  |  |  |  |

pQTL: pleiotropic quantitative trait loci; HD: heading date; GNP: grain number per plant; PN: panicle number; KGW: kilo-grain weight.
