# Supplementary material for: Genetic Basis Underlying Correlations Among Growth Duration and Yield Traits Revealed by GWAS in Rice (Oryza sativa L.)
Source: Front Plant Sci. 2018 May 22;9:650. doi: 10.3389/fpls.2018.00650 (PMC5972282; doi:10.3389/fpls.2018.00650)
Supplement: Supplementary file 8 [file Table_8.DOCX]

**SUPPLEMENTARY TABLE 8 | Numbers of pleiotropic SNPs, pleiotropic QTLs and pleiotropic genes among different traits.**

| **Trait** | **pQTL** | | | | **pSNP** | **Candidate pGene** | | | | | **Candidate pGene with pSNP** | | | | | **Pathway** | | | | | |
| --- | --- | --- | --- | --- | --- | --- | --- | --- | --- | --- | --- | --- | --- | --- | --- | --- | --- | --- | --- | --- | --- |
|  |  |  |  |  |  | **pQTL** | | | | **Candidate gene** | **pQTL** | | | | **Candidate gene** | **Pathway** | **Pathways containing iGenes** | **Pairs of iGenes** | | | |
|  | **Total** | **Full** | ***Ind.*** | ***Jap.*** |  | **Total** | **Full** | ***Ind.*** | ***Jap.*** |  | **Total** | **Full** | ***Ind.*** | ***Jap.*** |  |  |  | **Total** | **Full** | ***Ind.*** | ***Jap.*** |
| HD GNP | 16 | 10 | 7 | 4 | 26 | 8 | 5 | 2 | 3 | 22 | 5 | 4 | 2 | 2 | 12 | 12 | 5 | 12 | 8 | 3 | 0 |
| HD PN | 9 | 5 | 4 | 0 | 1 | 2 | 1 | 1 | 0 | 5 | 1 | 1 | 0 | 0 | 2 | 9 | 2 | 3 | 3 | 0 | 0 |
| HD KGW | 9 | 4 | 7 | 0 | 0 | 1 | 0 | 1 | 0 | 1 | 0 | 0 | 0 | 0 | 0 | 8 | 3 | 4 | 1 | 1 | 1 |
| GNP PN | 8 | 6 | 2 | 2 | 2 | 4 | 3 | 1 | 2 | 15 | 2 | 1 | 1 | 1 | 2 | 10 | 5 | 7 | 3 | 1 | 0 |
| GNP KGW | 11 | 10 | 0 | 1 | 0 | 2 | 2 | 0 | 0 | 2 | 0 | 0 | 0 | 0 | 0 | 9 | 4 | 6 | 4 | 0 | 2 |
| PN KGW | 11 | 7 | 4 | 0 | 8 | 2 | 2 | 0 | 0 | 7 | 1 | 1 | 0 | 0 | 4 | 7 | 4 | 8 | 5 | 1 | 1 |

pSNP: pleiotropic SNP; pQTL: pleiotropic QTL; pGene: pleiotropic gene; *Ind*.:*indica*; *Jap*.:*japonica*; HD: heading date; GNP: grain number per plant; PN: panicle number; KGW: kilo-grain weight.
