# Supplementary material for: Genetic Basis Underlying Correlations Among Growth Duration and Yield Traits Revealed by GWAS in Rice (Oryza sativa L.)
Source: Front Plant Sci. 2018 May 22;9:650. doi: 10.3389/fpls.2018.00650 (PMC5972282; doi:10.3389/fpls.2018.00650)
Supplement: Supplementary file 9 [file Table_9.DOCX]

**SUPPLEMENTARY TABLE 9 | Fifteen pathways regulating multiple traits identified by genome-wide association study.**

| **Pathway** | **Associated gene** | | **Position** | | | **Cloned gene** | | **HD** | **GNP** | **PN** | **KGW** |
| --- | --- | --- | --- | --- | --- | --- | --- | --- | --- | --- | --- |
| Arginine and proline metabolism  (1 cloned gene + 6 candidate genes) | Os02g0611200 | LOC_Os02g39790 | 2 | 24045246 | 24048446 |  |  |  | GNP |  |  |
|  | Os04g0106300 | LOC_Os04g01590 | 4 | 396481 | 400947 | *OsARG* | GNP |  |  |  |  |
|  | Os04g0182875 | LOC_Os04g10410 | 4 | 5644929 | 5649613 |  |  |  | GNP |  |  |
|  | Os04g0184500 | LOC_Os04g10569 | 4 | 5739440 | 5741093 |  |  | HD | GNP |  |  |
|  | Os06g0131300 | LOC_Os06g04070 | 6 | 1674045 | 1677406 |  |  |  |  |  | KGW |
|  | Os07g0408700 | LOC_Os07g22600 | 7 | 12716456 | 12722911 |  |  |  | GNP |  |  |
|  | Os11g0186200 | LOC_Os11g08300 | 11 | 4375281 | 4380044 |  |  |  |  | PN |  |
| Carbon fixation in photosynthetic organisms  (1 cloned gene + 4 candidate genes) | Os01g0866400 | LOC_Os01g64660 | 1 | 37519542 | 37522613 | *MOC2; FBP1* | PN |  |  |  |  |
|  | Os02g0665000 | LOC_Os02g44550 | 2 | 26988678 | 26993990 |  |  |  | GNP |  |  |
|  | Os03g0169100 | LOC_Os03g07300 | 3 | 3713491 | 3717958 |  |  |  |  |  | KGW |
|  | Os05g0186300 | LOC_Os05g09440 | 5 | 5293555 | 5297419 |  |  | HD |  |  |  |
|  | Os06g0133800 | LOC_Os06g04270 | 6 | 1808766 | 1812362 |  |  |  |  |  | KGW |
| Carbon metabolism  (1 cloned gene + 12 candidate genes) | Os01g0328700 | LOC_Os01g22520 | 1 | 12654852 | 12658475 |  |  | HD |  |  |  |
|  | Os01g0866400 | LOC_Os01g64660 | 1 | 37519542 | 37522613 | *MOC2; FBP1* | PN |  |  |  |  |
|  | Os02g0665000 | LOC_Os02g44550 | 2 | 26988678 | 26993990 |  |  |  | GNP |  |  |
|  | Os03g0169100 | LOC_Os03g07300 | 3 | 3713491 | 3717958 |  |  |  |  |  | KGW |
|  | Os03g0185000 | LOC_Os03g08660 | 3 | 4464190 | 4465095 |  |  | HD |  |  |  |
|  | Os03g0325000 | LOC_Os03g20880 | 3 | 11819962 | 11823440 |  |  |  |  |  | KGW |
|  | Os04g0390000 | LOC_Os04g32020 | 4 | 19175703 | 19182548 |  |  |  |  | PN |  |
|  | Os05g0156700 | LOC_Os05g06460 | 5 | 3313391 | 3319717 |  |  |  |  |  | KGW |
|  | Os05g0186300 | LOC_Os05g09440 | 5 | 5293555 | 5297419 |  |  | HD |  |  |  |
|  | Os05g0194900 | LOC_Os05g10650 | 5 | 5813265 | 5815545 |  |  |  |  | PN |  |
|  | Os06g0133800 | LOC_Os06g04270 | 6 | 1808766 | 1812362 |  |  |  |  |  | KGW |
|  | Os07g0406300 | LOC_Os07g22350 | 7 | 12544210 | 12550060 |  |  |  | GNP | PN |  |
|  | Os11g0629500 | LOC_Os11g41160 | 11 | 24655805 | 24659328 |  |  |  |  | PN |  |
| Carotenoid biosynthesis  (2 cloned genes +3 candidate genes) | Os04g0550600 | LOC_Os04g46470 | 4 | 27567824 | 27570926 | *HTD1/sd-t/OsCCD7* | PN |  |  |  |  |
|  | Os01g0746400 | LOC_Os01g54270 | 1 | 31220321 | 31228566 | *D10/ OsCCD8* | PN |  |  |  |  |
|  | Os07g0282300 | LOC_Os07g18154 | 7 | 10748746 | 10760833 |  |  |  | GNP | PN |  |
|  | Os07g0282401 | LOC_Os07g18158 | 7 | 10762643 | 10763602 |  |  |  | GNP |  |  |
|  | Os02g0817900 | LOC_Os02g57290 | 2 | 35092344 | 35099291 |  |  |  | GNP |  |  |
| Circadian rhythm - plant  (9 cloned genes + 1 candidate gene) | Os01g0182600 | LOC_Os01g08700 | 1 | 4329285 | 4338486 | *OsGI* | HD |  |  |  |  |
|  | Os01g0566100 | LOC_Os01g38530 | 1 | 21639928 | 21643111 | *OsEF3* | HD |  |  |  |  |
|  | Os02g0771100 | LOC_Os02g53140 | 2 | 32528037 | 32533583 | *PPS* | HD |  |  |  |  |
|  | Os03g0309200 | LOC_Os03g19590 | 3 | 11020091 | 11028228 | *PHYB* | HD |  |  |  |  |
|  | Os03g0762000 | LOC_Os03g55389 | 3 | 31508811 | 31514460 | *Hd6; CK2α* | HD |  |  |  |  |
|  | Os06g0142600 | LOC_Os06g05060 | 6 | 2234119 | 2239162 | *Hd17; Ef7; OsELF3; OsELF3-1* | HD |  |  |  |  |
|  | Os06g0157500 | LOC_Os06g06300 | 6 | 2926823 | 2928474 | *RFT1* | HD |  | GNP |  |  |
|  | Os06g0157700 | LOC_Os06g06320 | 6 | 2940004 | 2942452 | *Hd3a* | HD |  | GNP |  |  |
|  | Os07g0695100 | LOC_Os07g49460 | 7 | 29616732 | 29629223 | *DTH7* | HD |  |  |  |  |
|  | Os09g0532400 | LOC_Os09g36220 | 9 | 20885172 | 20889843 |  |  | HD |  |  |  |
| Glycolysis / Gluconeogenesis  (1 cloned gene + 8 candidate genes) | Os01g0328700 | LOC_Os01g22520 | 1 | 12654852 | 12658475 |  |  | HD |  |  |  |
|  | Os01g0866400 | LOC_Os01g64660 | 1 | 37519542 | 37522613 | *MOC2; FBP1* | PN |  |  |  |  |
|  | Os03g0325000 | LOC_Os03g20880 | 3 | 11819962 | 11823440 |  |  |  |  |  | KGW |
|  | Os03g0381000 | LOC_Os03g26430 | 3 | 15100555 | 15102434 |  |  |  | GNP |  |  |
|  | Os05g0156700 | LOC_Os05g06460 | 5 | 3313391 | 3319717 |  |  |  |  |  | KGW |
|  | Os05g0194900 | LOC_Os05g10650 | 5 | 5813265 | 5815545 |  |  |  |  | PN |  |
|  | Os07g0410100 | LOC_Os07g22720 | 7 | 12811612 | 12819404 |  |  |  | GNP | PN |  |
|  | Os09g0327400 | LOC_Os09g15820 | 9 | 9655424 | 9659127 |  |  |  | GNP | PN |  |
|  | Os11g0186200 | LOC_Os11g08300 | 11 | 4375281 | 4380044 |  |  |  |  | PN |  |
| Plant hormone signal transduction  (4 cloned genes + 11 candidate genes) | Os01g0178500 | LOC_Os01g08320 | 1 | 4073676 | 4076438 |  |  |  |  |  | KGW |
|  | Os01g0813100 | LOC_Os01g59760 | 1 | 34565292 | 34568290 |  |  |  | GNP |  |  |
|  | Os02g0787300 | LOC_Os02g54600 | 2 | 33442069 | 33443948 | *OsMKK4; SMG1* | KGW |  |  |  |  |
|  | Os03g0700800 | LOC_Os03g49400 | 3 | 28116620 | 28124217 |  |  |  |  | PN |  |
|  | Os05g0143800 | LOC_Os05g05180 | 5 | 2522903 | 2525214 |  |  |  |  |  | KGW |
|  | Os05g0523300 | LOC_Os05g44810 | 5 | 26052916 | 26055714 |  |  | HD |  |  |  |
|  | Os06g0154500 | LOC_Os06g06090 | 6 | 2806544 | 2813004 | *OsMPK1; OsMAPK6; OsSIPK; OsMPK6; DSG1* | KGW |  | GNP |  |  |
|  | Os06g0528300 | LOC_Os06g33690 | 6 | 19604055 | 19606116 |  |  |  |  |  | KGW |
|  | Os07g0576100 | LOC_Os07g38860 | 7 | 23314482 | 23319154 |  |  | HD |  |  |  |
|  | Os07g0686100 | LOC_Os07g48660 | 7 | 29144895 | 29147038 |  |  |  |  |  | KGW |
|  | Os08g0474500 | LOC_Os08g36970 | 8 | 23384707 | 23385710 |  |  | HD |  |  |  |
|  | Os08g0557700 | LOC_Os08g44350 | 8 | 27904573 | 27909555 | *OsAHP1/OHP1* | PN |  |  |  |  |
|  | Os09g0437400 | LOC_Os09g26610 | 9 | 16143735 | 16144535 |  |  |  |  |  | KGW |
|  | Os09g0567400 | LOC_Os09g39400 | 9 | 22653849 | 22657046 | *OsAHP2/OHP2* | PN |  |  |  |  |
|  | Os10g0362300 | LOC_Os10g21810 | 10 | 11192316 | 11203063 |  |  |  | GNP |  |  |
| Starch and sucrose metabolism  (1 cloned gene + 10 candidate genes) | Os01g0633100 | LOC_Os01g44220 | 1 | 25356683 | 25361949 | *OsAGPL2; OsAPL2; shr1* | KGW |  |  |  |  |
|  | Os01g0813700 | LOC_Os01g59819 | 1 | 34588827 | 34602432 |  |  | HD |  |  |  |
|  | Os02g0753000 | LOC_Os02g51680 | 2 | 31663080 | 31665818 |  |  |  |  | PN |  |
|  | Os05g0365600 | LOC_Os05g30250 | 5 | 17530143 | 17534646 |  |  |  |  |  | KGW |
|  | Os05g0366600 | LOC_Os05g30350 | 5 | 17594979 | 17600510 |  |  |  |  |  | KGW |
|  | Os06g0133000 | LOC_Os06g04200 | 6 | 1766194 | 1770656 |  |  |  |  |  | KGW |
|  | Os06g0160700 | LOC_Os06g06560 | 6 | 3079059 | 3086808 |  |  |  | GNP |  |  |
|  | Os06g0194900 | LOC_Os06g09450 | 6 | 4796286 | 4804246 |  |  |  | GNP |  |  |
|  | Os06g0229800 | LOC_Os06g12450 | 6 | 6748358 | 6753338 |  |  | HD |  |  |  |
|  | Os07g0412100 | LOC_Os07g22930 | 7 | 12916277 | 12920695 |  |  |  | GNP |  |  |
|  | Os10g0465700 | LOC_Os10g32810 | 10 | 17180757 | 17183465 |  |  |  |  |  | KGW |
| Diterpenoid biosynthesis  (1 cloned gene + 4 candidate genes) | Os06g0569900 | LOC_Os06g37330 | 6 | 22048646 | 22057757 |  |  | HD |  |  |  |
|  | Os06g0110000 | LOC_Os06g02019 | 6 | 580665 | 586450 | *D3* | PN |  |  |  |  |
|  | Os01g0209700 | LOC_Os01g11150 | 1 | 5968819 | 5972489 |  |  |  |  | PN |  |
|  | Os02g0570400 | LOC_Os02g36140 | 2 | 21765009 | 21771452 |  |  |  |  | PN |  |
|  | Os04g0178400 | LOC_Os04g09920 | 4 | 5332329 | 5334479 |  |  |  | GNP |  |  |
| Biosynthesis of amino acids  (2 cloned genes + 3 candidate genes) | Os05g0135700 | LOC_Os05g04510 | 5 | 2089056 | 2092066 |  |  | HD |  |  |  |
|  | Os04g0106300 | LOC_Os04g01590 | 4 | 396481 | 400947 |  |  |  |  |  |  |
|  | Os04g0254000 | LOC_Os04g18200 | 4 | 10061764 | 10065209 | *OsARG* | GNP |  | GNP |  |  |
|  | Os04g0659100 | LOC_Os04g56400 | 4 | 33627909 | 33631095 |  |  |  |  |  |  |
|  | Os04g0406600 | LOC_Os04g33390 | 4 | 20169585 | 20171246 | *OsGS2/OsGLN2/λGS31* | PN |  |  | PN |  |
| Plant-pathogen interaction  (1 cloned gene + 4 candidate genes) | Os03g0366200 | LOC_Os03g25070 | 3 | 14316087 | 14323469 |  |  |  |  |  | KGW |
|  | Os07g0409900 | LOC_Os07g22710 | 7 | 12794960 | 12803568 |  |  |  | GNP |  |  |
|  | Os12g0230200 | LOC_Os12g12860 | 12 | 7112286 | 7116204 |  |  |  | GNP |  |  |
|  | Os02g0787300 | LOC_Os02g54600 | 2 | 33442069 | 33443948 |  |  |  |  |  |  |
|  | Os08g0260800 | LOC_Os08g16070 | 8 | 9795796 | 9798581 | *OsMKK4; SMG1* | KGW | HD |  |  |  |
| Porphyrin and chlorophyll metabolism  (2 cloned genes + 1 candidate gene) | Os01g0949400 | LOC_Os01g72090 | 1 | 41822269 | 41825087 | *Se13; OsHY2* | HD |  |  |  |  |
|  | Os06g0603000 | LOC_Os06g40080 | 6 | 23853714 | 23858061 | *Se5; OsHY1; OsHO1* | HD |  |  |  |  |
|  | Os10g0369000 | LOC_Os10g22380 | 10 | 11582003 | 11585288 |  |  |  | GNP |  | KGW |
| Zeatin biosynthesis  (1 cloned gene + 1 candidate gene) | Os01g0197700 | LOC_Os01g10110 | 1 | 5270103 | 5275678 | *Gn1a; OsCKX2* | GNP |  |  |  |  |
|  | Os10g0483500 | LOC_Os10g34230 | 10 | 18270169 | 18274977 |  |  |  |  |  | KGW |
| Ubiquitin mediated proteolysis  (2 cloned genes + 3 candidate genes) | Os03g0123300 | LOC_Os03g03150 | 3 | 1327397 | 1331210 | *TAD1/TE* | PN | HD |  |  |  |
|  | Os02g0771100 | LOC_Os02g53140 | 2 | 32528037 | 32533583 | *PPS* | HD |  |  |  |  |
|  | Os07g0411200 | LOC_Os07g22840 | 7 | 12881692 | 12883433 |  |  |  | GNP | PN |  |
|  | Os07g0409500 | LOC_Os07g22680 | 7 | 12777061 | 12778037 |  |  |  | GNP | PN |  |
|  | Os01g0819400 | LOC_Os01g60360 | 1 | 34917682 | 34919409 |  |  |  | GNP |  |  |
| Nitrogen metabolism  (1 cloned gene + 1 candidate gene) | Os04g0659100 | LOC_Os04g56400 | 4 | 33627909 | 33631095 | *OsGS2/OsGLN2/λGS31* | PN |  |  |  |  |
|  | Os10g0471300 | LOC_Os10g33270 | 10 | 17471599 | 17474854 |  |  |  |  |  | KGW |

HD: heading date; GNP: grain number per plant; PN: panicle number; KGW: kilo-grain weight.
