# Supplementary material for: Genetic Basis Underlying Correlations Among Growth Duration and Yield Traits Revealed by GWAS in Rice (Oryza sativa L.)
Source: Front Plant Sci. 2018 May 22;9:650. doi: 10.3389/fpls.2018.00650 (PMC5972282; doi:10.3389/fpls.2018.00650)
Supplement: Supplementary file 10 [file Table_10.DOCX]

**SUPPLEMENTARY TABLE 10 | Interactive genes participating in various pathways.**

| **Carbon metabolism.** |  | | | 1 | 2 | 3 | 4 | 5 | 6 | 7 | 8 | 9 | 10 | 11 | 12 |
| --- | --- | --- | --- | --- | --- | --- | --- | --- | --- | --- | --- | --- | --- | --- | --- |
|  | LOC_Os01g22520 | HD | 1 | 1 |  |  |  |  |  |  |  |  |  |  |  |
|  | LOC_Os01g64660 | PN | 2 | SY(**) | 1 |  |  |  |  |  |  |  |  |  |  |
|  | LOC_Os02g44550 | GNP | 3 | - | - | 1 |  |  |  |  |  |  |  |  |  |
|  | LOC_Os03g07300 | KGW | 4 | SY(*), CS(**) | - | - | 1 |  |  |  |  |  |  |  |  |
|  | LOC_Os03g08660 | HD | 5 | - | - | SY(**) | - | 1 |  |  |  |  |  |  |  |
|  | LOC_Os04g32020 | PN | 6 | - | - | - | SY(*) | - | 1 |  |  |  |  |  |  |
|  | LOC_Os05g06460 | KGW | 7 | CS_*jap*.(*) | - | - | - | - | - | 1 |  |  |  |  |  |
|  | LOC_Os05g09440 | HD | 8 | - | - | - | - | - | - | - | 1 |  |  |  |  |
|  | LOC_Os05g10650 | PN | 9 | - | - | - | - | - | - | SY(*) | - | 1 |  |  |  |
|  | LOC_Os06g04270 | KGW | 10 | - | - | - | - | - | - | - | - | - | 1 |  |  |
|  | LOC_Os07g22350 | GNP PN | 11 | - | - | - | - | - | - | - | CS(**) | - | - | 1 |  |
|  | LOC_Os11g41160 | PN | 12 | CS(*) | - | - | - | - | - | - | - | - | CS_*jap*.(*) | - | 1 |
| **Carotenoid biosynthesis** |  | |  | 1 | 2 | 3 | 4 | 5 |  |  |  |  |  |  |  |
|  | LOC_Os04g46470 | PN | 1 | 1 |  |  |  |  |  |  |  |  |  |  |  |
|  | LOC_Os01g54270 | PN | 2 | - | 1 |  |  |  |  |  |  |  |  |  |  |
|  | LOC_Os07g18154 | GNP PN | 3 | - | CS(**) | 1 |  |  |  |  |  |  |  |  |  |
|  | LOC_Os07g18158 | GNP | 4 | - | - | SY_*ind*.(**), CS(**) | 1 |  |  |  |  |  |  |  |  |
|  | LOC_Os02g57290 | GNP | 5 | SY(*) | - | - | - | 1 |  |  |  |  |  |  |  |
| **Plant hormone Signal transduction.** |  | |  | 1 | 2 | 3 | 4 | 5 | 6 | 7 |  |  |  |  |  |
|  | LOC_Os01g59760 | GNP | 1 | 1 |  |  |  |  |  |  |  |  |  |  |  |
|  | LOC_Os05g05180 | KGW | 2 | - | 1 |  |  |  |  |  |  |  |  |  |  |
|  | LOC_Os05g44810 | HD | 3 | - | - | 1 |  |  |  |  |  |  |  |  |  |
|  | LOC_Os06g06090 | KGW GNP | 4 | - | - | - | 1 |  |  |  |  |  |  |  |  |
|  | LOC_Os07g48660 | KGW | 5 | SY(**) | - | CS_*jap*.(**), SY(*) | - | 1 |  |  |  |  |  |  |  |
|  | LOC_Os08g36970 | HD | 6 | CS_*ind*.(*) | - | - | - | - | 1 |  |  |  |  |  |  |
|  | LOC_Os08g44350 | PN | 7 |  | CS(**) | - | CS(*) | SY(**), CS(**) | - | 1 |  |  |  |  |  |
| **Circadian rhythm - plant** |  | |  | 1 | 2 | 3 | 4 |  |  |  |  |  |  |  |  |
|  | LOC_Os02g53140 | HD | 1 | 1 |  |  |  |  |  |  |  |  |  |  |  |
|  | LOC_Os03g55389 | HD | 2 | - | 1 |  |  |  |  |  |  |  |  |  |  |
|  | LOC_Os06g06300 | HDGNP | 3 | - | SY(**) | 1 |  |  |  |  |  |  |  |  |  |
|  | LOC_Os06g06320 | HDGNP | 4 | SY(**) | SY(**) | - | 1 |  |  |  |  |  |  |  |  |
| **Glycolysis / Gluconeogenesis** |  | |  | 1 | 2 | 3 | 4 | 5 | 6 | 7 | 8 |  |  |  |  |
|  | LOC_Os05g06460 | KGW | 1 | 1 |  |  |  |  |  |  |  |  |  |  |  |
|  | LOC_Os01g64660 | PN | 2 | SY_*ind*.(**), SY(**) | 1 |  |  |  |  |  |  |  |  |  |  |
|  | LOC_Os11g08300 | PN | 3 | - |  | 1 |  |  |  |  |  |  |  |  |  |
|  | LOC_Os07g22720 | GNPPN | 4 | - | CS(**) | - | 1 |  |  |  |  |  |  |  |  |
|  | LOC_Os05g10650 | PN | 5 | SY(**) | - | - | - | 1 |  |  |  |  |  |  |  |
|  | LOC_Os03g20880 | KGW | 6 | - | - | - | CS(**) | - | 1 |  |  |  |  |  |  |
|  | LOC_Os09g15820 | GNPPN | 7 | - | - | SY_*ind*(*) | - | - | - | 1 |  |  |  |  |  |
|  | LOC_Os03g26430 | GNP | 8 | SY_*jap*.(**) | - | - | - | - | - | - | 1 |  |  |  |  |
| **Ubiquitin mediated proteolysis** |  | |  | 1 | 2 | 3 | 4 |  |  |  |  |  |  |  |  |
|  | LOC_Os01g60360 | GNP | 1 | 1 |  |  |  |  |  |  |  |  |  |  |  |
|  | LOC_Os02g53140 | HD | 2 | - | 1 |  |  |  |  |  |  |  |  |  |  |
|  | LOC_Os07g22680 | GNPPN | 3 | SY(**), SY_*ind*.(**) | - | 1 |  |  |  |  |  |  |  |  |  |
|  | LOC_Os07g22840 | GNPPN | 4 | - | SY(**) | - | 1 |  |  |  |  |  |  |  |  |
| **Arginine and proline metabolism** |  | |  | 1 | 2 | 3 | 4 |  |  |  |  |  |  |  |  |
|  | LOC_Os04g01590 | GNP | 1 | 1 |  |  |  |  |  |  |  |  |  |  |  |
|  | LOC_Os04g10410 | GNP | 2 | - | 1 |  |  |  |  |  |  |  |  |  |  |
|  | LOC_Os04g10569 | HDGNP | 3 | - | SY(**) | 1 |  |  |  |  |  |  |  |  |  |
|  | LOC_Os06g04070 | KGW | 4 | CS(**) | - | - | 1 |  |  |  |  |  |  |  |  |
| **Starch and sucrose metabolism** |  | |  | 1 | 2 | 3 | 4 | 5 | 6 | 7 | 8 |  |  |  |  |
|  | LOC_Os01g59819 | HD | 1 | 1 |  |  |  |  |  |  |  |  |  |  |  |
|  | LOC_Os02g51680 | PN | 2 | - | 1 |  |  |  |  |  |  |  |  |  |  |
|  | LOC_Os05g30350 | KGW | 3 | SY_*ind*(*) | - | 1 |  |  |  |  |  |  |  |  |  |
|  | LOC_Os06g06560 | GNP | 4 | CS(**) | - | - | 1 |  |  |  |  |  |  |  |  |
|  | LOC_Os06g09450 | GNP | 5 | CS_*ind*(*) | - | SY(**) |  | 1 |  |  |  |  |  |  |  |
|  | LOC_Os06g12450 | HD | 6 | - | - | - | CS_*ind*(**), CS(**) | - | 1 |  |  |  |  |  |  |
|  | LOC_Os07g22930 | GNP | 7 | SY_*ind*(**) | SY_*ind*(**), SY(**) | - | - | - | - | 1 |  |  |  |  |  |
|  | LOC_Os10g32810 | KGW | 8 | - | CS_*ind*(*) | - | - | - | - | CS_*jap*(**) | 1 |  |  |  |  |

"*": significant correlation at the *P* = 0.05;"**": significant correlation at the *P* = 0.01; SY: Sanya; CS: Changsha; *Ind.*: *indica*; *Jap*.:*japonica*; HD: heading date; GNP: grain number per plant; PN: panicle number; KGW: kilo-grain weight.
