# Supplementary material for: Genetic Basis Underlying Correlations Among Growth Duration and Yield Traits Revealed by GWAS in Rice (Oryza sativa L.)
Source: Front Plant Sci. 2018 May 22;9:650. doi: 10.3389/fpls.2018.00650 (PMC5972282; doi:10.3389/fpls.2018.00650)
Supplement: Supplementary file 11 [file Table_11.DOCX]

**SUPPLEMENTARY TABLE 11 | Reported cloned genes for heading date.**

| **Class** | **Gene for HD (38)** | **ID** | | **Position** | | | **Trait** | | **Function** |
| --- | --- | --- | --- | --- | --- | --- | --- | --- | --- |
| Photoperiod-  sensitivity (PS) | *OsMADS51 OsMADS65* | Os01g0922800 | LOC_Os01g69850 | 1 | 40,344,374 | 40,364,362 | HD | MADS-Box gene | |
|  | *Se13; OsHY2* | Os01g0949400 | LOC_Os01g72090 | 1 | 41,822,269 | 41,825,087 | HD | Photoperiod-sensitivity-13 | |
|  | *LC2; OsVIL3* | Os02g0152500 | LOC_Os02g05840 | 2 | 2,876,559 | 2,882,177 | secondary effect-HD | Rice leaf inclination2 | |
|  | *OsCOL4* | Os02g0610500 | LOC_Os02g39710 | 2 | 23,989,803 | 23,991,271 | HD | Flowering repressor | |
|  | *OsDof12* | Os03g0169600 | LOC_Os03g07360 | 3 | 3,738,868 | 3,741,611 | secondary effect-HD | DNA-binding with one finger protein | |
|  | *Ehd4* | Os03g0112700 | LOC_Os03g02160 | 3 | 717,839 | 720,374 | HD | Early heading date 4 | |
|  | *PHYB* | Os03g0309200 | LOC_Os03g19590 | 3 | 11,020,152 | 11,028,185 | secondary effect-HD | Phytochrome B | |
|  | *OsMADS14* | Os03g0752800 | LOC_Os03g54160 | 3 | 31,034,529 | 31,041,563 | HD | MADS-box gene | |
|  | *Hd6; CK2α* | Os03g0762000 | LOC_Os03g55389 | 3 | 31,508,813 | 31,514,460 | HD | Heading date-6 | |
|  | *CKI; EL1; Hd16* | Os03g0793500 | LOC_Os03g57940 | 3 | 32,999,502 | 33,006,898 | HD | Heading date 16 | |
|  | *OsLF* | Os05g0541400 | LOC_Os05g46370 | 5 | 26,879,818 | 26,881,142 | HD | Atypical HLH protein | |
|  | *Hd17; Ef7* | Os06g0142600 | LOC_Os06g05060 | 6 | 2,234,156 | 2,236,760 | HD | Heading date 17 | |
|  | *RFT1* | Os06g0157500 | LOC_Os06g06300 | 6 | 2,926,823 | 2,928,474 | secondary effect-HD | Florigen gene | |
|  | *Hd3a* | Os06g0157700 | LOC_Os06g06320 | 6 | 2,940,004 | 2,942,452 | HD | Heading date-3a | |
|  | *Se5; OsHY1; OsHO1* | Os06g0603000 | LOC_Os06g40080 | 6 | 23,853,783 | 23,858,023 | HD | Photoperiod-sensitivity-5 | |
|  | *Ghd7* | Os07g0261200 | LOC_Os07g15770 | 7 | 9,152,377 | 9,155,030 | secondary effect-HD | Grains.Height.Date-7 | |
|  | *DTH7* | Os07g0695100 | LOC_Os07g49460 | 7 | 29,616,705 | 29,629,215 | HD | Days to heading 7 | |
|  | *DTH8; Ghd8* | Os08g0174500 | LOC_Os08g07740 | 8 | 4,332,106 | 4,334,829 | secondary effect-HD | Grain yield, heading date, plant height | |
|  | *OsFCA* | Os09g0123200 | LOC_Os09g03610 | 9 | 1,790,628 | 1,799,552 | HD | Heading dates in rice | |
|  | *OsCO3* | Os09g0240200 | LOC_Os09g06464 | 9 | 3,048,085 | 3,064,471 | secondary effect-HD | Constans-like gene | |
|  | *Ehd1* | Os10g0463400 | LOC_Os10g32600 | 10 | 17,076,098 | 17,081,344 | HD | Early heading date 1 | |
|  | *OsFKF1* | Os11g0547000 | LOC_Os11g34460 | 11 | 20,182,477 | 20,187,327 | HD | Flavin-binding, kelch repeat, F-box 1 | |
|  | *OsVIL2* | Os12g0533500 | LOC_Os12g34850 | 12 | 21,210,477 | 21,214,801 | secondary effect-HD | Vernalization insensitive 2-like protein | |
| Photoperiod-  sensitivity (PS)/temperature sensitivity (TS) | *OsGI* | Os01g0182600 | LOC_Os01g08700 | 1 | 4,329,362 | 4,338,486 | secondary effect-HD | Gigantea | |
|  | *DTH2* | Os02g0724000 | LOC_Os02g49230 | 2 | 30,094,300 | 30,099,072 | HD | Days to heading; constans-like protein | |
|  | *OsMADS50* | Os03g0122600 | LOC_Os03g03070 | 3 | 1,270,320 | 1,300,273 | HD | MADS-box protein gene | |
|  | *Hd1* | Os06g0275000 | LOC_Os06g16370 | 6 | 9,336,376 | 9,338,569 | HD | Heading date 1 | |
|  | *OsMADS56* | Os10g0536100 | LOC_Os10g39130 | 10 | 20,863,187 | 20,873,635 | secondary effect-HD | MADS-box protein gene | |
| Basic vegetative growth (BVG) | *OsEF3* | Os01g0566100 | LOC_Os01g38530 | 1 | 21,639,932 | 21,643,059 | HD | Early flowering gene | |
|  | *SDG725* | Os02g0554000 | LOC_Os02g34850 | 2 | 20,900,191 | 20,910,475 | HD | H3K36 methyltransferase | |
|  | *PPS* | Os02g0771100 | LOC_Os02g53140 | 2 | 32,528,080 | 32,533,583 | secondary effect-HD | Peter pan syndrome | |
|  | *NRR;OsmiR393* | Os05g0595300 | LOC_Os05g51690 | 5 | 29,656,681 | 29,660,315 | secondary effect-HD | Nutrition response and root growth,microRNA | |
|  | *Ehd3* | Os08g0105000 | LOC_Os08g01420 | 8 | 272,854 | 276,978 | HD | Early heading date 3 | |
|  | *Ehd2; RID1* | Os10g0419200 | LOC_Os10g28330 | 10 | 14,739,603 | 14,743,222 | HD | Transcription factor for encoding Cys2  /His2 zinc finger protein | |
| Other | *AID1* | Os06g0181300 | LOC_Os06g08290 | 6 | 4,014,963 | 4,018,328 | secondary effect-HD | Anther indehiscence1 | |
|  | *RFL; APO2* | Os04g0598300 | LOC_Os04g51000 | 4 | 30,182,589 | 30,185,852 | HD | Aberrant panicle organization 2 | |
|  | *HGW* | Os06g0160400 | LOC_Os06g06530 | 6 | 3,060,007 | 3,063,606 | HD | Heading and grain weight | |
|  | *OsLFL1* | Os01g0713600 | LOC_Os01g51610 | 1 | 29,638,075 | 29,642,862 | HD | Transcription factor | |

HD: heading date.
