# Supplementary material for: Genetic Basis Underlying Correlations Among Growth Duration and Yield Traits Revealed by GWAS in Rice (Oryza sativa L.)
Source: Front Plant Sci. 2018 May 22;9:650. doi: 10.3389/fpls.2018.00650 (PMC5972282; doi:10.3389/fpls.2018.00650)
Supplement: Supplementary file 12 [file Table_12.DOCX]

**SUPPLEMENTARY TABLE 12 | Reported cloned genes for grain number per panicle.**

| **Participation** | | **Gene for GNP (28)** | **ID** | | **Position** | | | **Trait** | **Annotation** |
| --- | --- | --- | --- | --- | --- | --- | --- | --- | --- |
| Erect panicle | CTK | *EP3; LP* | Os02g0260200 | LOC_Os02g15950 | 2 | 9,042,134 | 9,044,852 | GNP | Erect panicle 3 |
|  | GA | *DEP3* | Os06g0677000 | LOC_Os06g46350 | 6 | 28,109,236 | 28,111,005 | GNP | Dense and erect panicle3 |
|  | - | *DEP2; EP2* | Os07g0616000 | LOC_Os07g42410 | 7 | 25,381,698 | 25,389,547 | GNP | Dense and erect panicle2 |
|  | N | *DEP1; DN1* | Os09g0441900 | LOC_Os09g26999 | 9 | 16,411,151 | 16,415,851 | GNP | Dense and erect panicle1 |
| Panicle length | CTK | *LOG* | Os01g0588900 | LOC_Os01g40630 | 1 | 22,955,070 | 22,959,148 | secondary effect-GNP | Lonely guy |
|  | ABA,JA | *Ghd7* | Os07g0261200 | LOC_Os07g15770 | 7 | 9,152,377 | 9,155,030 | GNP | Grains.height.date-7 |
|  | - | *sp1* | Os11g0235200 | LOC_Os11g12740 | 11 | 7,193,230 | 7,198,552 | GNP | Short panicle1 |
|  | Cellulose and xylose | *OsCD1* | Os12g0555600 | LOC_Os12g36890 | 12 | 22,602,934 | 22,607,307 | secondary effect-GNP | Cellulose synthase-like D sub-family |
| Branch | - | *LAX1* | Os01g0831000 | LOC_Os01g61480 | 1 | 37,287,830 | 37,288,907 | GNP | Lax panicle |
|  | - | *LAX2* | Os04g0396500 | LOC_Os04g32510 | 4 | 19,564,239 | 19,566,320 | GNP | Lax panicle2 |
|  | IAA | *APO2;RFL* | Os04g0598300 | LOC_Os04g51000 | 4 | 30,182,589 | 30,185,852 | GNP | Aberrant panicle organizaion 2 |
|  | - | *APO1* | Os06g0665400 | LOC_Os06g45460 | 6 | 28,357,085 | 28,358,453 | GNP | Aberrant panicle organization 1 |
|  | IAA | *OsLIS-L1* | Os08g0162100 | LOC_Os08g06480 | 8 | 3,667,092 | 3,675,813 | GNP | Aberrant spikelet and panicle1 |
|  | - | *DTH8; Ghd8* | Os08g0174500 | LOC_Os08g07740 | 8 | 4,332,106 | 4,334,829 | secondary effect-GNP | Grain yield, heading date, plant height |
|  | - | *OsSPL14* | Os08g0509600 | LOC_Os08g39890 | 8 | 25,274,541 | 25,278,696 | GNP | Squamosa promoter binding protein-like 14 |
| Spikelet development | CTK | *Gn1a* | Os01g0197700 | LOC_Os01g10110 | 1 | 5,270,449 | 5,275,585 | GNP | Grain number |
|  | - | *OsIDS1* | Os03g0818800 | LOC_Os03g60430 | 3 | 34,358,193 | 34,362,334 | GNP | Indeterminate spikelet 1 |
|  | - | *MFS1* | Os05g0497200 | LOC_Os05g41760 | 5 | 24,427,885 | 24,428,869 | GNP | Multi-floret spikelet1 |
|  | JA | *OsJMT1* | Os06g0314600 | LOC_Os06g20920 | 6 | 12,082,449 | 12,088,593 | GNP | Jasmonic acid carboxyl methyltransferase gene |
|  | GA | *sped1-D* | Os06g0597500 | LOC_Os06g39650 | 6 | 23,548,029 | 23,549,621 | GNP | Pentatricopeptide repeat protein |
|  | - | *FZP* | Os07g0669500 | LOC_Os07g47330 | 7 | 28,299,602 | 28,301,089 | GNP | Frizzy panicle |
|  | ABA | *OsSDR* | Os07g0685800 | LOC_Os07g48640 | 7 | 29,121,890 | 29,124,939 | secondary effect-GNP | Short-chain alcohol dehydrogenase |
|  | - | *TAW1* | Os10g0478000 | LOC_Os10g33780 | 10 | 17,888,297 | 17,889,724 | GNP | Tawawa1 |
| Setting percentage | - | *DFO1; CCP1* | Os01g0229300 | LOC_Os01g12890 | 1 | 7,157,780 | 7,163,626 | GNP | Deformed floral organ 1 |
|  | CTK | *OsMADS29* | Os02g0170300 | LOC_Os02g07430 | 2 | 3,833,130 | 3,837,135 | GNP | MADS-box gene |
|  | N | *OsARG* | Os04g0106300 | LOC_Os04g01590 | 4 | 396,563 | 400,802 | secondary effect-GNP | Arginase |
|  | - | *GSD1* | Os04g0620200 | LOC_Os04g52920 | 4 | 31,526,357 | 31,527,861 | GNP | Grain setting defect1;Remorin Protein |
|  | - | *PTB1* | Os05g0145000 | LOC_Os05g05280 | 5 | 2,598,549 | 2,604,164 | GNP | Pollen tube blocked |

GNP: grain number per plant.
