# Supplementary material for: Genetic Basis Underlying Correlations Among Growth Duration and Yield Traits Revealed by GWAS in Rice (Oryza sativa L.)
Source: Front Plant Sci. 2018 May 22;9:650. doi: 10.3389/fpls.2018.00650 (PMC5972282; doi:10.3389/fpls.2018.00650)
Supplement: Supplementary file 13 [file Table_13.DOCX]

**SUPPLEMENTARY TABLE 13 | Reported cloned genes for panicle number / tiller number.**

| **Participation** | | **Gene for TN/PN (31)** | **ID** | | **Position** | | | | **Trait** | **Annotation** |
| --- | --- | --- | --- | --- | --- | --- | --- | --- | --- | --- |
| Hormone | ABA/GA | *OsAP2-39* | Os04g0610400 | LOC_Os04g52090 | 4 | 30,939,170 | 30,940,160 | | TN | Apetala-2-like transcription factor gene |
|  | CTK | *MOC3; OsWUS* | Os04g0663600 | LOC_Os04g56780 | 4 | 33,860,374 | 33,861,424 | | TN | Monoculm 3 |
|  |  | *OsMT2b* | Os05g0111300 | LOC_Os05g02070 | 5 | 605,868 | 606,764 | | TN | Metallothionein gene |
|  |  | *OsAHP1;OHP1* | Os08g0557700 | LOC_Os08g44350 | 8 | 27,992,607 | 27,997,515 | | TN | Histidine Phosphotransfer Protein |
|  |  | *OsAHP2;OHP2* | Os09g0567400 | LOC_Os09g39400 | 9 | 23,538,005 | 23,541,047 | | TN | Histidine Phosphotransfer Protein |
|  | IAA | *D62;DLT* | Os06g0127800 | LOC_Os06g03710 | 6 | 1,465,500 | 1,468,583 | | TN | Dwarf and low tillering |
|  |  | *moc1* | Os06g0610300 | LOC_Os06g40780 | 6 | 24,312,470 | 24,313,095 | | TN/PN | Monoculm 1 |
|  |  | *TAD1;TE* | Os03g0123300 | LOC_Os03g03150 | 3 | 1,327,450 | 1,331,022 | | TN | Tillering and Dwarf 1 |
|  |  | *MIP1* | Os04g0550400 | LOC_Os04g46450 | 4 | 27,551,423 | 27,559,580 | | TN | MOC1 interacting protein 1 |
|  |  | *OsIAGLU* | Os03g0693600 | LOC_Os03g48740 | 3 | 27,775,328 | 27,778,427 | | TN/PN | IAA-glucose synthase |
|  |  | *OsPIN2* | Os06g0660200 | LOC_Os06g44970 | 6 | 27,200,808 | 27,204,611 | | TN | Auxin efflux transporter |
|  | SL | *D17* |  |  |  |  |  |  |  |  |
|  |  | *LAX1* | Os01g0831000 | LOC_Os01g61480 | 1 | 37,287,830 | | 37,288,907 | TN | Regulator of axillary meristem formation |
|  |  | *OsMADS57* | Os02g0731200 | LOC_Os02g49840 | 2 | 30,456,666 | | 30,462,759 | TN | MADS-box gene |
|  |  | *htd2;D88;D14* | Os03g0203200 | LOC_Os03g10620 | 3 | 5,422,206 | | 5,423,780 | TN | High tillering dwarf 2 |
|  |  | *HTD1;OsCCD7* | Os04g0550600 | LOC_Os04g46470 | 4 | 27,382,685 | | 27,385,787 | TN | Dwarf and increased tillering1 |
|  |  | *D3* | Os06g0110000 | LOC_Os06g02019 | 6 | 580,670 | | 586443 | TN | Bungetsuwaito tillering dwarf |
|  |  | *d53* | Os11g0104300 | LOC_Os11g01330 | 11 | 194,178 | | 196,047 | TN | Dwarf 53 |
|  |  | *d27* | Os11g0587000 | - | 11 | 22,221,628 | | 22,231,042 | TN | Dwarf 27 |
|  |  | *SPL14;IPA1* | Os08g0509600 | LOC_Os08g39890 | 8 | 25,274,541 | | 25,278,696 | TN | Squamosa promoter binding protein-like 14 |
|  |  | *D10;OsCCD8* | Os01g0746400 | LOC_Os01g54270 | 1 | 31,225,458 | | 31,228,566 | TN | Carotenoid cleavage dioxygenase 8 |
|  |  | *TB1;FC1* | Os03g0706500 | LOC_Os03g49880 | 3 | 28,428,504 | | 28,430,438 | TN | Rice teosinte branched 1 |
| Mitochondria electron transport chain | | *OGR1* | Os12g0270200 | LOC_Os12g17080 | 12 | 9,777,776 | | 9,782,458 | TN | Pentatricopeptide repeat–DYW protein |
| Pleiotropy | | *OsJAG* | Os01g0129200 | LOC_Os01g03840 | 1 | 1,625,159 | | 1,626,574 | TN | Complete-deletion mutant |
|  |  | *TUT1;ES1* | Os01g0208600 | LOC_Os01g11040 | 1 | 5,890,605 | | 5,898,224 | TN | Suppressor of cAMP receptor-like protein |
|  |  | *RFL;APO2* | Os04g0598300 | LOC_Os04g51000 | 4 | 30,182,589 | | 30,185,852 | secondary effect-TN | Aberrant panicle organization 2 |
|  |  | *AID1* | Os06g0181300 | LOC_Os06g08290 | 6 | 4,014,963 | | 4,018,328 | TN/PN | Anther indehiscence1 |
|  |  | *OsTEF1* | Os02g0134300 | LOC_Os02g04160 | 2 | 1,816,334 | | 1,819,090 | TN | Transcription elongation factor |
|  |  | *PROG1* | Os07g0153600 | LOC_Os07g05900 | 7 | 2,838,477 | | 2,838,980 | secondary effect-PN | Prostrate growth 1 |
| Organic N | | *OsGS2* | Os04g0659100 | LOC_Os04g56400 | 4 | 33,624,428 | | 33,631,025 | TN | Glutamine synthetase |
| Sucrose | | *MOC2;FBP1* | Os01g0866400 | LOC_Os01g64660 | 1 | 37,519,585 | | 37,522,414 | TN | Monoculm 2 |

TN: tiller number; PN: panicle number.
