# Supplementary material for: Genetic Basis Underlying Correlations Among Growth Duration and Yield Traits Revealed by GWAS in Rice (Oryza sativa L.)
Source: Front Plant Sci. 2018 May 22;9:650. doi: 10.3389/fpls.2018.00650 (PMC5972282; doi:10.3389/fpls.2018.00650)
Supplement: Supplementary file 14 [file Table_14.DOCX]

**SUPPLEMENTARY TABLE 14 | Reported cloned genes for kilo-grain weight.**

| **Participation** | **Gene for KGW (25)** | **ID** | | **Position** | | | **Trait** | **Annotation** |
| --- | --- | --- | --- | --- | --- | --- | --- | --- |
| BR | *OsMPK1* | Os06g0154500 | LOC_Os06g06090 | 6 | 2,813,004 | 2,806,543 | GL, GW, KGW | Nitogen activated protein gene |
|  | *OsBZR* | Os07g0580500 | LOC_Os07g39220 | 7 | 23,485,344 | 23,483,809 | GL, GW, GT, KGW | BR-signaling factor |
| CTK/N | *OsCEP6.1* | Os08g0475500 | LOC_Os08g37070 | 8 | 23,425,536 | 23,425,856 | GL, GW, KGW | C-terminally encoded peptide gene |
| E3/GIFI | *GW2* | Os02g0244100 | LOC_Os02g14720 | 2 | 8,115,223 | 8,121,651 | GW, KGW | Major QTL associated with rice grain width and weight |
|  | *gw5* | - | - | 5 | 5,727,083 | 5,727,339 | GW, KGW | Major QTL associated with rice grain width and weight |
|  | *HGW* | Os06g0160400 | LOC_Os06g06530 | 6 | 3,060,007 | 3,063,606 | KGW | Heading and grain weight |
|  | *GS3* | Os03g0407400 | - | 3 | 16,729,501 | 16,735,109 | KGW, GL | Major QTL for grain length and weight |
| GA | *OsOFP2* | Os01g0625900 | LOC_Os01g43610 | 1 | 24,982,666 | 24,981,173 | GW, L/W | Ovate Family Protein 2 |
| IAA | *BG1* | Os03g0175800 | LOC_Os03g07920 | 3 | 4,039,509 | 4,037,983 | GL, GW | Big Grain1 |
|  | *TGW6* | Os06g0623700 | LOC_Os06g41850 | 6 | 25,093,242 | 25,094,294 | KGW | Thousand-grain weight 6 |
| Plasmodesmata | *GSD1; gsd1-D* | Os04g0620200 | LOC_Os04g52920 | 4 | 31,526,357 | 31,527,861 | KGW, GW | Grain setting defect 1 |
| Starch | *flo2* | Os04g0645100 | LOC_Os04g55230 | 4 | 32,835,169 | 32,848,481 | GS | Floury endosperm 2 |
|  | *OsACS6; SSG6* | Os06g0130400 | LOC_Os06g03990 | 6 | 1,629,717 | 1,633,629 | GW, GL, KGW | Substandard starch grain6 |
|  | *FLO7* | Os10g0463800 | LOC_Os10g32680 | 10 | 17,115,452 | 17,112,003 | KGW, GT | Floury endosperm7 |
| Cell cycle | *FUWA* | Os02g0234200 | LOC_Os02g13950 | 2 | 7,594,952 | 7,599,871 | GW, GT, GL, KGW | NHL domain-containing protein |
| ABA | *OsAGPL2; OsAPL2* | Os01g0633100 | LOC_Os01g44220 | 1 | 25,353,805 | 25,361,883 | endosperm | Large subunit of ADP-glucose pyrophosphorylase |
|  | *GS5* | Os05g0158500 | LOC_Os05g06660 | 5 | 3,443,769 | 3,439,259 | GS | Regulator of grain size |
| Others | *OsMKK4; SMG1* | Os02g0787300 | LOC_Os02g54600 | 2 | 33,443,948 | 33,442,069 | KGW | Small grain 1 |
|  | *OsPPKL2* | Os05g0144400 | LOC_Os05g05240 | 5 | 2,564,221 | 2,573,089 | GL | Protein phosphatase with kelch-like repeat domain |
|  | *OsABC1-7; OsAGSW1* | Os05g0323800 | LOC_Os05g25840 | 5 | 15,040,797 | 15,044,759 | GW, GL | ABC1-like kinase gene |
|  | *GW6a; OsglHAT1* | Os06g0650300 | LOC_Os06g44100 | 6 | 26,591,904 | 26,593,469 | KGW | Grain weight |
|  | *OsGRF4; GS2; GL2* | Os02g0701300 | LOC_Os02g47280 | 2 | 28,863,274 | 28,866,997 | GS | Grain size on chromosome 2 |
|  | *GE; CYP78A13; BG2* | Os07g0603700 | LOC_Os07g41240 | 7 | 24,713,778 | 24,715,813 | GL, GW, GT | Big grain 2 |
|  | *GL7; GW7* | Os07g0603300 | LOC_Os07g41200 | 7 | 24,664,328 | 24,669,321 | GL, GW, L/W | Grain length on chromosome 7 |
|  | *qGW8; OsSPL16* | Os08g0531600 | LOC_Os08g41940 | 8 | 26,501,167 | 26,506,218 | GW | Squamosa promoter binding protein-like 16 |

KGW: kilo-grain weight; GL: grain length; GW: grain width; GT: grain thickness; L/W: ratio of length and width; GS: grain size.
