# Supplementary material for: Genetic Basis Underlying Correlations Among Growth Duration and Yield Traits Revealed by GWAS in Rice (Oryza sativa L.)
Source: Front Plant Sci. 2018 May 22;9:650. doi: 10.3389/fpls.2018.00650 (PMC5972282; doi:10.3389/fpls.2018.00650)
Supplement: Supplementary file 15 [file Image_1.pdf]

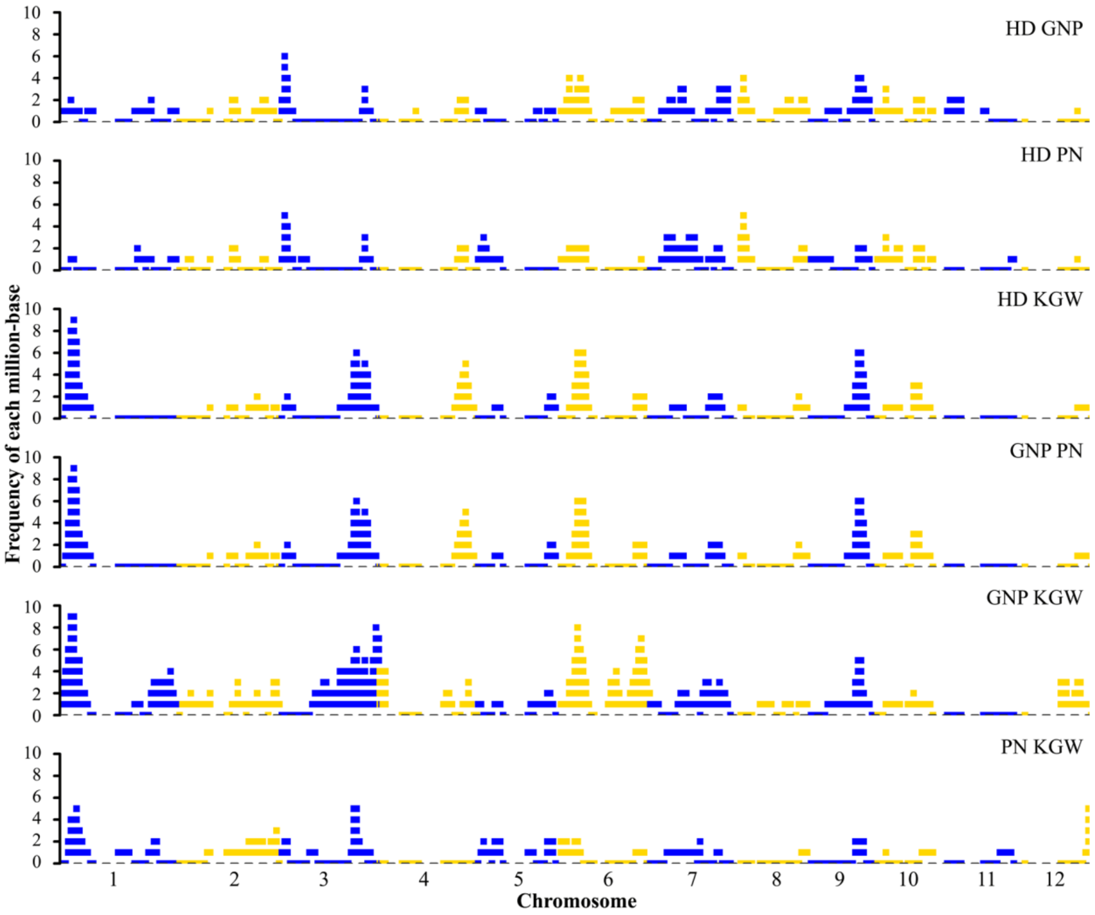

SUPPLEMENTARY FIGURE 1. **Schematics of frequency for each bin in pleiotropic quantitative trait loci (QTLs) from linkage analysis between heading date (HD) and grain number per plant (GNP), HD and panicle number (PN), HD and kilo-grain weight (KGW), GNP and PN, GNP and PN, PN and KGW.** The X axis depicts the physical location across the 12 chromosomes and the Y axis depicts the frequency.
