# Supplementary material for: Genetic Basis Underlying Correlations Among Growth Duration and Yield Traits Revealed by GWAS in Rice (Oryza sativa L.)
Source: Front Plant Sci. 2018 May 22;9:650. doi: 10.3389/fpls.2018.00650 (PMC5972282; doi:10.3389/fpls.2018.00650)
Supplement: Supplementary file 16 [file Image_2.pdf]

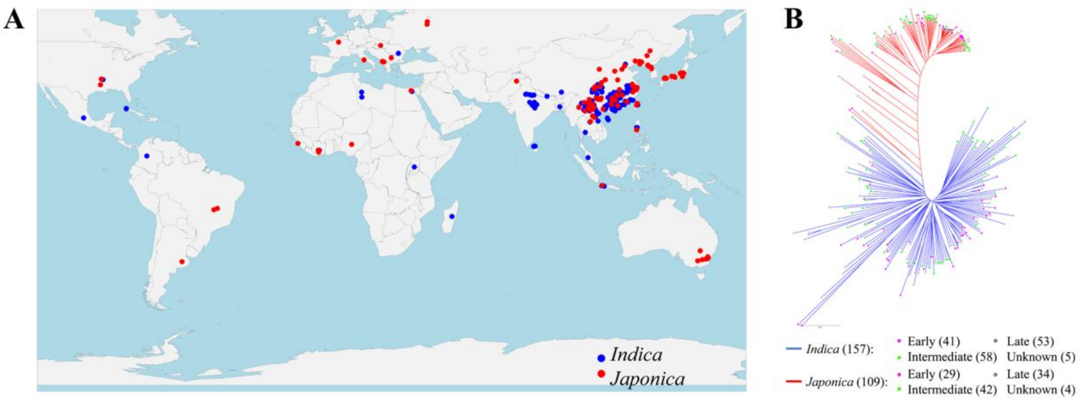


**SUPPLEMENTARY FIGURE 2. Geographic distribution (A) and phylogenetic tree (B) of the mini core collection of 266 cultivated rice varieties used for genome-wide association study (GWAS).** Blue and red dots on the map represent *indica* and *japonica* varieties respectively. On the phylogenetic tree, blue and red lines represent *indica* and *japonica* varieties respectively; light green, pink and black dots represent varieties with early, late, and intermediate maturation dates, respectively.
