# Supplementary material for: Genetic Basis Underlying Correlations Among Growth Duration and Yield Traits Revealed by GWAS in Rice (Oryza sativa L.)
Source: Front Plant Sci. 2018 May 22;9:650. doi: 10.3389/fpls.2018.00650 (PMC5972282; doi:10.3389/fpls.2018.00650)
Supplement: Supplementary file 17 [file Image_3.pdf]

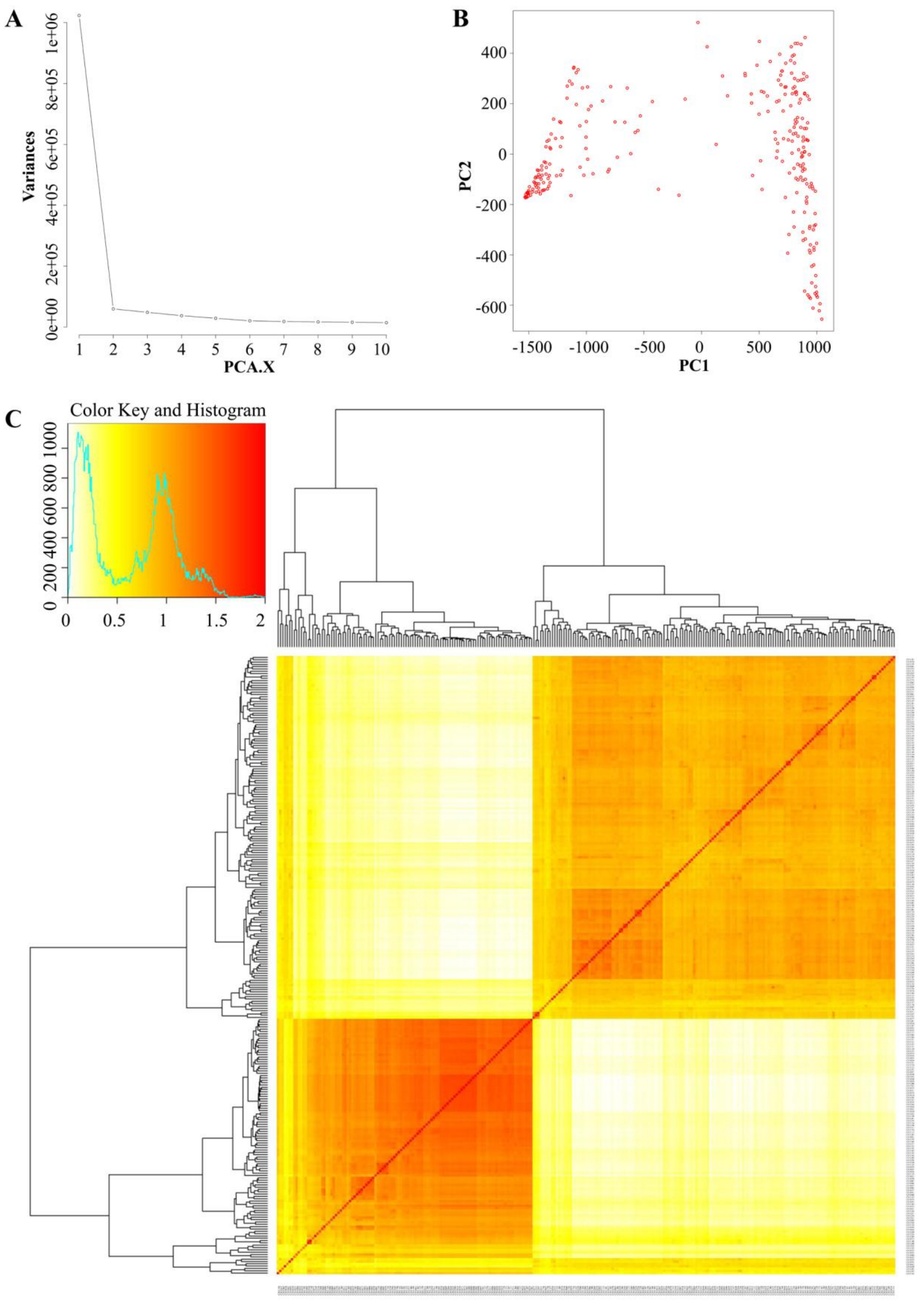

SUPPLEMENTARY FIGURE 3. Variance distribution from principal component analysis from PC1 to PC10 (A), population structure for 266 varieties revealed by PC1 vs PC2 (B) and kinship among individuals (C).
