# Supplementary material for: Genetic Basis Underlying Correlations Among Growth Duration and Yield Traits Revealed by GWAS in Rice (Oryza sativa L.)
Source: Front Plant Sci. 2018 May 22;9:650. doi: 10.3389/fpls.2018.00650 (PMC5972282; doi:10.3389/fpls.2018.00650)
Supplement: Supplementary file 18 [file Image_4.pdf]

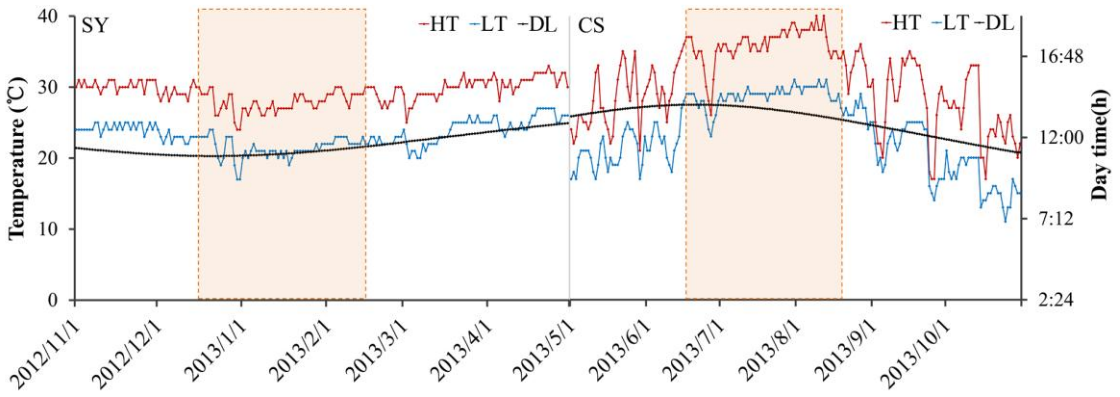


**SUPPLEMENTARY FIGURE 4. Low temperature (LT), high temperature (HT) and day length (DL) during the growing seasons in which the mini core collection was grown at Sanya (SY, left) and Changsha (CS, right).** The periods from transplanting to beginning of heading are shadowed.
