# Supplementary material for: Genetic Basis Underlying Correlations Among Growth Duration and Yield Traits Revealed by GWAS in Rice (Oryza sativa L.)
Source: Front Plant Sci. 2018 May 22;9:650. doi: 10.3389/fpls.2018.00650 (PMC5972282; doi:10.3389/fpls.2018.00650)
Supplement: Supplementary file 19 [file Image_5.PDF]

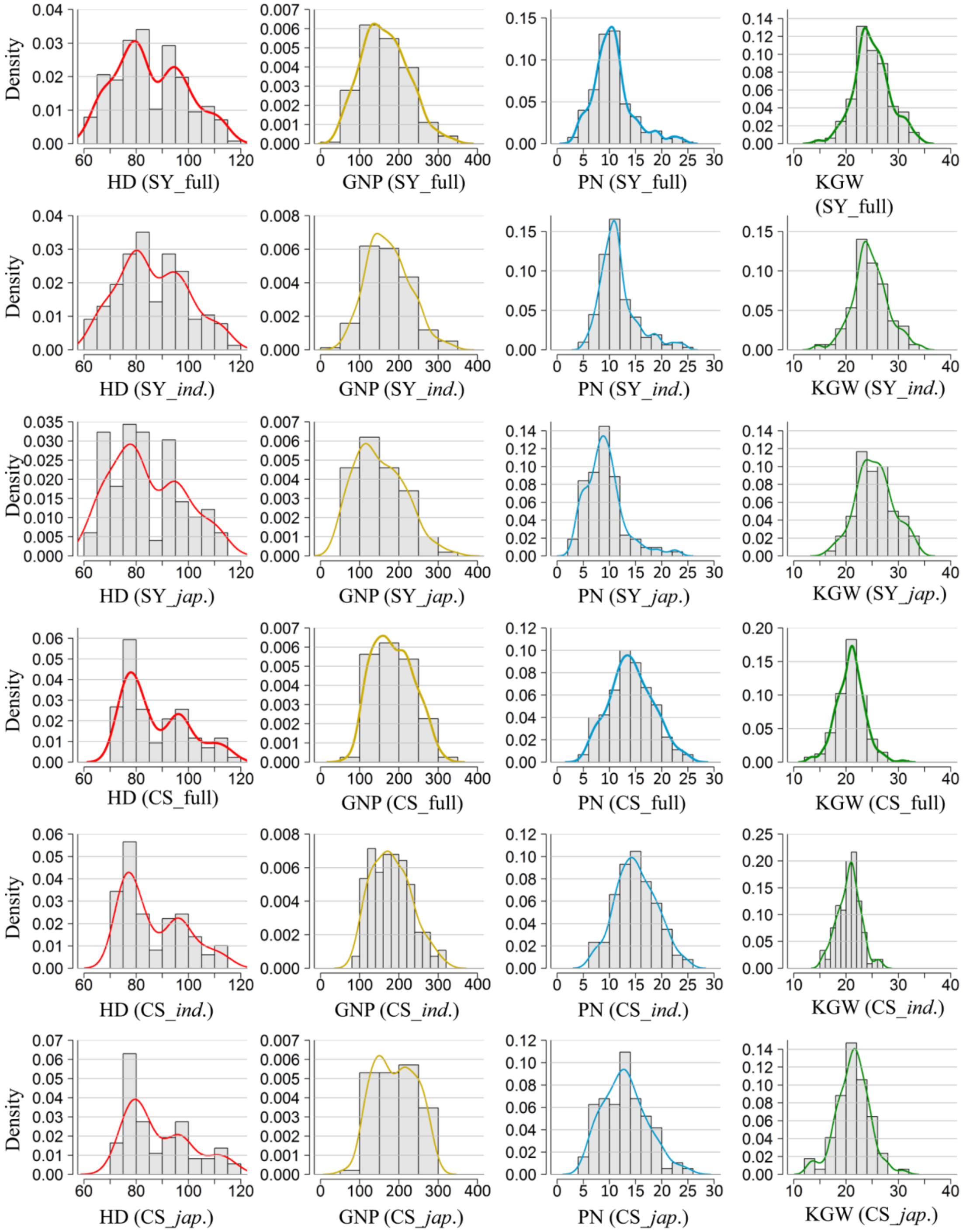

SUPPLEMENTARY FIGURE 5. Phenotypic distributions of HD, GNP, PN and KGW for full (full), *indica* (*ind*.) and *japonica* (*jap*.) populations at SY and CS.
