# Supplementary material for: Genetic Basis Underlying Correlations Among Growth Duration and Yield Traits Revealed by GWAS in Rice (Oryza sativa L.)
Source: Front Plant Sci. 2018 May 22;9:650. doi: 10.3389/fpls.2018.00650 (PMC5972282; doi:10.3389/fpls.2018.00650)
Supplement: Supplementary file 21 [file Image_7.pdf]

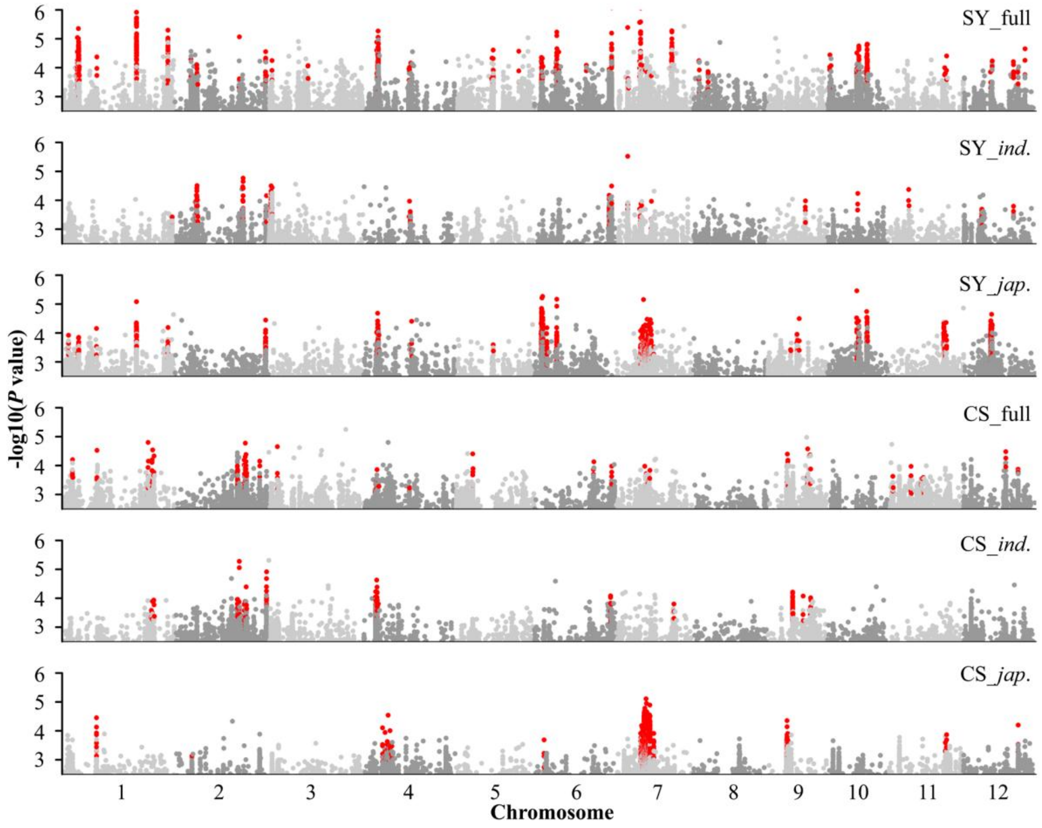

SUPPLEMENTARY FIGURE 7. Manhattan plots of GWAS for GNP in full (full), *indica* (*ind.*) and *japonica* (*jap.*) populations at SY and CS. Red dots indicate $-\log_{10}$ (P value) of SNPs in GWAS when higher than the highest $-\log_{10}$ (P value) in 1000 permutations.
