# Supplementary material for: Genetic Basis Underlying Correlations Among Growth Duration and Yield Traits Revealed by GWAS in Rice (Oryza sativa L.)
Source: Front Plant Sci. 2018 May 22;9:650. doi: 10.3389/fpls.2018.00650 (PMC5972282; doi:10.3389/fpls.2018.00650)
Supplement: Supplementary file 24 [file Image_10.pdf]

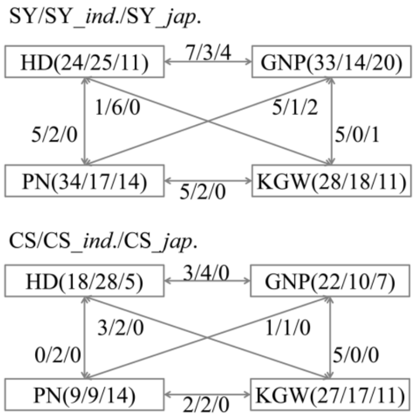

SUPPLEMENTARY FIGRUE 10. Numbers of QTLs for four traits and pleiotropic QTLs among those traits in all (the first number on the left), *indica* (*ind.*, the number between slashes) and *japonica* (*jap.*, the first number on the right) varieties grown at SY and CS.
