# Supplementary material for: Genetic Basis Underlying Correlations Among Growth Duration and Yield Traits Revealed by GWAS in Rice (Oryza sativa L.)
Source: Front Plant Sci. 2018 May 22;9:650. doi: 10.3389/fpls.2018.00650 (PMC5972282; doi:10.3389/fpls.2018.00650)
Supplement: Supplementary file 25 [file Image_11.pdf]

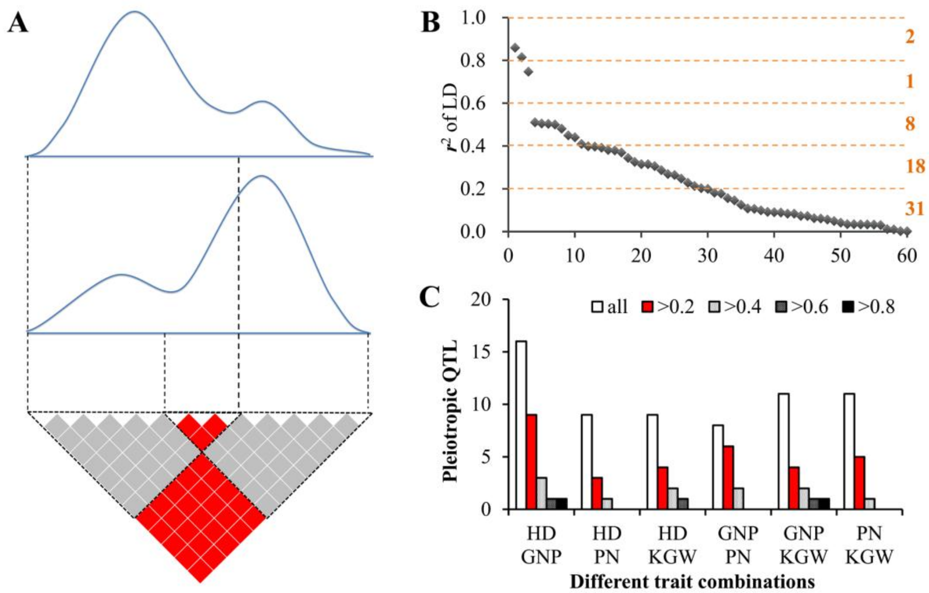

SUPPLEMENTARY FIGURE 11. Simulated diagram for calculation of linkage disequilibrium (LD) in pleiotropic QTLs and distribution of the results. (A) Calculating sketch of average r^2 (LD) in pleiotropic QTLs. Blue curves represent QTLs for different traits; red section of LD heat map participated in calculation of average r^2 (LD). **(B)** Distribution of r^2 (LD) after sequencing all pleiotropic QTLs in same population and environment. The right orange fonts show the number pleiotropic QTL at different levels of r^2 (LD). **(C)** Number of pleiotropic QTLs at different levels of r^2 (LD).
