# Supplementary material for: Genetic Basis Underlying Correlations Among Growth Duration and Yield Traits Revealed by GWAS in Rice (Oryza sativa L.)
Source: Front Plant Sci. 2018 May 22;9:650. doi: 10.3389/fpls.2018.00650 (PMC5972282; doi:10.3389/fpls.2018.00650)
Supplement: Supplementary file 26 [file Image_12.pdf]

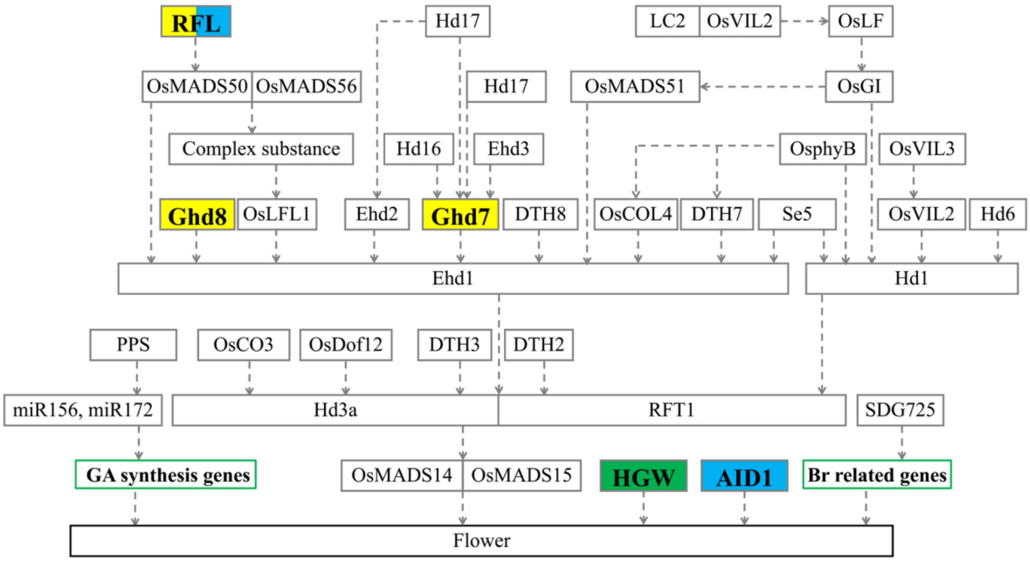

SUPPLEMENTARY FIGURE 12. Cloned genes for HD. Genes in textboxes with yellow, blue and green background are pleiotropic genes that regulated GNP, PN and KGW, respectively, but not HD; genes in boxes with green borders represent hormones.
