# Supplementary material for: Genetic Basis Underlying Correlations Among Growth Duration and Yield Traits Revealed by GWAS in Rice (Oryza sativa L.)
Source: Front Plant Sci. 2018 May 22;9:650. doi: 10.3389/fpls.2018.00650 (PMC5972282; doi:10.3389/fpls.2018.00650)
Supplement: Supplementary file 27 [file Image_13.pdf]

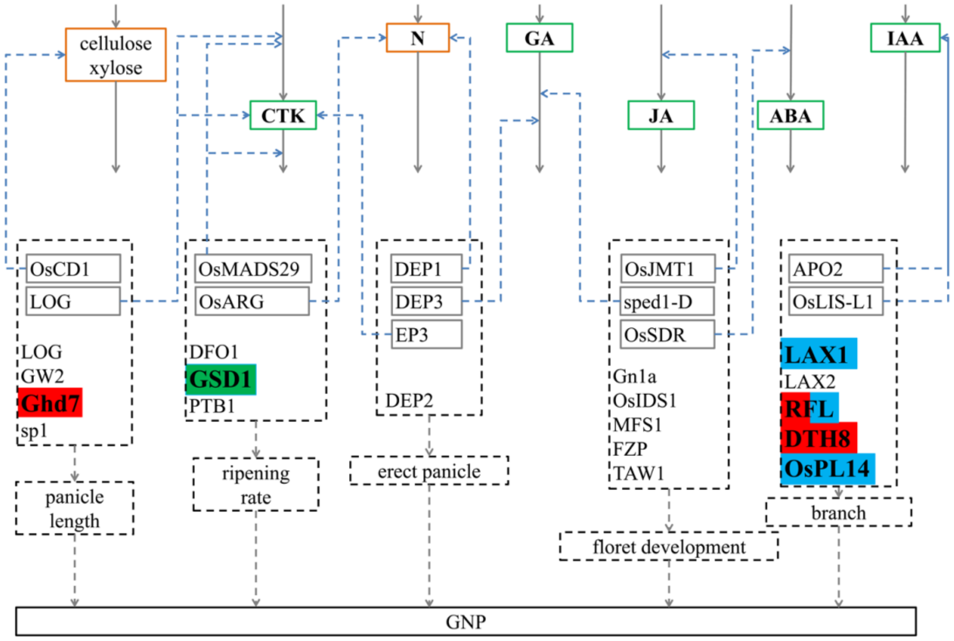

SUPPLEMENTARY FIGURE 13. Cloned genes for GNP. Genes in textboxes with red, blue and green background are pleiotropic genes; they also regulated HD, PN and KGW, respectively, except GNP; genes in boxes with green borders are hormones; those in boxes with orange borders are for N and cellulose; those in boxes with black dotted borders were classified according to morphological components, N, cellulose and hormones.
