# Supplementary material for: Genetic Basis Underlying Correlations Among Growth Duration and Yield Traits Revealed by GWAS in Rice (Oryza sativa L.)
Source: Front Plant Sci. 2018 May 22;9:650. doi: 10.3389/fpls.2018.00650 (PMC5972282; doi:10.3389/fpls.2018.00650)
Supplement: Supplementary file 29 [file Image_15.pdf]

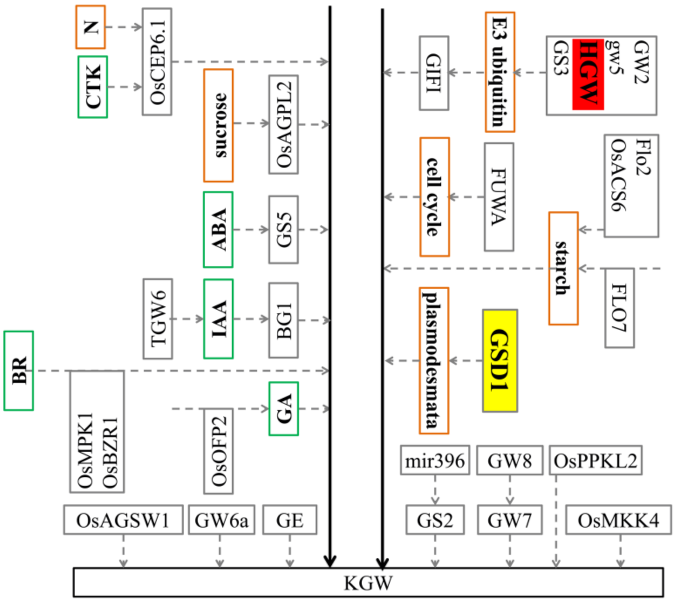

SUPPLEMENTARY FIGURE 15. Cloned genes for KGW. Genes in textboxes with red and yellow background are pleiotropic genes; they also regulated HD and GNP, respectively, but not KGW; genes in boxes with green borders are hormones; those in boxes with orange borders are energy-related and others; the two black bold arrows are fundamental carriers of KGW (last box).
