# Supplementary material for: Genetic Basis Underlying Correlations Among Growth Duration and Yield Traits Revealed by GWAS in Rice (Oryza sativa L.)
Source: Front Plant Sci. 2018 May 22;9:650. doi: 10.3389/fpls.2018.00650 (PMC5972282; doi:10.3389/fpls.2018.00650)
Supplement: Supplementary file 28 [file Image_14.pdf]

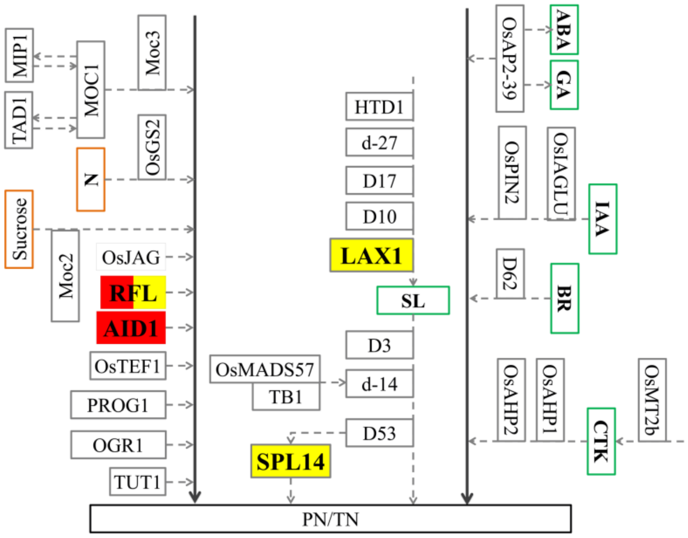

SUPPLEMENTARY FIGURE 14. Cloned genes for PN/tiller number. Genes in textboxes with red and yellow backgrounds are pleiotropic genes; they also regulated HD and GNP, respectively, but not PN/TN; genes in boxes with green borders are hormones; those in boxes with orange borders are energy-related; the two black bold arrows are fundamental carriers of PN/TN (final box).
